# Comparative Metabolic Profiling in Pulp and Peel of Green and Red Pitayas (*Hylocereus polyrhizus* and *Hylocereus undatus*) Reveals Potential Valorization in the Pharmaceutical and Food Industries

**DOI:** 10.1155/2021/6546170

**Published:** 2021-03-12

**Authors:** Xing'e Lin, Hongmao Gao, Zheli Ding, Rulin Zhan, Zhaoxi Zhou, Jianhong Ming

**Affiliations:** Haikou Experimental Station, Chinese Academy of Tropical Agricultural Sciences (CATAS), China

## Abstract

Pitaya (*Hylocereus* genus) is a popular plant with exotic and nutritious fruit, which has widespread uses as a source of nutrients and raw materials in the pharmaceutical industry. However, the potential of pitaya peel as a natural source of bioactive compounds has not yet fully been explored. Recent advances in metabolomics have paved the way for understanding and evaluating the presence of diverse sets of metabolites in different plant parts. This study is aimed at exploring the diversity of primary and secondary metabolites in two commercial varieties of pitaya, i.e., green pitaya (*Hylocereus undatus*) and red pitaya (*Hylocereus polyrhizus*). A total of 433 metabolites were identified using a widely targeted metabolomic approach and classified into nine known diverse classes of metabolites, including flavonoids, amino acids and its derivatives, alkaloids, tannins, phenolic acids, organic acids, nucleotides and derivatives, lipids, and lignans. Red pitaya peel and pulp showed relatively high accumulation of metabolites viz. alkaloids, amino acids and its derivatives, and lipids. Differential metabolite landscape of pitaya fruit indicated the presence of key bioactive compounds, i.e., L-tyrosine, L-valine, DL-norvaline, tryptophan, *γ*-linolenic acid, and isorhamnetin 3-O-neohesperidoside. The findings in this study provide new insight into the broad spectrum of bioactive compounds of red and green pitaya, emphasizing the valorization of the biowaste pitaya peel as raw material for the pharmaceutical and food industries.

## 1. Introduction

Pitaya (*Hylocereus* genus) is an important exotic fruit native to Central America and Mexico, usually cultivated in tropical and subtropical habitats [1]. Pitaya species have several cultivated types with different peel and pulp colors [2]. Classification of pitayas in different species is mainly based on pulp colors such as white (*Hylocereus undatus*), red (*H. costaricensis* and *H. polyrhizus*), and yellow (*Selenicereus megalanthus*) [3, 4]. *H. undatus* and *H. polyrhizus* are the most widely cultivated species [5]. Besides its exotic appearance and striking colors, pitaya fruit is well known for its nutritional properties and health benefits [2, 6–8]. Pitaya produces an array of chemically and biologically diverse compounds, including betanin [9, 10], phyllocactin [11], betanidin [12], gluconic acid, tyrosine [13], tricarboxylated hylocerenin, and decarboxylated neohylocerenin [2]. These metabolites make the pitaya fruit an important diet source with high nutritional value, energy, and other health benefits like chemoprevention [14, 15], anti-inflammatory [16], antimicrobial [17], antidiabetic, antioxidant properties [18–20], and prebiotic effects [16, 21].

Previous studies demonstrated the availability of secondary metabolites in pitaya fruit pulp, i.e., antioxidants [2, 6, 22], polyphenolic compounds, organic acids, amino acids, and flavonoids [2, 23]. *H. polyrhizus* seed oil is also a rich source of fatty acids, i.e., linoleic acid, that is beneficial to human health [24, 25]. However, the concentration of these metabolites varies in different fruit parts and is highly dependent on cultivars, environment, and management practices [26]. Usually, pitaya peel is discarded as biowaste during processing or direct consumption, approximately 22% of the fruit weight [27]. Pitaya fruit peel contains soluble and insoluble fiber, betalains, polyphenolic compounds, and other volatile compounds [28–30]. It has been emphasized that the concentration of metabolites tends to be significantly higher in discarded fruit parts, i.e., peel and seed, than edible parts [31]. However, not much work has been done to explore the spectrum and concentration of bioactive metabolites in different parts of pitaya fruits, i.e., peel and seeds. The scenarios of increasing yield potential and industrial demand for natural bioactive compounds have raised prospects for extending the utilization of this crop with the valorization of neglected parts of the fruit, i.e., peel and seed as previously demonstrated in citrus, apple, mango, pineapple, and pomegranate [31–33].

The advance in the field of metabolomics has facilitated the analysis and characterization of the metabolome in various plant organs, subsequently helps in the detection of thousands of bioactive metabolites [34–39]. The diversity of metabolites accumulation and metabolic profiling of different fruit plants provides a promising approach to understand and determine the commercial importance of the target fruit plants [39].

In this study, we used the widely targeted metabolomic approach to comparatively and comprehensively investigate the complex and distinct landscapes of metabolites in two main parts of pitaya fruits (peel and pulp) of the two major commercial varieties of pitaya viz. green pitaya (*Hylocereus undatus*) and red pitaya (*H. polyrhizus*). Our work provides essential basic information for the valorization of pitaya fruit peel for its use as a functional food or a potential source for raw material in the pharmaceutical industry. Further, it will be helpful to dissect the biosynthetic pathways of important metabolites in pitaya fruit.

## 2. Materials and Methods

### 2.1. Plant Materials

In this study, we used two commercial varieties, i.e., green pitaya (*Hylocereus undatus*) and red pitaya (*H. polyrhizus*) collected from Sanya fruit Island, Dragon fruit planting base, Sanya, Hainan, China. Thirty days after pollination, five fruits per tree were harvested from three different trees of each variety, representing the three replications. Pulp and peel samples from each tree were mixed separately, and all samples were immediately placed in liquid nitrogen and stored at –80°C until use.

### 2.2. Metabolomic Analyses

The sample preparation, extract analysis, metabolite identification, and quantification were performed at Wuhan Metware Biotechnology Co., Ltd., Wuhan, China, following their standard procedures.

### 2.3. Sample Preparation

All samples were ground to a fine powder using a Grinding Mill at 65 Hz for 90 s. A total of 50 mg of sample was weighed and extracted with 800 *μ*L of methanol. The samples were vortexed for 30 s and centrifuged at 12000 rpm and 4°C for 15 min. 200 *μ*L of supernatant was transferred to the vial for LC-MS analysis.

### 2.4. Liquid Chromatography Coupled Mass Spectrometry (LC-MS)

The data acquisition instrument system included LC-MS (Thermo, Ultimate 3000LC, Orbitrap Elite). The liquid phase conditions included (1) column: Hypergod C18 (100 × 4.6 mm 3 *μ*m); (2) mobile phase: phase A = water + 0.1%formic acid and phase B = acetonitrile + 0.1%formic acid; (3) elution gradient: 0 min B = 5%in comparison and 2 min B = 5%, B was linearly increased to 95% in 12 min and maintained at 95% for 15 min, and B was reduced to 5% and was balanced to 17 min; and (4) flow rate 0.3 mL/min: injection volume = 4 *μ*L and automatic injector temperature = 4°C. Whereas the MS conditions were as follows: the positive electrospray ionization (ESI+) temperature was 300°C, sheath gas flow rate was 45 arb, aux gas flow rate was 15 arb, sweep gas flow rate was 1 arb, spray voltage was 3.0 kV, the capillary temperature was 350°C, and S-Lens RF level was 30%. The ESI-conditions were the same as ESI+ except that the spray voltage was 3.2 kV, and the S-Lens RF level was 60%. The qualitative analysis of the material was established on secondary spectrum information using the public databases (KNApSAcK, METLIN, LipidMAps, and MassBank) of metabolites. The isotope signals were removed during the quantitative analysis of samples. Triple Q scans were attained as multiple reaction monitoring (MRM) experiments. Declustering potential (DP) and collision energy (CE) for individual MRM transitions were done with further DP and CE optimization [40]. A specific set of MRM transitions was monitored for each period according to the metabolites eluted within this period.

### 2.5. Quality Control and Data Analysis

Quality control was performed to check the reliability and reproducibility of the data. Extracted samples were mixed and inserted into every four samples, and changes were monitored. Data sets with the intensity of the metabolites from each sample, i.e., peel and pulp, were uploaded to the Analyst 1.6.1 software (AB SCIEX, Ontario, ON, Canada) for descriptive statistical analyses. The principal component analysis was performed using R package prcomp and visualized using ggbiplot.

## 3. Results

### 3.1. Metabolome Profiling of the Pitaya Varieties

**Figure 1:**
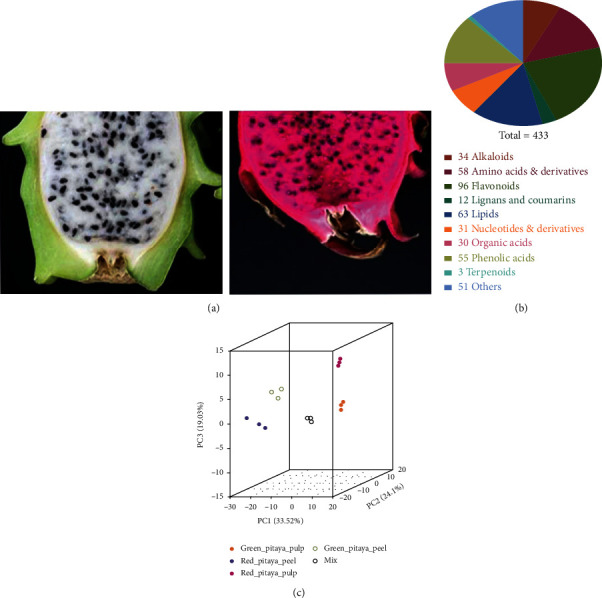
Metabolome landscape in peel and pulp tissue of red pitaya (*Hylocereus polyrhizus*) and green pitaya (*Hylocereus undatus*) varieties: (a) pictorial description of green and red pitaya fruit; (b) distribution of identified metabolites classes; (c) principal component analysis with 3D visualization, comprising PC1 (33.52%), PC2 (24.1%), and PC3 (19.03%).

Two commercial pitaya varieties, green pitaya (*Hylocereus undatus*) and red-pitaya (*H. polyrhizus*), were used for metabolomic analyses. These two commercial varieties possess distinguished morphological appearances of peel and pulp (Figure 1(a)). Significant contrasting fruit colors suggested the variable concentration of metabolites in peel and pulp tissues. To support this hypothesis, in this study, we examined the metabolome profile in the peel and pulp tissues of both cultivars. Ultraperformance liquid chromatography and tandem mass spectrometry (UPLC-MS/MS) techniques were employed and identified in total of 443 metabolites (Table S1). Following the recommendations of international metabolomics society (http://metabolomicssociety.org/), the identified metabolites were categorized into first two levels A (identified metabolites) and B (putatively annotated metabolites). Five random samples were selected, and their graphs of mass scan data collected over time (TIC) have been presented in supplementary figures 1-5.The identified metabolites were further classified into nine known major classes based on metabolites structure (Figure 1(b)). Among these 443 metabolites, 96 flavonoids, 63 lipids, 58 amino acids and derivatives, 55 phenolic acids, 51 others, 34 alkaloids, 31 nucleotide and derivatives, 30 organic acids, 12 lignans, and coumarins, and 3 terpenoids were included (Figure 1(b)). These results indicate that flavonoids, lipids, amino acids and derivatives, and phenolic acids are in high proportion in pitaya. Detailed information about the identified landscape of metabolites, including molecular weights (Da), formula, compounds, major and minor classes, ionization, and KEGG maps, is described in Table S1.

Principle component analysis (PCA) was performed to summarize the descriptive assessment, help to understand the underlying characteristics and structure of the metabolome data. The 3D PCA plot in Figure 1(c) demonstrated the four distinctive structures of metabolome data based on tissue and variety. Mix was used for quality control in the analysis. PCA plot illustrated the closer grouping of red pitaya peel to that green pitaya peel, similar to red and green pitaya pulp samples. These results suggested the close grouping among the fruit tissues rather than varieties, and all replicates were grouped together, suggesting a high quality of our metabolome profiling. Metabolome variance explained by the first three PCA coordinates accumulatively higher as 76.65%. First PCA coordinate (PC1) explained 33.52%, PC2 explained 24.1%, and PC3 explained 19.03% variance of metabolome data. These results indicated the high variance of metabolite concentrations in pitaya fruit.

### 3.2. Metabolite Landscape in Pitaya Fruit Peel from the Two Varieties

**Figure 2:**
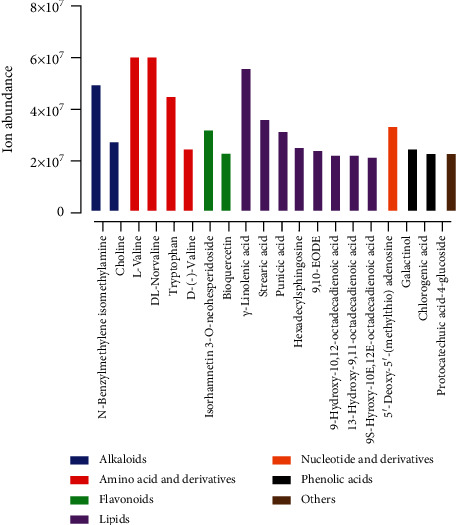
Top 20 most abundant metabolites in pitaya fruit peel.

The metabolome landscape of pitaya fruit peel revealed a diverse array of metabolites. Top 20 metabolites identified based on metabolites ion abundance were N-benzylmethylene isomethylamine, choline, L-valine, DL-norvaline, tryptophan, D-(-)-valine, isorhamnetin 3-O-neohesperidoside, bioquercetin, *γ*-linolenic acid, stearic acid, punicic acid, hexadecylsphingosine, 9,10-EODE, 9-hydroxy-10,12-octadecadienoic acid, 13-hydroxy-9,11-octadecadienoic acid, 9S-hyroxy-10E,12E-octadecadienoic acid, 5′-deoxy-5′-(methylthio) adenosine, galactinol, chlorogenic acid, and protocatechuic acid-4-glucoside (Figure 2). Of these, choline (6.03*E* + 07), L-valine (5.99*E* + 07), *γ*-linolenic acid (5.56*E* + 07), and N-benzylmethylene isomethylamine (4.90*E* + 07) were the most concentrated metabolites in fruit peel (Table S2). These top 20 metabolites belong to seven major classes, i.e., alkaloids, amino acid and derivatives, flavonoids, lipids, nucleotide and derivatives, phenolic acids, and others. Lipids and amino acids and derivatives were in higher frequency compared to remaining metabolite classes.

### 3.3. Metabolite Landscape in Pitaya Fruit Pulp from the Two Varieties

**Figure 3:**
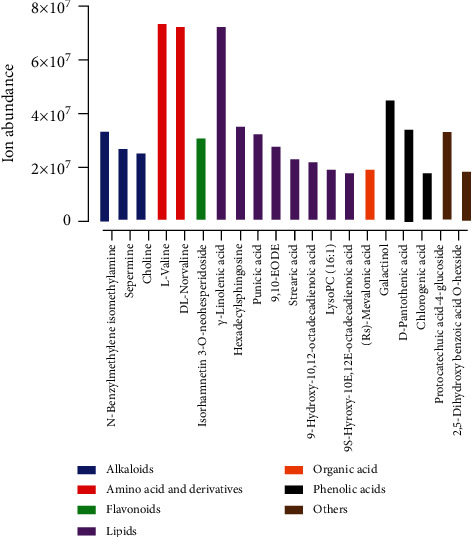
Top 20 most abundant metabolites in fruit pulp.

Metabolome landscape was investigated in fruit pulp of green pitaya (*Hylocereus undatus*) and red pitaya (*Hylocereus polyrhizus*) varieties. The top 20 most profuse metabolites identified in fruit pulp were N-benzylmethylene isomethylamine, spermine, choline, L-valine, DL-norvaline, isorhamnetin 3-O-neohesperidoside, *γ*-linolenic acid, hexadecylsphingosine, punicic acid, 9,10-EODE, stearic acid, 9-hydroxy-10,12-octadecadienoic acid, LysoPC (16 : 1), 9S-hyroxy-10E,12E-octadecadienoic acid, (Rs)-mevalonic acid, galactinol, D-pantothenic acid, chlorogenic acid, protocatechuic acid-4-glucoside, and 2,5-dihydroxy benzoic acid O-hexside (Figure 3). Of these metabolites, L-valine (7.33*E* + 07), *γ*-linolenic acid (7.21*E* + 07), and DL-norvaline (7.20*E* + 07) revealed the highest ion abundance (Table S3). These top 20 metabolites of fruit pulp also belonged to seven major classes, i.e., alkaloids, amino acid and derivatives, flavonoids, lipids, organic acid, phenolic acids, and others. While metabolites belonging to lipids, alkaloids, and phenolic acid classes had higher occurrences than other metabolite classes.

### 3.4. Comparative Analysis of Metabolome from the Pitaya Fruit Peel

**Figure 4:**
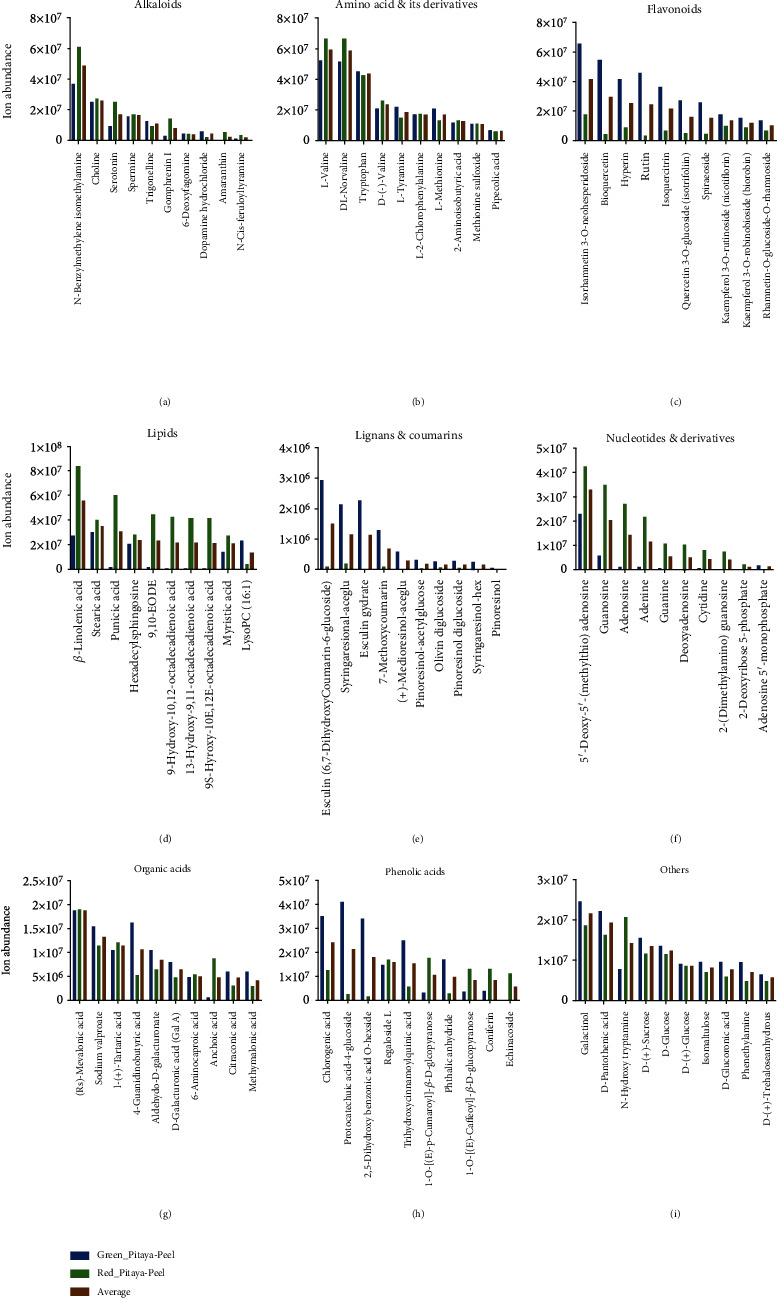
Comparative analysis between red pitaya peel and green pitaya peel based on the top 10 metabolites from each class: (a) alkaloids; (b) amino acid and derivatives; (c) flavonoids; (d) lipids; (e) lignans and coumarins; (f) nucleic acid and derivatives; (g) organic acids; (h) phenolic acids; (i) others.

For further investigation, comparative analyses between the two varieties were performed based on the most abundant metabolites of nine major metabolite classes from each tissue separately. The top 10 highest abundant metabolites from each class in fruit peel were selected and compared between the two varieties (Figure 4). In the present study, 34 metabolites belonged to the alkaloid class. Alkaloid is one of the most important secondary metabolite classes which naturally occurred in plants [41]. The top 10 most accumulated metabolites identified in fruit peel tissues were N-benzylmethylene isomethylamine, choline, serotonin, spermine, trigonelline, gomphrenin I, 6-deoxyfagomine, dopamine hydrochloride, amaranthin, and N-cis-feruloyltyramine (Figure 4(a)). N-Benzylmethylene isomethylamine metabolite showed the highest ion abundance among the top 10 alkaloids. N-Benzylmethylene isomethylamine and serotonin indicated significantly higher ion abundance in red pitaya compared to green pitaya varieties. The top 10 abundant amino acids and derivatives found in fruit peel were L-valine, DL-norvaline, tryptophan, D-(-)-valine, L-tyramine, L-2-chlorophenylalanine, L-methionine, 2-aminoisobutyric acid, methionine sulfoxide, and pipecolic acid (Figure 4(b)). L-Valine, DL-norvaline, and tryptophan metabolites revealed the highest ion abundance among the top 10 abundant amino acids and derivatives. L-Valine and DL-norvaline showed significantly higher ion accumulation in red pitaya peel than green pitaya peel. Further, flavonoids were screened based on the average value of ion abundance in green pitaya peel and red pitaya peel, and we selected the top 10 most abundant metabolites. These metabolites were isorhamnetin 3-O-neohesperidoside, bioquercetin, hyperin, rutin, isoquercitrin, quercetin 3-O-glucoside (Isotrifoliin), spiraeoside, kaempferol 3-O-rutinoside (Nicotiflorin), kaempferol 3-O-robinobioside (Biorobin), and rhamnetin-O-glucoside-O-rhamnoside (Figure 4(c)). These top 10 flavonoids revealed significantly higher ion abundance in green pitaya peel compared to red pitaya peel. Lipid class was also evaluated for the top 10 most abundant metabolites based on ion abundance average of red and green pitaya peels. The top 10 most abundant metabolites were *γ*-linolenic acid, stearic acid, punicic acid, hexadecylsphingosine, 9,10-EODE, 9-hydroxy-10,12-octadecadienoic acid, 13-hydroxy-9,11-octadecadienoic acid, 9S-hyroxy-10E,12E-octadecadienoic acid, myristic acid, and LysoPC (16 : 1) (Figure 4(d)). All metabolites except LysoPC (16 : 1) displayed higher ion accumulation in red pitaya fruit compared to green pitaya fruit. Lignans and coumarins class were screened for the top 10 metabolites, and we identified esculin (6,7-dihydroxycoumarin-6-glucoside), syringaresinol-aceGlu, esculin hydrate, 7-methoxycoumarin, (+)-medioresinol-aceGlu, pinoresinol-acetylglucose, olivin diglucoside, pinoresinol diglucoside, syringaresinol-Hex, and pinoresinol metabolites (Figure 4(e)). The first three metabolites showed the highest concentrations. While lignans and coumarins, organic acids (Figure 4(g)), phenolic acids (Figure 4(h)), and other (Figure 4(i)) class metabolites indicated similar behavior to flavonoid class as almost all top 10 metabolites exhibited significantly higher abundance in green pitaya than the red pitaya varieties, while nucleotides and derivatives displayed the opposite pattern (Figure 4(f)).

### 3.5. Comparative Analysis of Metabolome from Pitaya Fruit Pulp

**Figure 5:**
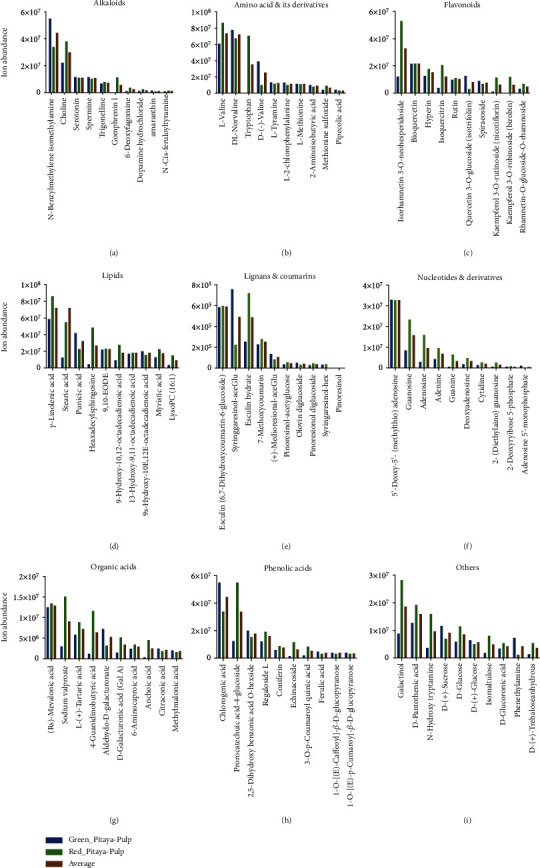
Comparative analysis between red pitaya pulp and green pitaya pulp based on top 10 metabolites from each class: (a) alkaloids; (b) amino acid and derivatives; (c) flavonoids; (d) lipids; (e) lignans and coumarins; (f) nucleic acid and derivatives; (g) organic acids; (h) phenolic acids; (i) others.

To understand the important metabolites from each class in fruit pulp, the top 10 abundant metabolites were selected and compared between red pitaya pulp and green pitaya pulp. Firstly, metabolites of the alkaloid class were evaluated for the top 10 most abundant metabolites. These metabolites were N-benzylmethylene isomethylamine, choline, serotonin, spermine, trigonelline, gomphrenin I, 6-deoxyfagomine, dopamine hydrochloride, amaranthin, and N-cis-feruloyltyramine (Figure 5(a)). N-Benzylmethylene isomethylamine, choline, and serotonin were the most abundant in pitaya fruit pulp. Choline metabolite demonstrated higher ion accumulation in red pitaya pulp than the green pitaya pulp. In contrast, serotonin showed higher ion accumulation in green pitaya pulp compared to red pitaya pulp. Amino acid and derivative classes were screened for the most abundant metabolites. Top 10 metabolites, L-valine, DL-norvaline, tryptophan, D-(-)-valine, L-tyramine, L-2-chlorophenylalanine, L-methionine, 2-aminoisobutyric acid, methionine sulfoxide, and pipecolic acid, were selected (Figure 5(b)). The first two metabolites, L-valine and DL-norvaline, demonstrated higher ion accumulation compared to the remaining metabolites. These metabolites also showed higher ion abundance in red pitaya pulp compared to green pitaya pulp. Flavonoids are also among the important metabolite class. The top 10 abundant metabolites from the flavonoid class were selected as isorhamnetin 3-O-neohesperidoside, bioquercetin, hyperin, rutin, isoquercitrin, quercetin 3-O-glucoside (Isotrifoliin), spiraeoside, kaempferol 3-O-rutinoside (Nicotiflorin), kaempferol 3-O-robinobioside (Biorobin), and rhamnetin-O-glucoside-O-rhamnoside (Figure 5(c)). Isorhamnetin 3-O-neohesperidoside metabolite revealed higher ion accumulation among the flavonoid class and also revealed higher abundance in red pitaya pulp compared to green pitaya pulp. Lipid and nucleotides and derivative classes were screened for the top 10 metabolites, as shown in Figures 5(d) and 5(f), respectively. Metabolites from both of these classes demonstrated higher ion abundance in red pitaya pulp compared to green pitaya pulp. Further, metabolites from lignans and coumarins (Figure 5(e)) and phenolic acids (Figure 4(h)) indicated contrasting behavior. Few metabolites showed higher ion abundance in green pitaya pulp, while few demonstrated higher ion accumulation in red pitaya pulp. Besides, organic acids (Figure 4(g)) and other (Figure 4(i)) class metabolites demonstrated significantly higher ion abundance in red pitaya pulp than green pitaya pulp.

### 3.6. Comparative Analysis of Metabolome between Fruit Pulp and Peel

**Figure 6:**
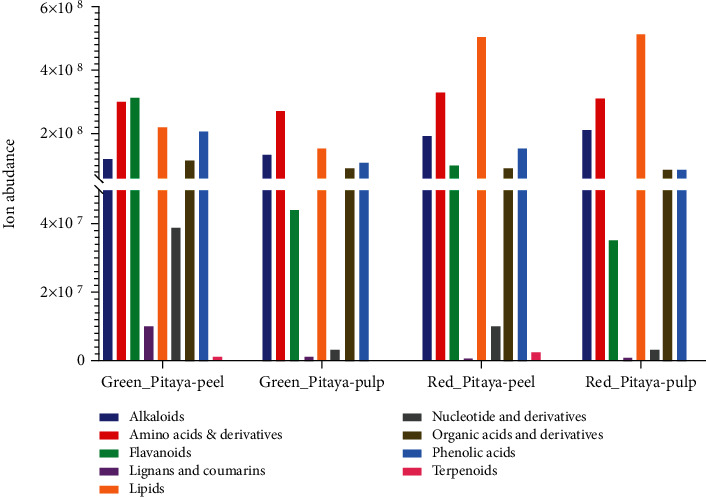
Sum total of metabolite classes in different tissues, i.e., peel and pulp of green pitaya (*Hylocereus undatus*) and red pitaya (*Hylocereus polyrhizus*) fruit.

Ion abundance of major classes in peel and pulp tissues was compared among the two commercial cultivars, as shown in Figure 6. Comparative analysis revealed that peel tissues of both cultivars have higher metabolite abundance compared to the pulp tissues. Moreover, red pitaya varieties demonstrated higher ion abundance in peel and pulp compared to green pitaya varieties. Overall, metabolite results indicated that few metabolite classes, i.e., amino acid and derivatives, alkaloids, lipids, organic acid and derivatives, and phenolic acids, were most abundant in peel and pulp tissues. In contrast, lipids demonstrated higher accumulation in red pitaya (*Hylocereus polyrhizus*) fruits. Furthermore, flavonoids exhibited tissue-specific patterns of ion abundance as this class of metabolites was enriched in peel tissues compared to pulp tissues. N-Benzylmethylene isomethylamine, choline, L-valine, DL-norvaline, isorhamnetin 3-O-neohesperidoside, *γ*-linolenic acid, punicic acid, stearic acid, 9-hydroxy-10,12-octadecadienoic acid, LysoPC (16 : 1), 9S-hyroxy-10E,12E-octadecadienoic acid, galactinol, chlorogenic acid, and protocatechuic acid-4-glucoside showed higher abundance in both peel and pulp tissues (Figures 2 and 3, Table S2 and 3). However, tryptophan, D-(-)-valine, bioquercetin, hexadecylsphingosine, 9,10-EODE, 13-hydroxy-9,11-octadecadienoic acid, and 5′-deoxy-5′-(methylthio) adenosine showed higher abundance in peel tissues.

## 4. Discussion

It has been documented that discarded fractions of fruits contain the highest accumulation of bioactive compounds [26, 42]. However, comparative metabolic profiling of pitaya peel and pulp in different cultivars is lacking. Such a study will provide insight into the importance of discarded parts, which in turn will help valorize the pitaya crop for its nutritional and industrial utilization. Keeping this in view, the present study was performed to evaluate the metabolic profiles of pitaya peel and pulp tissues. Previous studies revealed that red pitaya cultivars are rich in amino acids like L- and D- amino acids [43]. A recent increase in synthetic coloration in the food industry is becoming a significant concern for scientists due to its adverse effects on human health [44]. Recently, albedo parts of pitaya fruit were taken to form a coloring powder as a natural food additive [44], in addition to previous utilization of pitaya as a natural colorant for the food industry [45, 46]. Betalain, a secondary metabolite, is derived for L-tyrosine with its known use as natural food colorant [47]. Although L-tyrosine was not among the most abundant metabolites in our study, we identified a significant abundance of L-tyrosine in both red and green pitaya (Table S1), with the highest abundance in red pitaya peel. This study also indicated the highest concentration of L- and D- amino acids like L-valine, DL-norvaline, D-(-)-valine, L-tyramine, L-2-chlorophenylalanine, and L-methionine in the peel and pulp tissues of pitaya fruit.

Pitaya fruit, with its nutritional benefits and culinary applications, is an excellent natural source for use as a raw material in the cosmetics and pharmaceutical industry [48, 49]. Large scale metabolic profiling of pitaya peel and pulp tissues indicated a diverse array of metabolites from different major metabolite classes. The top 10 most abundant metabolites are conserved in peel and pulp tissues, while their abundance varied in green pitaya and red pitaya cultivars. This metabolic profiling between the two cultivars suggests a differential pattern of metabolite abundance, as it is evident that the metabolic landscape varies according to the variety, underlying tissues, and environment [50–53]. Metabolome landscapes of peel and pulp tissues showed that alkaloids, amino acid and derivatives, lipids, organic acid and derivatives, and phenolic acids have the highest concentrations. Alkaloid class has not only important role in human diet but also is famous for its pharmaceutical properties like antioxidative [54], antibacterial [55, 56], antiparasitic [57], insecticidal [58], anticorrosive [59], and antiplasmodial [59]. N-Benzylmethylene isomethylamine, spermine, and choline were the most abundant alkaloids estimated in this study which have potential pharmaceutical applications [60, 61]. Protein extricates from plants are the essential raw material in the cosmetic and pharmaceutical industries [62, 63]. Peel extracts of pitaya fruit unveiled a noticeable abundance of amino acids, including L-valine, DL-norvaline, and tryptophan. These amino acids are well-known for their beneficial characteristics in the human body like insulin secretion, muscle strengthening, mammary health, mucin production, and several other metabolic functions [64–67]. Among the lipid class, *γ*-linolenic acid is the highest concentrated metabolite which is important for several beneficial human health effects like atopic eczema, platelet aggregation, and immune function [68–70].

Besides, isorhamnetin 3-O-neohesperidoside metabolite exhibited the highest abundances among flavonoid class in peel tissue. This metabolite is widely employed in clinical practices, i.e., scrofula, abscesses, abdominal pain, and many other chronic diseases [71, 72], and also acts as antioxidant [73], antimicrobial [74], and antiatherogenic [75]. Therapeutic effects of hexadecylsphingosine with a sphingolipid base, one of the most abundant lipid metabolite found in this study, during intestinal cancer have been documented [76]. Furthermore, another lipid metabolite, punicic acid, abundantly present in green pitaya pulp, has been reported in many studies for its therapeutic effects during different chronic diseases in human viz. obesity, diabetics, inflammation, and metabolic syndromes [77–79]. Pomegranate seed oil is considered as the primary natural source of punicic acid [77, 80]. Stearic acid from lipids was also found to be one of the highly abundant metabolites in pulp tissues of red pitaya pulp. Previous reports suggested an active role of stearic acid in lowering cholesterol levels in humans [81, 82]. Phenolics are natural compounds involved in potential health benefits, as they possess cardioprotective, anti-inflammatory, antiallergic, anticarcinogenic, antiarthritic, antioxidant, and antimicrobial activities [83]. D-Pantothenic acid was found among the most abundant phenolic acids in this study. Disulfide pantothenic acid is considered as the most active form of vitamin B_5_ [84]. Another phenolic acid, chlorogenic acid (with high abundance in green pitaya peel and pulp tissue), is considered as helpful bioactive compound against neurodegenerative conditions beside its widespread use as an antioxidant [85–87]. Presented results and previously reported statistics suggested that fruit peel, especially red pitaya peel, is a rich source of metabolites with potential applications in pharmaceutics as a natural raw material.

## 5. Conclusions

Metabolome landscape of pitaya fruit provides insight into the natural variations between peel and pulp tissues of green pitaya (Hylocereus undatus) and red pitaya (Hylocereus polyrhizus) varieties. An array of 433 metabolites was identified in pitaya fruit, which includes nine known diverse metabolite classes. Results suggested that both tissues of pitaya fruit are abundant in important metabolite classes like amino acid and derivatives and lipids. Our study also sheds light on the importance of pitaya fruit peel, which is usually discarded as a waste product. Peel and pulp of red pitaya have a high abundance of major classes; therefore, may have more health benefits and pharmaceutical importance compared to green pitaya. Further exploitation and understanding of physio-chemical properties of pitaya peel metabolome can pave the way for a better valorization of pitaya fruit as a raw material in the food and pharmaceutical industries.

## Data Availability

All data used in this work could be found inside the text and in the supplementary tables.

## Supplementary Materials

Supplementary 1 Table S1: metabolome landscape in peel and pulp tissues of green pitaya and red pitaya varieties. Table S2: top 20 most abundant metabolites in pitaya fruit peel. Table S3: top 20 most abundant metabolites in pitaya fruit pulp.

Supplementary 2 Figure S1: graph of mass scan data collected over time (TIC) of MRM from sample 20-Meta2 (9-hydroxy-10,12-octadecadienoic acid). Figure S2: graph of mass scan data collected over time (TIC) of MRM from sample 26-Meta 26 (trigonelline). Figure S3: graph of mass scan data collected over time (TIC) of MRM from sample 30-Meta30 (baicalein). Figure S4: graph of mass scan data collected over time (TIC) of MRM from sample 37-Meta37 (tangeretin). Figure S5: graph of mass scan data collected over time (TIC) of MRM from sample 43-Meta43 (isoquercitrin).
