# Supplementary material for: Comparative Metabolic Profiling in Pulp and Peel of Green and Red Pitayas (Hylocereus polyrhizus and Hylocereus undatus) Reveals Potential Valorization in the Pharmaceutical and Food Industries
Source: Biomed Res Int. 2021 Mar 12;2021:6546170. doi: 10.1155/2021/6546170 (PMC7980772; doi:10.1155/2021/6546170)
Supplement: Supplementary 1 — Table S1: metabolome landscape in peel and pulp tissues of green pitaya and red pitaya varieties. Table S2: top 20 most abundant metabolites in pitaya fruit peel. Table S3: top 20 most abundant metabolites in pitaya fruit pulp. [file 6546170.f1.docx]

**Table S1.** Metabolome landscape in peel and pulp tissues of both green-pitaya and red-pitaya cultivars

| **Index** | **Molecular Weight (Da)** | **Formula** | **Compounds** | **Class I** | **Class II** | **CAS** | **Level of identification** | **Ion fragments** | **Green_Pitaya-peel_Average** | **Green_Pitaya-pulp_Average** | **Red_Pitaya-peel_Average** | **Red_Pitaya-pulp_Average** | **Average** | **mix_quality check** |
| --- | --- | --- | --- | --- | --- | --- | --- | --- | --- | --- | --- | --- | --- | --- |
| Meta75 | 1.17E+02 | C5H11NO2 | L-Valine | Amino acids and derivatives | Amino acids and derivatives | 72-18-4 | A | 118.09, 72.08, 55.06, 57.06, 56.05 | 5.31E+07 | 6.03E+07 | 6.75E+07 | 8.63E+07 | 6.68E+07 | 7.33E+07 |
| Meta342 | 1.17E+02 | C5H11NO2 | DL-Norvaline | Amino acids and derivatives | Amino acids and derivatives | 760-78-1 | B | 118.09, 72.08, 55.06, 57.06, 56.05 | 5.26E+07 | 5.83E+07 | 6.71E+07 | 8.57E+07 | 6.59E+07 | 7.17E+07 |
| Meta314 | 2.83E+02 | C10H13N5O5 | Guanosine | Nucleotides and derivatives | Nucleotides and derivatives | 118-00-3 | A | 284.1, 152.06, 135.03, 110.03, 222.99 | 5.78E+06 | 7.73E+07 | 3.49E+07 | 6.70E+07 | 4.62E+07 | 3.40E+07 |
| Meta432 | 1.19E+02 | C8H9N | N-Benzylmethylene isomethylamine | Alkaloids | Alkaloids | - | - | - | 3.70E+07 | 3.29E+07 | 6.10E+07 | 3.26E+07 | 4.09E+07 | 4.11E+07 |
| Meta90 | 2.78E+02 | C18H30O2 | γ-Linolenic Acid | Lipids | Free fatty acids | 506-26-3 | A | 277.22, 59.01, 259.21, 233.23, 205.2 | 2.74E+07 | 4.04E+06 | 8.39E+07 | 9.40E+06 | 3.12E+07 | 5.72E+07 |
| Meta157 | 2.84E+02 | C18H36O2 | Stearic Acid | Lipids | Free fatty acids | - | - | - | 3.03E+07 | 2.22E+07 | 4.02E+07 | 2.31E+07 | 2.89E+07 | 3.04E+07 |
| Meta411 | 2.04E+02 | C11H12N2O2 | Tryptophan | Amino acids and derivatives | Amino acids and derivatives | 73-22-3 | A | 205.1, 188.1, 146.06, 118.07 | 4.57E+07 | 1.32E+07 | 4.33E+07 | 9.93E+06 | 2.80E+07 | 3.32E+07 |
| Meta171 | 1.17E+02 | C5H11NO2 | D-(-)-Valine | Amino acids and derivatives | Amino acids and derivatives | 640-68-6 | A | 118.09, 72.08, 55.06, 57.06, 56.05 | 2.13E+07 | 2.23E+07 | 2.66E+07 | 3.82E+07 | 2.71E+07 | 2.80E+07 |
| Meta332 | 1.76E+02 | C10H12N2O | Serotonin | Alkaloids | Plumerane | 50-67-9 | A | 177.1, 160.08, 132.08, 115.05, 117.06 | 9.74E+06 | 4.18E+07 | 2.54E+07 | 2.27E+07 | 2.49E+07 | 2.21E+07 |
| Meta58 | 3.54E+02 | C16H18O9 | Chlorogenic acid | Phenolic acids | Phenolic acids | 327-97-9 | B | 353.09, 191.06, 161.03, 179.04, 173.05 | 3.54E+07 | 3.94E+07 | 1.28E+07 | 1.03E+07 | 2.45E+07 | 2.95E+07 |
| Meta83 | 1.92E+02 | C6H8O7 | Citric Acid | Organic acids | Organic acids | 77-92-9 | A | 191.02, 111.01, 87.01, 85.03, 111 | 9.00E+00 | 5.51E+07 | 9.00E+00 | 3.39E+07 | 2.23E+07 | 3.67E+06 |
| Meta05 | 5.50E+02 | C24H26N2O13 | Gomphrenin I | Alkaloids | Alkaloids | 17008-59-2 | A | 551.16, 303.04, 551.16, 533.14, 287.07 | 3.18E+06 | 1.87E+04 | 1.43E+07 | 7.02E+07 | 2.19E+07 | 1.38E+07 |
| Meta422 | 2.73E+02 | C16H35NO2 | Hexadecylsphingosine | Lipids | Sphingolipids | - | - | - | 2.04E+07 | 1.25E+07 | 2.78E+07 | 2.24E+07 | 2.08E+07 | 2.15E+07 |
| Meta229 | 1.76E+02 | C10H12N2O | N-Hydroxy tryptamine | Others | Others | - | B | 177.1, 160.08, 132.08, 115.05, 117.06 | 7.60E+06 | 3.57E+07 | 2.08E+07 | 1.90E+07 | 2.08E+07 | 1.82E+07 |
| Meta324 | 2.97E+02 | C11H15N5O3S | 5'-Deoxy-5'-(methylthio)adenosine | Nucleotides and derivatives | Nucleotides and derivatives | 2457-80-9 | B | 298.1, 136.07, 61.01, 119.04, 145.03 | 2.30E+07 | 5.88E+06 | 4.26E+07 | 1.13E+07 | 2.07E+07 | 1.84E+07 |
| Meta232 | 5.17E+02 | C26H48NO7P | LysoPC 18:3 | Lipids | LPC | - | A | 518.32, 184.08, 104.11, 500.31, 258.11 | 7.56E+06 | 1.28E+07 | 7.27E+06 | 5.47E+07 | 2.06E+07 | 1.80E+07 |
| Meta237 | 5.17E+02 | C26H48NO7P | LysoPC 18:3(2n isomer) | Lipids | LPC | - | A | 518.32, 184.08, 104.11, 500.31, 258.11 | 7.11E+06 | 1.21E+07 | 6.72E+06 | 5.30E+07 | 1.97E+07 | 1.69E+07 |
| Meta125 | 1.99E+02 | C9H10ClNO2 | L-2-chlorophenylalanine | Amino acids and derivatives | Amino acids and derivatives | - | - | - | 1.74E+07 | 2.13E+07 | 1.80E+07 | 2.18E+07 | 1.96E+07 | 1.98E+07 |
| Meta207 | 1.03E+02 | C5H13NO | Choline | Alkaloids | Alkaloids | - | - | - | 2.55E+07 | 1.33E+07 | 2.77E+07 | 1.16E+07 | 1.95E+07 | 2.08E+07 |
| Meta29 | 2.02E+02 | C10H26N4 | Spermine | Alkaloids | Phenolamine | - | - | - | 1.62E+07 | 1.72E+07 | 1.73E+07 | 1.83E+07 | 1.72E+07 | 1.72E+07 |
| Meta381 | 6.10E+02 | C27H30O16 | Bioquercetin | Flavonoids | Flavonols | 52525-35-6 | A | 609.15, 609.14, 301.04, 300.03, 607.25 | 4.11E+07 | 1.15E+07 | 3.08E+06 | 1.08E+07 | 1.66E+07 | 1.49E+07 |
| Meta240 | 2.78E+02 | C18H30O2 | Punicic acid | Lipids | Free fatty acids | 544-72-9 | A | 277.22, 59.01, 259.21, 233.23, 205.2 | 1.62E+06 | 9.85E+05 | 5.99E+07 | 1.67E+06 | 1.60E+07 | 2.17E+07 |
| Meta426 | 4.95E+02 | C24H50NO7P | LysoPC(16:1) | Lipids | LPC | - | A | 496.34, 184.08, 104.11, 478.33, 313.27 | 2.30E+07 | 8.89E+06 | 4.24E+06 | 2.74E+07 | 1.59E+07 | 1.44E+07 |
| Meta325 | 6.24E+02 | C28H32O16 | Isorhamnetin 3-O-neohesperidoside | Flavonoids | Flavonols | 55033-90-4 | A | 625.18, 317.07, 479.12, 129.06, 147.07 | 4.95E+07 | 1.12E+05 | 1.35E+07 | 3.04E+04 | 1.58E+07 | 4.41E+07 |
| Meta233 | 4.95E+02 | C24H50NO7P | LysoPC 16:0 | Lipids | LPC | 17364-16-8 | A | 496.34, 184.08, 104.11, 478.33, 313.27 | 2.18E+07 | 8.84E+06 | 4.12E+06 | 2.82E+07 | 1.57E+07 | 1.46E+07 |
| Meta47 | 2.28E+02 | C14H28O2 | Myristic Acid | Lipids | Free fatty acids | - | - | - | 1.40E+07 | 9.80E+06 | 2.76E+07 | 1.08E+07 | 1.56E+07 | 1.81E+07 |
| Meta150 | 2.19E+02 | C9H17NO5 | D-Pantothenic Acid | Others | Vitamin | 79-83-4 | A | 220.12, 90.06, 124.08, 72.04, 98.02 | 2.23E+07 | 1.18E+07 | 1.63E+07 | 1.12E+07 | 1.54E+07 | 1.23E+07 |
| Meta26 | 1.38E+02 | C7H8NO2+ | Trigonelline | Alkaloids | Alkaloids | - | - | - | 1.32E+07 | 2.00E+07 | 9.58E+06 | 1.52E+07 | 1.45E+07 | 1.40E+07 |
| Meta309 | 1.37E+02 | C8H11NO | L-Tyramine | Amino acids and derivatives | Amino acids and derivatives | 51-67-2 | A | 138.09, 121.07, 77.04, 103.06, 91.05 | 2.27E+07 | 1.16E+07 | 1.53E+07 | 6.90E+06 | 1.41E+07 | 1.23E+07 |
| Meta138 | 3.42E+02 | C12H22O11 | Galactinol | Others | Saccharides and Alcohols | 3687-64-7 | A | 341.11, 119.04, 89.02, 179.05, 59.01 | 2.46E+07 | 6.25E+06 | 1.88E+07 | 5.92E+06 | 1.39E+07 | 1.20E+07 |
| Meta39 | 6.10E+02 | C27H30O16 | Rutin | Flavonoids | Flavonols | 153-18-4 | A | 609.15, 609.14, 301.04, 300.03, 607.25 | 3.46E+07 | 9.55E+06 | 2.36E+06 | 8.07E+06 | 1.36E+07 | 1.23E+07 |
| Meta278 | 4.95E+02 | C24H50NO7P | LysoPC 16:0(2n isomer) | Lipids | LPC | - | A | 496.34, 184.08, 104.11, 478.33, 313.27 | 9.00E+00 | 4.36E+06 | 1.51E+06 | 4.85E+07 | 1.36E+07 | 1.11E+07 |
| Meta352 | 1.48E+02 | C6H12O4 | (Rs)-Mevalonic acid | Organic acids | Organic acids | 150-97-0 | B | 147.07, 59.01, 57.03, 61.99, 99.04 | 1.90E+07 | 4.34E+06 | 1.92E+07 | 8.87E+06 | 1.28E+07 | 1.19E+07 |
| Meta124 | 3.42E+02 | C16H22O8 | Coniferin | Phenolic acids | Phenolic acids | 531-29-3 | B | 341.12, 177.02, 179.08, 146.04, 179.07 | 3.89E+06 | 1.25E+07 | 1.32E+07 | 1.91E+07 | 1.22E+07 | 1.26E+07 |
| Meta368 | 3.16E+02 | C13H16O9 | Protocatechuic acid-4-glucoside | Phenolic acids | Phenolic acids | - | A | 315.07, 152.01, 108.02, 153.02, 109.03 | 4.13E+07 | 4.25E+06 | 2.49E+06 | 3.84E+05 | 1.21E+07 | 1.38E+07 |
| Meta256 | 2.96E+02 | C18H32O3 | 9,10-EODE | Lipids | Free fatty acids | 65167-83-1 | A | 295.23, 277.22, 195.14, 171.1, 183.1 | 1.62E+06 | 7.63E+05 | 4.46E+07 | 1.31E+06 | 1.21E+07 | 1.59E+07 |
| Meta103 | 1.45E+02 | C5H11N3O2 | 4-Guanidinobutyric acid | Organic acids | Organic acids | 463-00-3 | A | 146.09, 86.06, 87.04, 58.99, 90.48 | 1.64E+07 | 1.25E+07 | 5.41E+06 | 1.34E+07 | 1.19E+07 | 2.46E+07 |
| Meta374 | 3.42E+02 | C15H18O9 | 1-O-[(E)-Caffeoyl]-β-D-glucopyranose | Phenolic acids | Phenolic acids | - | A | 341.09, 179.04, 135.05, 341.09, 134.04 | 3.77E+06 | 1.27E+07 | 1.33E+07 | 1.76E+07 | 1.18E+07 | 1.22E+07 |
| Meta234 | 4.79E+02 | C23H46NO7P | LysoPE 18:1(2n isomer) | Lipids | LPE | - | A | 478.1, 281.3, 196.04, 140.01 | 8.61E+06 | 9.98E+06 | 7.59E+05 | 2.78E+07 | 1.18E+07 | 9.87E+06 |
| Meta189 | 1.80E+02 | C6H12O6 | D-Glucose | Others | Saccharides and Alcohols | 14431-43-7 | A | 179.06, 59.01, 71.01, 58.01, 85.03 | 1.35E+07 | 1.17E+07 | 1.15E+07 | 1.02E+07 | 1.17E+07 | 1.75E+07 |
| Meta341 | 1.65E+02 | C5H11NO3S | Methionine sulfoxide | Amino acids and derivatives | Amino acids and derivatives | - | - | - | 1.12E+07 | 1.16E+07 | 1.13E+07 | 1.20E+07 | 1.15E+07 | 1.13E+07 |
| Meta317 | 1.49E+02 | C5H11NO2S | L-Methionine | Amino acids and derivatives | Amino acids and derivatives | 63-68-3 | A | 150.06, 56.05, 61.01, 104.05, 122.03 | 2.13E+07 | 6.39E+06 | 1.34E+07 | 4.89E+06 | 1.15E+07 | 1.81E+07 |
| Meta20 | 2.96E+02 | C18H32O3 | 9-Hydroxy-10,12-octadecadienoic acid | Lipids | Free fatty acids | 15514-85-9 | B | 295.23, 277.22, 195.14, 171.1, 183.1 | 7.97E+05 | 4.34E+05 | 4.25E+07 | 9.28E+05 | 1.12E+07 | 1.32E+07 |
| Meta19 | 2.96E+02 | C18H32O3 | 13-Hydroxy-9,11-octadecadienoic acid | Lipids | Free fatty acids | 5204-88-6 | B | 295.23, 277.22, 195.14, 171.1, 183.1 | 7.92E+05 | 4.28E+05 | 4.20E+07 | 9.13E+05 | 1.10E+07 | 1.31E+07 |
| Meta392 | 2.96E+02 | C18H32O3 | 9S-Hyroxy-10E,12E-octadecadienoic acid | Lipids | Free fatty acids | - | B | 295.23, 277.22, 195.14, 171.1, 183.1 | 7.91E+05 | 4.15E+05 | 4.14E+07 | 9.04E+05 | 1.09E+07 | 1.30E+07 |
| Meta84 | 4.79E+02 | C23H46NO7P | LysoPE 18:1 | Lipids | LPE | 89576-29-4 | A | 478.1, 281.3, 196.04, 140.01 | 7.90E+06 | 8.38E+06 | 6.21E+05 | 2.32E+07 | 1.00E+07 | 8.33E+06 |
| Meta261 | 3.16E+02 | C13H16O9 | 2,5-Dihydroxy benzoic acid O-hexside | Phenolic acids | Phenolic acids | - | A | 315.07, 152.01, 108.02, 153.02, 109.03 | 3.44E+07 | 3.42E+06 | 1.63E+06 | 5.98E+05 | 1.00E+07 | 1.13E+07 |
| Meta40 | 4.64E+02 | C21H20O12 | Hyperin | Flavonoids | Flavonols | 482-36-0 | A | 463.09, 300.03, 301.04, 271.03, 255.03 | 3.15E+07 | 4.09E+05 | 6.83E+06 | 1.55E+05 | 9.73E+06 | 8.53E+06 |
| Meta350 | 1.03E+02 | C4H9NO2 | 2-Aminoisobutyric acid | Amino acids and derivatives | Amino acids and derivatives | - | - | 104.07, 69.04, 87.05, 68.05, 86.07 | 1.26E+07 | 6.59E+06 | 1.33E+07 | 5.51E+06 | 9.51E+06 | 1.07E+07 |
| Meta396 | 3.72E+02 | C16H20O10 | Trihydroxycinnamoylquinic acid | Phenolic acids | Phenolic acids | - | A | 371.1, 249.06, 121.03, 113.02, 231.05 | 2.50E+07 | 3.69E+06 | 5.85E+06 | 2.66E+06 | 9.30E+06 | 1.29E+07 |
| Meta430 | 1.48E+02 | C8H4O3 | Phthalic anhydride | Phenolic acids | Phenolic acids | 85-44-9 | A | 149.02, 65.04, 121.04, 93.04, 80.03 | 1.72E+07 | 6.68E+06 | 2.98E+06 | 7.82E+06 | 8.66E+06 | 1.22E+07 |
| Meta119 | 1.66E+02 | C8H15NaO2 | Sodium Valproate | Organic acids | Organic acids | 1069-66-5 | A | 143.11, 143.11, 142.68, 97.8, | 1.56E+07 | 2.86E+06 | 1.15E+07 | 4.49E+06 | 8.61E+06 | 1.08E+07 |
| Meta307 | 3.42E+02 | C12H22O11 | D-(+)-Sucrose | Others | Saccharides and Alcohols | 57-50-1 | A | 341.11, 119.04, 89.02, 179.05, 59.01 | 1.56E+07 | 3.44E+06 | 1.17E+07 | 2.93E+06 | 8.40E+06 | 5.90E+06 |
| Meta43 | 4.64E+02 | C21H20O12 | Isoquercitrin | Flavonoids | Flavonols | 482-35-9 | A | 463.09, 300.03, 301.04, 271.03, 255.03 | 2.73E+07 | 3.74E+05 | 5.08E+06 | 1.23E+05 | 8.23E+06 | 7.16E+06 |
| Meta367 | 4.16E+02 | C18H24O11 | Regaloside L | Phenolic acids | Phenolic acids | - | A | 415.13, 415.12, 161.02, 179.03, 235.06 | 1.47E+07 | 4.47E+05 | 1.72E+07 | 2.03E+05 | 8.14E+06 | 6.41E+06 |
| Meta77 | 1.50E+02 | C4H6O6 | L-(+)-Tartaric acid | Organic acids | Organic acids | 87-69-4 | A | 149.01, 72.99, 87.01, 59.01, 103 | 1.09E+07 | 3.39E+06 | 1.22E+07 | 5.37E+06 | 7.96E+06 | 8.32E+06 |
| Meta63 | 1.80E+02 | C6H12O6 | D-(+)-Glucose | Others | Saccharides and Alcohols | 50-99-7 | A | 179.06, 59.01, 71.01, 58.01, 85.03 | 8.78E+06 | 7.17E+06 | 8.61E+06 | 5.52E+06 | 7.52E+06 | 1.06E+07 |
| Meta294 | 2.67E+02 | C10H13N5O4 | Adenosine | Nucleotides and derivatives | Nucleotides and derivatives | 58-61-7 | A | 268.1, 136.06, 119.04, 135.65, 57.03 | 1.13E+06 | 1.46E+05 | 2.70E+07 | 1.59E+05 | 7.12E+06 | 6.51E+06 |
| Meta300 | 5.94E+02 | C27H30O15 | Kaempferol 3-O-rutinoside(Nicotiflorin) | Flavonoids | Flavonols | 17650-84-9 | A | 595.17, 457.11, 577.15, 595.17, 559.15 | 1.36E+07 | 3.75E+06 | 7.62E+06 | 3.20E+06 | 7.05E+06 | 4.69E+06 |
| Meta25 | 1.29E+02 | C6H11NO2 | Pipecolic acid | Amino acids and derivatives | Amino acids and derivatives | 4043-87-2 | B | 130.09, 84.08, 55 | 6.89E+06 | 5.81E+06 | 6.10E+06 | 8.78E+06 | 6.89E+06 | 7.65E+06 |
| Meta286 | 1.35E+02 | C5H5N5 | Adenine | Nucleotides and derivatives | Nucleotides and derivatives | 73-24-5 | A | 136.06, 119.04, 136.06, 92.03, 94.04 | 1.12E+06 | 1.98E+06 | 2.17E+07 | 1.09E+06 | 6.46E+06 | 5.48E+06 |
| Meta355 | 4.64E+02 | C21H20O12 | Quercetin 3-O-glucoside(Isotrifoliin) | Flavonoids | Flavonols | 21637-25-2 | A | 463.09, 300.03, 301.04, 271.03, 255.03 | 2.05E+07 | 2.62E+05 | 3.87E+06 | 9.30E+04 | 6.19E+06 | 5.22E+06 |
| Meta277 | 4.67E+02 | C22H46NO7P | LysoPC 14:0(2n isomer) | Lipids | LPC | - | A | 468.31, 184.07, 104.11, 468.3, 450.3 | 1.37E+05 | 3.70E+06 | 9.27E+04 | 2.08E+07 | 6.18E+06 | 3.54E+06 |
| Meta251 | 3.52E+02 | C21H36O4 | MAG(18:3)isomer1 | Lipids | Glycerol ester | - | B | 353.3, 261.1, 243.21, 121.1 | 8.56E+05 | 3.13E+05 | 2.08E+07 | 2.52E+06 | 6.13E+06 | 6.78E+06 |
| Meta100 | 1.21E+02 | C8H11N | Phenethylamine | Others | Others | 64-04-0 | A | 122.1, 105.07, 77.04, 79.06, 103.06 | 9.41E+06 | 2.83E+06 | 4.74E+06 | 6.62E+06 | 5.90E+06 | 5.13E+06 |
| Meta120 | 4.64E+02 | C21H20O12 | Spiraeoside | Flavonoids | Flavonols | 20229-56-5 | A | 463.09, 300.03, 301.04, 271.03, 255.03 | 1.96E+07 | 2.66E+05 | 3.62E+06 | 8.00E+04 | 5.89E+06 | 5.08E+06 |
| Meta361 | 3.11E+02 | C12H17N5O5 | 2-(Dimethylamino)guanosine | Nucleotides and derivatives | Nucleotides and derivatives | 2140-67-2 | A | 312.13, 180.09, 135.03, 110.03, 137.08 | 3.68E+05 | 8.97E+06 | 7.41E+06 | 6.45E+06 | 5.80E+06 | 2.08E+06 |
| Meta250 | 3.56E+02 | C21H40O4 | MAG(18:1)isomer1 | Lipids | Glycerol ester | - | A | 357.3, 265.25, 339.29, 135.12, 247.24 | 3.20E+06 | 3.52E+06 | 7.59E+05 | 1.56E+07 | 5.76E+06 | 5.02E+06 |
| Meta326 | 5.94E+02 | C27H30O15 | Kaempferol 3-O-robinobioside(Biorobin) | Flavonoids | Flavonols | 17297-56-2 | A | 595.17, 457.11, 577.15, 595.17, 559.15 | 1.15E+07 | 2.78E+06 | 6.54E+06 | 2.26E+06 | 5.76E+06 | 4.32E+06 |
| Meta204 | 3.56E+02 | C21H40O4 | MAG(18:1)isomer2 | Lipids | Glycerol ester | - | A | 357.3, 265.25, 339.29, 135.12, 247.24 | 3.10E+06 | 3.31E+06 | 7.49E+05 | 1.49E+07 | 5.51E+06 | 4.89E+06 |
| Meta419 | 5.20E+02 | C26H51NO7P+ | PC(18:2) | Lipids | PC | - | B | 520.34, 184.07, 520.34, 502.33, 125 | 1.21E+06 | 2.69E+06 | 1.14E+06 | 1.69E+07 | 5.48E+06 | 4.45E+06 |
| Meta186 | 1.53E+02 | C8H11NO2 | Dopamine hydrochloride | Alkaloids | Alkaloids | 62-31-7 | B | 154.09, 91.06, 137.06, 119.05, 65.04 | 6.11E+06 | 1.20E+06 | 2.83E+06 | 1.16E+07 | 5.44E+06 | 3.56E+06 |
| Meta420 | 5.20E+02 | C26H51NO7P+ | PC(18:2)isomer | Lipids | PC | - | A | 520.34, 184.07, 520.34, 502.33, 125 | 1.23E+06 | 2.70E+06 | 1.11E+06 | 1.65E+07 | 5.39E+06 | 4.39E+06 |
| Meta427 | 5.21E+02 | C26H52NO7P | LysoPC(18:1) | Lipids | LPC | - | A | 522.36, 522.36, 184.07, 104.11, 504.35 | 2.65E+06 | 3.02E+06 | 6.56E+05 | 1.51E+07 | 5.35E+06 | 4.14E+06 |
| Meta373 | 3.26E+02 | C15H18O8 | 1-O-[(E)-p-Cumaroyl]-β-D-glucopyranose | Phenolic acids | Phenolic acids | - | B | 325.1, 119.05, 163.04, 151.04, 118.69 | 3.17E+06 | 9.00E+00 | 1.78E+07 | 9.00E+00 | 5.25E+06 | 4.35E+06 |
| Meta425 | 5.19E+02 | C26H50NO7P | LysoPC(18:2) | Lipids | LPC | - | A | 520.34, 184.07, 104.11, 502.33, 86.1 | 1.24E+06 | 2.65E+06 | 1.12E+06 | 1.59E+07 | 5.23E+06 | 4.43E+06 |
| Meta195 | 3.42E+02 | C12H22O11 | Isomaltulose | Others | Saccharides and Alcohols | 13718-94-0 | B | 341.11, 119.04, 89.02, 179.05, 59.01 | 9.57E+06 | 2.52E+06 | 6.93E+06 | 1.83E+06 | 5.21E+06 | 4.03E+06 |
| Meta241 | 3.54E+02 | C21H38O4 | MAG(18:2) | Lipids | Glycerol ester | - | A | 355.29, 263.24, 245.23, 337.27, 95.09 | 1.16E+06 | 1.55E+06 | 1.20E+07 | 5.24E+06 | 5.00E+06 | 5.41E+06 |
| Meta281 | 1.15E+02 | C5H9NO2 | L-Proline | Amino acids and derivatives | Amino acids and derivatives | 147-85-3 | A | 116.07, 70.07, 68.05, 116.07, 69.86 | 5.98E+05 | 1.38E+07 | 2.80E+05 | 3.50E+06 | 4.54E+06 | 5.21E+06 |
| Meta146 | 1.94E+02 | C6H10O7 | Aldehydo-D-galacturonate | Organic acids | Organic acids | 685-73-4 | A | 193.03, 59.01, 71.01, 72.99, 85.03 | 1.06E+07 | 3.20E+05 | 6.46E+06 | 7.07E+05 | 4.53E+06 | 4.20E+06 |
| Meta28 | 1.94E+02 | C10H10O4 | Ferulic acid | Phenolic acids | Phenolic acids | 1135-24-6 | A | 193.05, 134.04, 178.03, 133.03, 137.03 | 2.82E+05 | 4.74E+06 | 9.59E+06 | 3.10E+06 | 4.43E+06 | 2.81E+06 |
| Meta280 | 4.53E+02 | C21H44NO7P | LysoPE 16:0(2n isomer) | Lipids | LPE | - | A | 454.29, 313.28, 436.28, 282.28, 393.24 | 6.37E+06 | 1.94E+06 | 7.81E+05 | 8.44E+06 | 4.38E+06 | 3.85E+06 |
| Meta246 | 3.52E+02 | C21H36O4 | MAG(18:3)isomer3 | Lipids | Glycerol ester | - | A | 353.3, 261.1, 243.21, 121.1 | 1.01E+07 | 2.48E+06 | 6.36E+05 | 3.48E+06 | 4.18E+06 | 4.87E+06 |
| Meta366 | 1.15E+02 | C5H9NO2 | DL-Proline | Amino acids and derivatives | Amino acids and derivatives | 609-36-9 | A | 116.07, 70.07, 68.05, 116.07, 69.86 | 5.65E+05 | 1.26E+07 | 2.59E+05 | 3.10E+06 | 4.13E+06 | 4.74E+06 |
| Meta358 | 1.94E+02 | C6H10O7 | D-Glucoronic acid | Others | Saccharides and Alcohols | 6556-12-3 | A | 193.03, 59.01, 71.01, 72.99, 85.03 | 9.56E+06 | 3.38E+05 | 5.79E+06 | 7.31E+05 | 4.11E+06 | 3.57E+06 |
| Meta239 | 4.53E+02 | C21H44NO7P | LysoPE 16:0 | Lipids | LPE | 53862-35-4 | A | 454.29, 313.28, 436.28, 282.28, 393.24 | 5.83E+06 | 1.81E+06 | 7.53E+05 | 7.91E+06 | 4.07E+06 | 3.63E+06 |
| Meta15 | 6.24E+02 | C28H32O16 | Rhamnetin-O-glucoside-O-rhamnoside | Flavonoids | Flavonoid | - | B | 625.18, 317.07, 479.12, 129.06, 147.07 | 1.06E+07 | 1.07E+05 | 5.08E+06 | 4.30E+04 | 3.96E+06 | 5.43E+06 |
| Meta276 | 3.30E+02 | C17H14O7 | Di-O-methylquercetin | Flavonoids | Flavonols | 2068-02-2 | B | 331.08, 316.06, 301.03, 168, 273.04 | 5.24E+05 | 1.77E+06 | 1.20E+07 | 1.12E+06 | 3.86E+06 | 3.31E+06 |
| Meta296 | 1.31E+02 | C6H13NO2 | 6-Aminocaproic acid | Organic acids | Organic acids | 60-32-2 | B | 132.1, 86.1, 69.07, 55.05, 86.11 | 5.01E+06 | 1.22E+06 | 5.36E+06 | 3.72E+06 | 3.83E+06 | 3.74E+06 |
| Meta417 | 1.31E+02 | C6H13NO2 | 6-Deoxyfagomine | Alkaloids | Alkaloids | 197449-09-5 | B | 132.1, 86.1, 69.07, 55.05, 86.11 | 4.65E+06 | 1.51E+06 | 4.91E+06 | 4.13E+06 | 3.80E+06 | 4.33E+06 |
| Meta271 | 3.38E+02 | C16H18O8 | 3-O-p-Coumaroyl quinic acid | Phenolic acids | Phenolic acids | 1899-30-5 | A | 337.09, 163.04, 191.06, 119.05, 173.05 | 2.55E+06 | 3.49E+05 | 7.11E+06 | 4.55E+06 | 3.64E+06 | 2.75E+06 |
| Meta74 | 1.81E+02 | C9H11NO3 | L-(-)-Tyrosine | Amino acids and derivatives | Amino acids and derivatives | 60-18-4 | A | 182.08, 119.05, 146.01, 136.08, 91.05 | 1.94E+06 | 7.26E+06 | 2.05E+06 | 3.27E+06 | 3.63E+06 | 2.83E+06 |
| Meta407 | 5.94E+02 | C27H30O15 | Luteolin-7-O-rutinoside | Flavonoids | Flavonoid | 3563-98-2 | A | 595.17, 457.11, 577.15, 595.17, 559.15 | 6.78E+06 | 3.10E+06 | 2.76E+06 | 1.73E+06 | 3.59E+06 | 2.54E+06 |
| Meta153 | 1.94E+02 | C10H10O4 | Trans-ferulic acid | Phenolic acids | Phenolic acids | 537-98-4 | A | 193.05, 134.04, 178.03, 133.03, 137.03 | 2.30E+05 | 3.69E+06 | 7.80E+06 | 2.44E+06 | 3.54E+06 | 2.52E+06 |
| Meta143 | 1.94E+02 | C6H10O7 | D-Galacturonic acid(Gal A) | Organic acids | Organic acids | 91510-62-2 | B | 193.03, 59.01, 71.01, 72.99, 85.03 | 8.14E+06 | 2.69E+05 | 4.77E+06 | 6.66E+05 | 3.46E+06 | 3.46E+06 |
| Meta79 | 3.42E+02 | C12H22O11 | D-(+)-TrehaloseAnhydrous | Others | Saccharides and Alcohols | 99-20-7 | A | 341.11, 119.04, 89.02, 179.05, 59.01 | 6.34E+06 | 1.36E+06 | 4.76E+06 | 1.36E+06 | 3.46E+06 | 2.30E+06 |
| Meta06 | 6.37E+02 | C27H29N2O16 | Phyllocactin II | Alkaloids | Alkaloids | - | A | 637.14, 389.1 | 3.68E+05 | 1.66E+03 | 1.56E+06 | 1.19E+07 | 3.45E+06 | 1.60E+06 |
| Meta395 | 3.30E+02 | C18H34O5 | 9,10,13-Trihyroxy-11-octadecadienoic acid | Lipids | Free fatty acids | - | B | 329.24, 329.23, 211.14, 171.1, 229.15 | 4.66E+05 | 1.54E+06 | 1.05E+07 | 9.89E+05 | 3.37E+06 | 2.89E+06 |
| Meta337 | 4.48E+02 | C21H20O11 | Luteolin 7-O-glucoside(Cynaroside) | Flavonoids | Flavonoid | 5373-11-5 | A | 447.09, 285.04, 284.03, 284.05, 327.05 | 4.28E+06 | 9.00E+00 | 9.08E+06 | 9.00E+00 | 3.34E+06 | 2.85E+06 |
| Meta321 | 1.69E+02 | C8H11NO3 | Pyridoxine | Others | Vitamin | 65-23-6 | A | 170.08, 134.06, 152.07, 124.08, 79.05 | 1.54E+06 | 7.32E+06 | 3.40E+06 | 1.11E+06 | 3.34E+06 | 4.60E+06 |
| Meta07 | 6.37E+02 | C27H29N2O16 | isophyllocactin | Alkaloids | Alkaloids | - | A | 637.14, 389.1 | 3.73E+05 | 2.15E+03 | 1.47E+06 | 1.14E+07 | 3.32E+06 | 1.51E+06 |
| Meta424 | 4.93E+02 | C24H48NO7P | LysoPC(16:2) | Lipids | LPC | - | A | 494.32, 184.07, 104.11, 476.31, 476.3 | 3.07E+05 | 1.08E+06 | 2.98E+05 | 1.15E+07 | 3.29E+06 | 1.82E+06 |
| Meta359 | 2.43E+02 | C9H13N3O5 | Cytidine | Nucleotides and derivatives | Nucleotides and derivatives | 65-46-3 | B | 244.09, 112.05, 95.02, 69.05, 94.04 | 7.09E+05 | 2.04E+06 | 8.11E+06 | 1.63E+06 | 3.12E+06 | 2.62E+06 |
| Meta128 | 3.25E+02 | C20H39NO2 | N-Oleoylethanolamine | Others | Others | 111-58-0 | A | 326.31, 62.06, 309.28, 62.07, 308.29 | 7.22E+05 | 7.73E+05 | 9.98E+06 | 4.65E+05 | 2.99E+06 | 1.98E+06 |
| Meta81 | 1.34E+02 | C4H6O5 | L-(-)-Malic acid | Organic acids | Organic acids | 636-61-3 | A | 133.01, 71.01, 115, 72.99, 133.01 | 1.98E+06 | 3.25E+06 | 1.24E+06 | 5.44E+06 | 2.98E+06 | 3.69E+06 |
| Meta149 | 3.42E+02 | C12H22O11 | Melibiose | Others | Saccharides and Alcohols | 585-99-9 | A | 341.11, 119.04, 89.02, 179.05, 59.01 | 5.49E+06 | 1.34E+06 | 3.76E+06 | 1.11E+06 | 2.93E+06 | 2.08E+06 |
| Meta141 | 7.86E+02 | C35H46O20 | Echinacoside | Phenolic acids | Phenolic acids | 82854-37-3 | B | 785.26, 623.23, 161.03, 179.04, 782.82 | 9.00E+00 | 1.15E+04 | 1.15E+07 | 2.09E+04 | 2.88E+06 | 2.05E+06 |
| Meta273 | 2.90E+02 | C6H11PO11 | Glucarate O-Phosphoric acid | Others | Saccharides and Alcohols | - | B | 289.1, 97, 78.96, 143 | 9.05E+05 | 7.73E+06 | 2.56E+05 | 2.41E+06 | 2.83E+06 | 1.87E+06 |
| Meta311 | 1.51E+02 | C5H5N5O | Guanine | Nucleotides and derivatives | Nucleotides and derivatives | 73-40-5 | A | 152.06, 135.03, 110.04, 55.03, 82.04 | 2.70E+05 | 1.76E+05 | 1.07E+07 | 1.17E+05 | 2.81E+06 | 2.47E+06 |
| Meta172 | 1.65E+02 | C9H11NO2 | D-(+)-Phenylalanine | Amino acids and derivatives | Amino acids and derivatives | - | - | - | 2.44E+06 | 2.21E+06 | 4.35E+06 | 2.08E+06 | 2.77E+06 | 2.83E+06 |
| Meta98 | 1.18E+02 | C4H6O4 | Methylmalonic acid | Organic acids | Organic acids | 516-05-2 | A | 117.02, 73.03, 99.01, 55.02, 117.02 | 5.84E+06 | 9.64E+05 | 2.86E+06 | 1.30E+06 | 2.74E+06 | 4.94E+06 |
| Meta202 | 3.52E+02 | C21H36O4 | MAG(18:3)isomer5 | Lipids | Glycerol ester | - | A | 353.3, 261.1, 243.21, 121.1 | 3.52E+06 | 1.52E+06 | 1.32E+06 | 4.61E+06 | 2.74E+06 | 2.30E+06 |
| Meta262 | 3.86E+02 | C17H22O10 | 1-O-β-D-Glucopyranosyl sinapate | Phenolic acids | Phenolic acids | 78185-48-5 | B | 385.1, 205, 190.03, 59.01 | 3.17E+06 | 1.31E+06 | 1.08E+06 | 5.38E+06 | 2.74E+06 | 1.65E+06 |
| Meta360 | 2.51E+02 | C10H13N5O3 | Deoxyadenosine | Nucleotides and derivatives | Nucleotides and derivatives | 958-09-8 | A | 252.11, 136.06, 119.04, 117.05, 99.04 | 1.66E+05 | 2.23E+05 | 1.03E+07 | 2.56E+05 | 2.73E+06 | 2.29E+06 |
| Meta62 | 1.18E+02 | C4H6O4 | Succinic acid | Organic acids | Organic acids | 110-15-6 | A | 117.02, 73.03, 99.01, 55.02, 117.02 | 5.59E+06 | 9.49E+05 | 2.76E+06 | 1.24E+06 | 2.63E+06 | 4.82E+06 |
| Meta71 | 1.88E+02 | C9H16O4 | Anchoic Acid | Organic acids | Organic acids | 123-99-9 | B | 187.1, 125.1, 187.1, 97.06, 123.08 | 6.69E+05 | 3.50E+05 | 8.87E+06 | 6.08E+05 | 2.62E+06 | 1.67E+06 |
| Meta376 | 3.86E+02 | C19H30O8 | Roseoside | Others | Others | - | B | 385.19, 153.09, 205.12, 152.08, 59.01 | 6.30E+06 | 2.50E+05 | 2.72E+06 | 1.06E+06 | 2.58E+06 | 2.37E+06 |
| Meta66 | 1.33E+02 | C4H7NO4 | L-AsparticAcid | Amino acids and derivatives | Amino acids and derivatives | 56-84-8 | A | 132.03, 88.04, 71.01, 132.03, 115 | 2.23E+06 | 3.61E+06 | 2.79E+06 | 1.56E+06 | 2.54E+06 | 3.13E+06 |
| Meta390 | 1.80E+02 | C9H8O4 | Sorbic acid | Phenolic acids | Phenolic acids | 110-44-1 | A | 179.03, 135.05, 134.04, 107.05, 117.04 | 1.69E+05 | 9.00E+00 | 9.71E+06 | 1.41E+05 | 2.51E+06 | 1.59E+06 |
| Meta122 | 2.60E+02 | C6H13O9P | D-Glucose 6-phosphate | Others | Saccharides and Alcohols | 56-73-5 | B | 259.02, 96.97, 78.96, 198.96, 138.94 | 2.67E+06 | 3.41E+06 | 4.03E+05 | 3.36E+06 | 2.46E+06 | 2.20E+06 |
| Meta139 | 2.60E+02 | C6H13O9P | Glucose-1-phosphate | Others | Saccharides and Alcohols | 59-56-3 | A | 259.02, 96.97, 78.96, 198.96, 138.94 | 2.62E+06 | 3.30E+06 | 4.16E+05 | 3.39E+06 | 2.43E+06 | 2.26E+06 |
| Meta94 | 1.30E+02 | C5H6O4 | Citraconic acid | Organic acids | Organic acids | 498-23-7 | A | 129.02, 121.93, 123.04, 123, 124.91 | 6.08E+06 | 1.21E+05 | 3.22E+06 | 1.95E+05 | 2.40E+06 | 2.21E+06 |
| Meta380 | 2.56E+02 | C16H32O2 | Hexadecanoic acid | Phenolic acids | Phenolic acids | - | - | - | 1.85E+06 | 2.42E+06 | 2.93E+06 | 2.34E+06 | 2.39E+06 | 2.49E+06 |
| Meta02 | 6.24E+02 | C28H32O16 | Isorhamnetin-3-O-rutinoside | Flavonoids | Flavonols | 604-80-8 | B | 625.18, 317.07, 479.12, 129.06, 147.07 | 6.28E+06 | 4.45E+04 | 3.01E+06 | 1.89E+04 | 2.34E+06 | 2.65E+06 |
| Meta111 | 2.84E+02 | C10H12N4O6 | Xanthosine | Nucleotides and derivatives | Nucleotides and derivatives | 146-80-5 | A | 283.07, 151.03, 283.07, 108.02, 222.99 | 1.55E+05 | 5.07E+06 | 1.07E+04 | 3.32E+06 | 2.14E+06 | 2.27E+06 |
| Meta297 | 1.66E+02 | C8H6O4 | Terephthalic acid | Phenolic acids | Phenolic acids | - | - | - | 3.03E+06 | 6.23E+05 | 2.44E+06 | 1.10E+06 | 1.80E+06 | 2.09E+06 |
| Meta304 | 1.23E+02 | C6H5NO2 | Nicotinic acid | Others | Vitamin | 59-67-6 | A | 124.04, 78.03, 124.04, 80.05, 53.04 | 4.74E+05 | 1.31E+06 | 4.53E+06 | 5.23E+05 | 1.71E+06 | 1.37E+06 |
| Meta284 | 1.46E+02 | C6H14N2O2 | L-(+)-Lysine | Amino acids and derivatives | Amino acids and derivatives | 56-87-1 | B | 147.11, 84.08, 56.05, 67.06, 130.09 | 2.20E+06 | 1.15E+06 | 2.44E+06 | 9.03E+05 | 1.67E+06 | 1.42E+06 |
| Meta279 | 5.23E+02 | C26H54NO7P | LysoPC 18:0 | Lipids | LPC | - | A | 524.37, 184.08, 104.11, 506.36, 184.1 | 6.52E+04 | 2.65E+05 | 6.77E+04 | 6.29E+06 | 1.67E+06 | 1.00E+06 |
| Meta421 | 3.13E+02 | C18H19NO4 | N-Cis-feruloyltyramine | Alkaloids | Phenolamine | 80510-09-4 | A | 314.1, 145, 177.06, 121.06 | 1.34E+06 | 2.84E+05 | 4.18E+06 | 7.50E+05 | 1.64E+06 | 1.44E+06 |
| Meta282 | 1.47E+02 | C5H9NO4 | L-Glutamic acid | Amino acids and derivatives | Amino acids and derivatives | 56-86-0 | B | 148.06, 60.05, 88.04, 70.03, 106.05 | 2.53E+06 | 1.23E+06 | 1.98E+05 | 2.53E+06 | 1.62E+06 | 6.25E+05 |
| Meta293 | 1.46E+02 | C5H10N2O3 | L-Glutamine | Amino acids and derivatives | Amino acids and derivatives | 56-85-9 | A | 147.08, 84.05, 56.05, 130.05, 102.06 | 1.98E+06 | 1.07E+06 | 2.51E+06 | 8.00E+05 | 1.59E+06 | 1.23E+06 |
| Meta170 | 1.47E+02 | C5H9NO4 | Glutamic acid | Amino acids and derivatives | Amino acids and derivatives | 617-65-2 | B | 148.06, 60.05, 88.04, 70.03, 106.05 | 2.15E+06 | 1.36E+06 | 2.41E+05 | 2.53E+06 | 1.57E+06 | 6.47E+05 |
| Meta252 | 2.46E+02 | C13H14N2O3 | Acetyltryptophan | Amino acids and derivatives | Amino acids and derivatives | 2280-01-5 | A | 245.09, 203.08, 74.02, 116.05, 201.11 | 6.10E+04 | 7.74E+04 | 4.97E+06 | 1.02E+06 | 1.53E+06 | 1.09E+06 |
| Meta09 | 7.26E+02 | C30H34N2O19 | amaranthin | Alkaloids | Alkaloids | - | A | 727.19, 389.09 | 2.04E+04 | 9.00E+00 | 5.76E+06 | 8.13E+04 | 1.46E+06 | 7.58E+05 |
| Meta283 | 1.65E+02 | C9H11NO2 | L-Phenylalanine | Amino acids and derivatives | Amino acids and derivatives | - | - | - | 1.30E+06 | 1.13E+06 | 2.23E+06 | 1.08E+06 | 1.44E+06 | 1.46E+06 |
| Meta76 | 1.31E+02 | C6H13NO2 | L-Isoleucine | Amino acids and derivatives | Amino acids and derivatives | 73-32-5 | A | 132.1, 86.1, 69.07, 55.05, 86.11 | 1.61E+06 | 4.99E+05 | 2.08E+06 | 1.46E+06 | 1.41E+06 | 1.60E+06 |
| Meta68 | 1.31E+02 | C6H13NO2 | L-Leucine | Amino acids and derivatives | Amino acids and derivatives | 61-90-5 | A | 132.1, 86.1, 69.07, 55.05, 86.11 | 1.62E+06 | 4.95E+05 | 1.99E+06 | 1.45E+06 | 1.39E+06 | 1.60E+06 |
| Meta165 | 1.31E+02 | C6H13NO2 | α-Aminocaproic acid | Amino acids and derivatives | Amino acids and derivatives | 327-57-1 | A | 132.1, 86.1, 69.07, 55.05, 86.11 | 1.57E+06 | 4.78E+05 | 1.95E+06 | 1.35E+06 | 1.34E+06 | 1.52E+06 |
| Meta351 | 1.03E+02 | C4H9NO2 | N,N-Dimethylglycine | Amino acids and derivatives | Amino acids and derivatives | 1118-68-9 | A | 104.07, 69.04, 87.05, 68.05, 86.07 | 1.75E+06 | 4.59E+05 | 2.63E+06 | 4.50E+05 | 1.32E+06 | 1.82E+06 |
| Meta99 | 1.32E+02 | C5H8O4 | 2-Methylsuccinic acid | Organic acids | Organic acids | - | - | - | 1.79E+06 | 9.42E+05 | 2.49E+06 | 9.00E+00 | 1.30E+06 | 2.06E+06 |
| Meta115 | 1.86E+02 | C11H22O2 | Undecylic Acid | Lipids | Free fatty acids | - | - | - | 1.63E+06 | 6.08E+05 | 2.35E+06 | 5.89E+05 | 1.30E+06 | 1.42E+06 |
| Meta51 | 1.22E+02 | C6H6N2O | Nicotinamide | Others | Vitamin | - | - | - | 2.27E+06 | 1.91E+05 | 2.42E+06 | 2.16E+05 | 1.27E+06 | 1.28E+06 |
| Meta12 | 6.10E+02 | C27H30O16 | Quercetin 3-O-rhanosylgalactoside | Flavonoids | Flavonols | - | A | 609.15, 609.14, 301.04, 300.03, 607.25 | 3.33E+06 | 7.51E+05 | 1.90E+05 | 7.66E+05 | 1.26E+06 | 8.56E+05 |
| Meta225 | 6.10E+02 | C27H30O16 | Luteolin 8-C-hexosyl-O-hexoside | Flavonoids | Flavonoid | - | B | 609.15, 609.14, 301.04, 300.03, 607.25 | 3.08E+06 | 8.67E+05 | 1.82E+05 | 8.13E+05 | 1.23E+06 | 8.95E+05 |
| Meta04 | 6.10E+02 | C27H30O16 | Quercetin-3-O-glucoside-7-O-rhamnoside | Flavonoids | Flavonols | - | A | 609.15, 609.14, 301.04, 300.03, 607.25 | 3.08E+06 | 8.79E+05 | 1.81E+05 | 7.41E+05 | 1.22E+06 | 9.05E+05 |
| Meta331 | 1.50E+02 | C5H10O5 | DL-Arabinose | Others | Saccharides and Alcohols | 147-81-9 | B | 149, 59, 72.99, 87.01 | 2.14E+06 | 2.21E+05 | 2.37E+06 | 1.44E+05 | 1.22E+06 | 1.43E+06 |
| Meta375 | 3.38E+02 | C16H18O8 | 3-O-(E)-p-Coumaroyl quinic acid | Phenolic acids | Phenolic acids | 87099-71-6 | A | 337.09, 163.04, 191.06, 119.05, 173.05 | 3.80E+06 | 8.53E+05 | 9.00E+00 | 9.00E+00 | 1.16E+06 | 8.14E+05 |
| Meta175 | 3.54E+02 | C16H18O9 | Cryptochlorogenic acid | Phenolic acids | Phenolic acids | - | - | 353.09, 191.06, 161.03, 179.04, 173.05 | 9.60E+05 | 1.80E+06 | 1.61E+06 | 2.73E+05 | 1.16E+06 | 3.60E+06 |
| Meta349 | 1.03E+02 | C4H9NO2 | γ-Aminobutyric acid | Organic acids | Organic acids | 56-12-2 | B | 104.07, 69.04, 87.05, 68.05, 86.07 | 1.29E+06 | 6.35E+04 | 3.06E+06 | 1.02E+05 | 1.13E+06 | 2.05E+06 |
| Meta308 | 1.96E+02 | C6H12O7 | Gluconic acid | Others | Saccharides and Alcohols | 526-95-4 | A | 195.05, 75.01, 59.01, 195.05, 129.02 | 1.53E+06 | 1.52E+06 | 9.11E+05 | 5.05E+05 | 1.12E+06 | 9.60E+05 |
| Meta70 | 3.76E+02 | C17H20N4O6 | Riboflavin | Others | Vitamin | 83-88-5 | A | 377.15, 243.09, 359.14, 172.09, 198.07 | 1.22E+05 | 1.48E+06 | 2.07E+06 | 6.96E+05 | 1.09E+06 | 8.19E+05 |
| Meta193 | 2.78E+02 | C15H22N2O3 | Leucylphenylalanine | Amino acids and derivatives | Amino acids and derivatives | 56217-82-4 | B | 279.17, 120.08, 166.09, 86.1, 86.11 | 6.23E+05 | 7.60E+04 | 3.59E+06 | 5.65E+04 | 1.09E+06 | 6.86E+05 |
| Meta330 | 3.54E+02 | C16H18O9 | Neochlorogenic acid(5-O-Caffeoylquinic acid) | Phenolic acids | Phenolic acids | 906-33-2 | A | 353.09, 191.06, 161.03, 179.04, 173.05 | 3.15E+06 | 5.69E+05 | 5.21E+05 | 1.05E+05 | 1.09E+06 | 6.74E+05 |
| Meta82 | 1.92E+02 | C7H12O6 | Kinic acid | Organic acids | Organic acids | 77-95-2 | A | 191.06, 85.03, 93.03, 87.01, 59.01 | 5.45E+05 | 2.44E+06 | 2.31E+05 | 1.11E+06 | 1.08E+06 | 1.09E+06 |
| Meta17 | 3.12E+02 | C18H32O4 | 13-Oxo-9-hydroxy-10-octadecenoic acid | Lipids | Free fatty acids | - | B | 311.22, 293.21, 223.13, 125.09 | 9.00E+00 | 6.20E+04 | 4.14E+06 | 9.46E+04 | 1.07E+06 | 9.34E+05 |
| Meta268 | 5.20E+02 | C24H24O13 | Isorhamnetin O-acetyl-hexoside | Flavonoids | Flavonols | - | A | 519.11, 361.09, 343.09, 361.1, 361.09 | 3.91E+06 | 2.93E+03 | 2.70E+05 | 1.38E+03 | 1.05E+06 | 8.79E+05 |
| Meta24 | 1.44E+02 | C7H14NO2+ | Proline βine(ProBet) | Amino acids and derivatives | Amino acids and derivatives | 1195-94-4 | B | 144.1, 84.08, 55 | 2.68E+06 | 2.81E+05 | 1.00E+06 | 1.54E+05 | 1.03E+06 | 7.91E+05 |
| Meta87 | 1.66E+02 | C5H10O6 | D-Xylonic acid | Organic acids | Organic acids | 526-91-0 | A | 165.04, 75.01, 59.01, 71.01, 165.04 | 1.47E+06 | 9.40E+05 | 1.08E+06 | 5.16E+05 | 1.00E+06 | 1.85E+06 |
| Meta114 | 1.38E+02 | C7H6O3 | 4-Hydroxybenzoic acid | Phenolic acids | Phenolic acids | 99-96-7 | A | 137.03, 136.02, 108.02, 81.04, 92.03 | 1.79E+05 | 2.75E+05 | 3.29E+06 | 1.65E+05 | 9.76E+05 | 7.52E+05 |
| Meta10 | 5.20E+02 | C24H24O13 | Isorhamnetin acetyl hexoside | Flavonoids | Flavonols | - | A | 519.11, 361.09, 343.09, 361.1, 361.09 | 3.64E+06 | 9.00E+00 | 2.39E+05 | 9.00E+00 | 9.70E+05 | 7.68E+05 |
| Meta136 | 5.94E+02 | C27H30O15 | Apigenin 6,8-C-diglucoside | Flavonoids | Flavonoid | 23666-13-9 | A | 595.17, 457.11, 577.15, 595.17, 559.15 | 1.67E+06 | 1.10E+06 | 8.65E+04 | 9.24E+05 | 9.44E+05 | 7.95E+05 |
| Meta210 | 3.06E+02 | C15H22N4O3 | N-Feruloyl agmatine | Alkaloids | Phenolamine | - | B | 307.18, 177.06, 145.03, 114.1, 290.15 | 4.57E+04 | 9.00E+00 | 3.72E+06 | 4.80E+03 | 9.42E+05 | 8.66E+05 |
| Meta315 | 2.67E+02 | C10H13N5O4 | Deoxyguanosine | Nucleotides and derivatives | Nucleotides and derivatives | 961-07-9 | B | 268.1, 136.06, 119.04, 135.65, 57.03 | 9.47E+04 | 8.48E+05 | 1.46E+06 | 1.27E+06 | 9.20E+05 | 6.68E+05 |
| Meta347 | 1.62E+02 | C6H10O5 | 3-Hydroxy-3-methylpentane-1,5-dioic acid | Amino acids and derivatives | Amino acids and derivatives | 503-49-1 | A | 161.05, 57.03, 99.04, 59.01, 101.02 | 1.41E+06 | 9.00E+00 | 2.13E+06 | 1.37E+05 | 9.19E+05 | 1.56E+06 |
| Meta243 | 3.47E+02 | C10H14N5O7P | Adenosine 5'-monophosphate | Nucleotides and derivatives | Nucleotides and derivatives | 61-19-8 | A | 348.2, 136, 97.03, 119.03 | 1.71E+06 | 6.68E+05 | 2.72E+05 | 1.02E+06 | 9.17E+05 | 8.57E+05 |
| Meta377 | 3.30E+02 | C15H22O8 | 3,4,5-Trimethoxyphenyl-β-D-Glucopyranoside | Phenolic acids | Phenolic acids | - | A | 329.08, 123.04, 149.02, 167.03, 169.01 | 1.52E+06 | 1.04E+06 | 3.42E+05 | 6.90E+05 | 8.99E+05 | 9.54E+05 |
| Meta340 | 1.83E+02 | C8H9NO4 | 4-Pyridoxic acid | Others | Vitamin | 82-82-6 | B | 184.06, 148.04, 166.05, 138.06, 120.04 | 2.11E+06 | 3.57E+05 | 9.43E+05 | 1.41E+05 | 8.88E+05 | 1.08E+06 |
| Meta212 | 6.63E+02 | C21H27N7O14P2 | Nicotinic acid adenine dinucleotide | Nucleotides and derivatives | Nucleotides and derivatives | 53-84-9 | B | 664.12, 428.05, 542.06, 428.03, 524.05 | 8.01E+05 | 1.08E+06 | 1.69E+05 | 1.48E+06 | 8.80E+05 | 7.89E+05 |
| Meta224 | 6.10E+02 | C27H30O16 | 8-C-Hexosyl-luteolin O-hexoside | Flavonoids | Flavonoid carbonoside | - | B | 609.15, 609.14, 301.04, 300.03, 607.25 | 2.99E+06 | 5.61E+03 | 5.23E+05 | 1.48E+03 | 8.79E+05 | 7.04E+05 |
| Meta356 | 3.83E+02 | C14H17N5O8 | N6-Succinyl Adenosine | Nucleotides and derivatives | Nucleotides and derivatives | 4542-23-8 | B | 384.12, 252.07, 234.06, 162.08, 136.06 | 4.98E+05 | 1.53E+06 | 3.85E+05 | 1.02E+06 | 8.60E+05 | 9.86E+05 |
| Meta318 | 2.20E+02 | C11H12N2O3 | 5-Hydroxy-L-tryptophan | Amino acids and derivatives | Amino acids and derivatives | 56-69-9 | B | 221.09, 162.06, 134.06, 204.07, 160.08 | 7.50E+05 | 1.56E+05 | 1.80E+06 | 6.09E+05 | 8.29E+05 | 4.28E+05 |
| Meta160 | 3.58E+02 | C16H22O9 | Sweroside | Terpenoids | Sesquiterpenoids | 14215-86-2 | B | 359.13, 127.04, 197.08, 179.07, 111.08 | 9.87E+05 | 6.07E+04 | 2.13E+06 | 5.42E+04 | 8.07E+05 | 1.89E+06 |
| Meta130 | 3.40E+02 | C15H16O9 | Esculin(6,7-DihydroxyCoumarin-6-glucoside) | Lignans and Coumarins | Coumarins | 531-75-9 | B | 339.07, 177.02, 133.03, 176.01, 176.48 | 2.94E+06 | 8.50E+04 | 9.38E+04 | 4.58E+04 | 7.90E+05 | 6.05E+05 |
| Meta328 | 1.46E+02 | C7H16NO2 | Acetylcholine | Alkaloids | Alkaloids | 60-31-1 | B | 147.12, 87.2, 60.08 | 9.72E+05 | 8.97E+05 | 3.42E+05 | 9.27E+05 | 7.84E+05 | 1.51E+06 |
| Meta208 | 2.34E+02 | C13H18N2O2 | N-p-Coumaroyl putrescine | Alkaloids | Phenolamine | 34136-53-3 | B | 235.14, 147.05, 119.05, 72.08, 91.06 | 2.59E+04 | 9.00E+00 | 3.05E+06 | 7.19E+03 | 7.71E+05 | 8.51E+05 |
| Meta49 | 5.23E+02 | C26H54NO7P | 1-Stearoyl-sn-glycero-3-phosphocholine | Lipids | LPC | 19420-57-6 | B | 524.37, 184.08, 104.11, 506.36, 184.1 | 7.45E+05 | 3.08E+05 | 1.41E+05 | 1.87E+06 | 7.67E+05 | 6.50E+05 |
| Meta418 | 3.13E+02 | C18H19NO4 | Methoxy-N-Caffeoyltyramine | Alkaloids | Phenolamine | - | B | 314.1, 145, 177.06, 121.06 | 6.21E+05 | 1.29E+05 | 1.97E+06 | 3.36E+05 | 7.65E+05 | 6.68E+05 |
| Meta431 | 5.23E+02 | C26H54NO7P | LysoPC(18:0) | Lipids | LPC | - | B | 524.37, 184.08, 104.11, 506.36, 184.1 | 6.98E+05 | 2.92E+05 | 1.46E+05 | 1.91E+06 | 7.61E+05 | 6.33E+05 |
| Meta178 | 1.80E+02 | C9H8O4 | Caffeic acid | Phenolic acids | Phenolic acids | 331-39-5 | A | 179.03, 135.05, 134.04, 107.05, 117.04 | 3.42E+05 | 2.17E+06 | 9.00E+00 | 4.77E+05 | 7.48E+05 | 1.37E+06 |
| Meta209 | 5.83E+02 | C34H37N3O6 | N',N'',N'''-p-Coumaroyl-cinnamoyl-caffeoyl spermidine | Alkaloids | Phenolamine | - | B | 584.2, 325, 454.14, 144.08 | 2.98E+04 | 1.93E+04 | 2.86E+06 | 9.37E+03 | 7.31E+05 | 5.52E+05 |
| Meta162 | 1.31E+02 | C5H9NO3 | Cis-4-Hydroxy-D-proline | Amino acids and derivatives | Amino acids and derivatives | 2584-71-6 | A | 132.07, 73, 86, 68 | 3.06E+05 | 1.27E+06 | 1.87E+05 | 1.10E+06 | 7.16E+05 | 3.18E+06 |
| Meta23 | 6.22E+02 | C30H38O14 | Syringaresinol-aceGlu | Lignans and Coumarins | Lignans | - | B | 621.21, 417.16, 517.3, 345.19 | 2.13E+06 | 2.29E+05 | 1.84E+05 | 2.31E+05 | 6.93E+05 | 6.39E+05 |
| Meta30 | 2.70E+02 | C15H10O5 | Baicalein | Flavonoids | Flavonoid | 491-67-8 | B | 271.06, 123.01, 253.05, 169.01, 225.05 | 2.26E+06 | 1.33E+04 | 4.87E+05 | 1.13E+04 | 6.92E+05 | 8.89E+05 |
| Meta196 | 3.40E+02 | C15H16O9 | Esculin Hydrate | Lignans and Coumarins | Coumarins | 66778-17-4 | B | 339.07, 177.02, 133.03, 176.01, 176.48 | 2.27E+06 | 2.29E+05 | 9.00E+00 | 1.36E+05 | 6.58E+05 | 5.09E+05 |
| Meta72 | 1.74E+02 | C8H14O4 | SubericAcid | Organic acids | Organic acids | 505-48-6 | B | 173.08, 111.08, 83.05, 109.06, 57.03 | 7.31E+05 | 1.76E+05 | 9.44E+05 | 7.68E+05 | 6.55E+05 | 4.87E+05 |
| Meta310 | 3.07E+02 | C10H17N3O6S | Glutathione reduced form | Amino acids and derivatives | Amino acids and derivatives | 70-18-8 | A | 306.08, 143.05, 128.03, 160.01, 210.09 | 7.34E+05 | 4.11E+05 | 1.22E+05 | 1.35E+06 | 6.54E+05 | 5.59E+05 |
| Meta323 | 1.64E+02 | C9H8O3 | p-Coumaric acid | Phenolic acids | Phenolic acids | 501-98-4 | A | 165.06, 119.05, 91.06, 147.05, 65.04 | 5.04E+04 | 1.33E+06 | 6.44E+05 | 5.68E+05 | 6.48E+05 | 3.27E+05 |
| Meta336 | 2.44E+02 | C10H16N2O3S | Biotin | Others | Vitamin | 58-85-5 | B | 245.1, 227.09, 123.03, 184.08, 166.07 | 1.58E+06 | 3.67E+05 | 4.78E+05 | 1.48E+05 | 6.43E+05 | 6.69E+05 |
| Meta274 | 1.54E+02 | C4H11O4P | Diethyl phosphate | Organic acids | Organic acids | - | - | - | 1.19E+06 | 3.44E+05 | 6.66E+05 | 3.55E+05 | 6.38E+05 | 7.44E+05 |
| Meta238 | 4.77E+02 | C23H44NO7P | LysoPE 18:2(2n isomer) | Lipids | LPE | - | A | 478.29, 337.28, 306.28, 263.24, 81.07 | 1.36E+06 | 2.54E+05 | 2.02E+05 | 7.22E+05 | 6.34E+05 | 6.44E+05 |
| Meta60 | 1.54E+02 | C7H6O4 | Protocatechuic acid | Flavonoids | Flavanols | 99-50-3 | A | 153.02, 109.03, 108.02, 109.04, 91.02 | 5.50E+05 | 9.63E+05 | 6.57E+05 | 3.35E+05 | 6.26E+05 | 6.39E+05 |
| Meta59 | 3.68E+02 | C17H20O9 | Chlorogenic acid methyl ester | Phenolic acids | Phenolic acids | 29708-87-0 | A | 367.1, 179.04, 135.05, 161.02, 134.04 | 1.09E+06 | 9.00E+00 | 9.41E+05 | 4.52E+05 | 6.21E+05 | 5.14E+05 |
| Meta214 | 4.49E+02 | C21H21O11 | Cyanidin 3-O-glucoside(Kuromanin) | Flavonoids | Anthocyanins | 7084-24-4 | B | 449.11, 287.06, 149.02, 256.96, 136.08 | 3.52E+04 | 9.00E+00 | 2.39E+06 | 5.97E+04 | 6.21E+05 | 6.31E+05 |
| Meta433 | 6.26E+02 | C27H30O17 | 6-Hydroxykaempferol-3,6-O-Diglucoside | Flavonoids | Flavonols | - | B | 627.16, 303.05, 465.1, 145.05, 85.03 | 8.67E+05 | 8.21E+05 | 3.29E+05 | 4.17E+05 | 6.09E+05 | 5.75E+05 |
| Meta205 | 1.35E+02 | C5H5N5 | Aminopurine | Alkaloids | Alkaloids | 452-06-2 | A | 136.06, 119.04, 136.06, 92.03, 94.04 | 8.92E+04 | 1.54E+04 | 2.18E+06 | 1.06E+04 | 5.73E+05 | 4.70E+05 |
| Meta379 | 1.54E+02 | C7H6O4 | Gentisic acid | Phenolic acids | Phenolic acids | 490-79-9 | A | 153.02, 109.03, 108.02, 109.04, 91.02 | 4.95E+05 | 8.84E+05 | 5.96E+05 | 3.16E+05 | 5.73E+05 | 5.63E+05 |
| Meta121 | 2.35E+02 | C5H9NaO7P | 2-Deoxyribose 5-phosphate | Nucleotides and derivatives | Nucleotides and derivatives | 102916-66-5 | B | 213.02, 78.96, 96.97, 78.72, 195.01 | 1.16E+05 | 3.29E+03 | 2.14E+06 | 1.76E+04 | 5.68E+05 | 2.82E+05 |
| Meta93 | 2.82E+02 | C18H34O2 | Elaidic Acid | Lipids | Free fatty acids | 112-79-8 | B | 281.25, 280.37, 280.39, 263.25, 280.43 | 2.75E+05 | 3.60E+05 | 8.49E+05 | 7.41E+05 | 5.56E+05 | 8.13E+05 |
| Meta73 | 2.44E+02 | C9H12N2O6 | Uridine | Nucleotides and derivatives | Nucleotides and derivatives | 58-96-8 | A | 243.06, 110.02, 82.03, 152.04, 122.02 | 5.45E+04 | 1.60E+05 | 1.88E+06 | 1.20E+05 | 5.53E+05 | 4.22E+05 |
| Meta118 | 1.49E+02 | C6H7N5 | 1-Methyladenine | Nucleotides and derivatives | Nucleotides and derivatives | 5142-22-3 | B | 150.08, 133.05, 109.05, 119.04, 135.05 | 1.02E+06 | 2.90E+05 | 7.18E+05 | 1.46E+05 | 5.44E+05 | 6.06E+05 |
| Meta133 | 4.48E+02 | C21H20O11 | Cyanidin 3-O-galactoside | Flavonoids | Anthocyanins | 27661-36-5 | A | 447.09, 285.04, 284.03, 284.05, 327.05 | 7.59E+04 | 9.00E+00 | 2.07E+06 | 2.31E+04 | 5.43E+05 | 5.01E+05 |
| Meta217 | 7.70E+02 | C34H42O20 | Chrysoeriol O-hexosyl-O-rutinoside | Flavonoids | Flavonoid | - | B | 771.23, 463.12, 609.19, 301.07, 625.18 | 1.64E+06 | 1.43E+04 | 5.08E+05 | 7.94E+03 | 5.43E+05 | 1.84E+07 |
| Meta429 | 3.54E+02 | C21H38O4 | Glyceryl linoleate | Lipids | Glycerol ester | 26545-74-4 | A | 355.29, 263.24, 245.23, 337.27, 95.09 | 9.71E+05 | 2.11E+05 | 1.66E+05 | 8.18E+05 | 5.42E+05 | 5.42E+05 |
| Meta183 | 2.21E+02 | C8H15NO6 | N-Acetyl-D-galactosamine | Others | Others | - | - | - | 5.28E+05 | 4.97E+05 | 5.42E+05 | 5.42E+05 | 5.27E+05 | 5.45E+05 |
| Meta236 | 4.25E+02 | C19H40NO7P | LysoPE 14:0 | Lipids | LPE | - | A | 426.26, 285.24, 254.25, 365.21, 62.06 | 3.00E+05 | 6.10E+05 | 2.64E+04 | 1.16E+06 | 5.25E+05 | 3.95E+05 |
| Meta110 | 1.54E+02 | C7H6O4 | 2,3-Dihydroxybenzoic Acid | Organic acids | Organic acids | 303-38-8 | B | 153.02, 109.03, 108.02, 109.04, 91.02 | 4.31E+05 | 8.12E+05 | 5.45E+05 | 2.68E+05 | 5.14E+05 | 5.20E+05 |
| Meta402 | 3.38E+02 | C16H18O8 | Trans-3-O-p-coumaric quinic acid | Phenolic acids | Phenolic acids | - | B | 337.09, 163.04, 191.06, 119.05, 173.05 | 1.37E+06 | 4.31E+05 | 1.17E+05 | 1.16E+05 | 5.09E+05 | 2.80E+05 |
| Meta32 | 5.78E+02 | C27H30O14 | Rhoifolin | Flavonoids | Flavonoid | 17306-46-6 | B | 579.17, 271.07, 271.08, 433.11, 270.23 | 9.39E+05 | 5.87E+05 | 3.52E+05 | 1.45E+05 | 5.06E+05 | 2.71E+05 |
| Meta198 | 1.88E+02 | C8H16N2O3 | Glycylisoleucine | Amino acids and derivatives | Amino acids and derivatives | 19461-38-2 | B | 189.12, 86.1, 132.1, 143.12, 69.07 | 1.33E+05 | 2.39E+05 | 1.45E+06 | 1.49E+05 | 4.92E+05 | 3.98E+05 |
| Meta27 | 2.81E+02 | C18H35NO | Octadecenoic amide | Others | Others | - | - | - | 4.25E+05 | 3.79E+05 | 6.96E+05 | 4.30E+05 | 4.82E+05 | 4.60E+05 |
| Meta126 | 1.46E+02 | C6H10O4 | 2-Methylglutaric acid | Organic acids | Organic acids | 617-62-9 | B | 145.05, 83.05, 101.06, 81.03, 145.05 | 2.04E+05 | 2.00E+05 | 3.62E+05 | 1.14E+06 | 4.77E+05 | 3.75E+05 |
| Meta428 | 5.43E+02 | C28H50NO7P | Propyl2-(trimethylammonio)ethyl phosphate | Others | Others | - | A | 544.34, 485.27, 544.34, 104.11, 146.98 | 2.73E+05 | 3.47E+05 | 7.74E+04 | 1.17E+06 | 4.68E+05 | 5.62E+05 |
| Meta292 | 1.51E+02 | C5H5N5O | 2-Hydroxy-6-aminopurine | Nucleotides and derivatives | Nucleotides and derivatives | 3373-53-3 | B | 152.06, 135.03, 110.04, 55.03, 82.04 | 2.38E+05 | 1.84E+05 | 1.20E+06 | 2.25E+05 | 4.62E+05 | 3.89E+05 |
| Meta61 | 1.17E+02 | C5H11NO2 | Betaine | Alkaloids | Alkaloids | - | - | 118.09, 72.08, 55.06, 57.06, 56.05 | 4.30E+05 | 5.16E+05 | 3.81E+05 | 5.10E+05 | 4.59E+05 | 3.54E+05 |
| Meta199 | 2.22E+02 | C11H14N2O3 | Glycylphenylalanine | Amino acids and derivatives | Amino acids and derivatives | 721-66-4 | A | 223.11, 120.08, 166.09, 120.1, 103.06 | 1.50E+05 | 2.13E+05 | 1.16E+06 | 2.92E+05 | 4.55E+05 | 7.89E+05 |
| Meta137 | 1.76E+02 | C10H8O3 | 7-Methoxycoumarin | Lignans and Coumarins | Coumarins | 531-59-9 | A | 177.06, 121.07, 133.07, 118.04, 134.04 | 1.29E+06 | 2.22E+05 | 7.79E+04 | 2.10E+05 | 4.49E+05 | 6.09E+05 |
| Meta64 | 1.46E+02 | C6H10O4 | Adipic Acid | Organic acids | Organic acids | 124-04-9 | B | 145.05, 83.05, 101.06, 81.03, 145.05 | 2.08E+05 | 1.86E+05 | 3.34E+05 | 1.06E+06 | 4.47E+05 | 3.63E+05 |
| Meta365 | 1.62E+02 | C10H10O2 | 4-MethoxycinnaMaldehyde | Phenolic acids | Phenolic acids | - | - | - | 5.09E+05 | 2.92E+05 | 5.95E+05 | 3.91E+05 | 4.47E+05 | 4.81E+05 |
| Meta53 | 1.18E+02 | C5H10O3 | 3-Hydroxy-3-methyl butyric acid | Organic acids | Organic acids | 625-08-1 | B | 117.06, 99.92, 59.01, 116.93 | 2.85E+05 | 1.28E+05 | 1.19E+05 | 1.24E+06 | 4.43E+05 | 3.19E+05 |
| Meta348 | 4.04E+02 | C9H14N2O12P2 | Uridine 5’-diphosphate | Nucleotides and derivatives | Nucleotides and derivatives | 27821-45-0 | B | 402.99, 158.92, 402.99, 111.02, 384.98 | 1.48E+05 | 5.86E+05 | 4.20E+05 | 5.98E+05 | 4.38E+05 | 3.46E+05 |
| Meta80 | 1.66E+02 | C9H10O3 | Phenyllactate(PLA) | Organic acids | Organic acids | 828-01-3 | A | 165.06, 59.01, 93.03, 121.07, 119.05 | 1.23E+06 | 5.28E+04 | 4.32E+05 | 9.00E+00 | 4.29E+05 | 8.88E+05 |
| Meta353 | 3.31E+02 | C10H14N5O6P | 2'-Deoxyadenosine-5'-monophosphate | Nucleotides and derivatives | Nucleotides and derivatives | 653-63-4 | B | 332.08, 136.06, 81.03, 119.04, 98.98 | 1.76E+05 | 3.23E+05 | 4.59E+05 | 7.43E+05 | 4.25E+05 | 3.74E+05 |
| Meta257 | 2.94E+02 | C18H30O3 | 9-HOTrE | Lipids | Free fatty acids | 89886-42-0 | A | 293.21, 275.2, 235.17, 113.1, 223.17 | 7.93E+04 | 4.86E+03 | 1.60E+06 | 7.13E+03 | 4.23E+05 | 3.62E+05 |
| Meta288 | 1.17E+02 | C5H11NO2 | 5-Aminovaleric acid | Amino acids and derivatives | Amino acids and derivatives | 660-88-8 | B | 118.09, 72.08, 55.06, 57.06, 56.05 | 1.09E+06 | 3.87E+04 | 4.76E+05 | 1.36E+04 | 4.04E+05 | 3.43E+05 |
| Meta410 | 6.26E+02 | C27H30O17 | Quercetin 3,7-bis-O-β-D-glucoside | Flavonoids | Flavonols | - | B | 627.16, 303.05, 465.1, 145.05, 85.03 | 4.18E+05 | 2.85E+05 | 5.14E+05 | 3.22E+05 | 3.85E+05 | 5.45E+05 |
| Meta211 | 2.76E+02 | C14H20N4O2 | N-p-Coumaroyl agmatine | Alkaloids | Phenolamine | 7295-86-5 | A | 277.17, 147.05, 114.1, 218.12, 119.05 | 3.72E+02 | 2.57E+02 | 1.52E+06 | 1.10E+03 | 3.80E+05 | 3.22E+05 |
| Meta316 | 2.27E+02 | C9H13N3O4 | Deoxycytidine | Nucleotides and derivatives | Nucleotides and derivatives | 951-77-9 | B | 228.1, 112.05, 95.03, 117.06, 94.04 | 3.72E+04 | 3.20E+05 | 8.79E+05 | 2.28E+05 | 3.66E+05 | 2.88E+05 |
| Meta158 | 2.80E+02 | C18H32O2 | Linoleic acid | Lipids | Free fatty acids | 60-33-3 | A | 279.24, 279.23, 261.22, 278.36, 59.02 | 2.06E+05 | 2.27E+05 | 6.97E+05 | 2.79E+05 | 3.52E+05 | 3.53E+05 |
| Meta135 | 2.68E+02 | C10H12N4O5 | 9-(β-D-Arabinofuranosyl)hypoxanthine | Nucleotides and derivatives | Nucleotides and derivatives | 7013-16-3 | A | 267.08, 135.03, 267.07, 149.04, 177.04 | 1.03E+05 | 7.44E+05 | 1.12E+05 | 3.50E+05 | 3.27E+05 | 1.88E+05 |
| Meta104 | 1.25E+02 | C5H7N3O | 5-Methylcytosine | Nucleotides and derivatives | Nucleotides and derivatives | - | - | - | 4.36E+05 | 1.30E+05 | 5.46E+05 | 1.01E+05 | 3.03E+05 | 2.86E+05 |
| Meta96 | 1.52E+02 | C8H8O3 | Vanillin | Phenolic acids | Phenolic acids | 121-33-5 | B | 151.04, 77.04, 93.03, 107.05, 76.81 | 4.24E+05 | 8.82E+04 | 4.64E+05 | 2.02E+05 | 2.95E+05 | 3.47E+05 |
| Meta164 | 5.16E+02 | C25H24O12 | Cynarin | Phenolic acids | Phenolic acids | 30964-13-7 | A | 515.12, 353.09, 191.06, 179.04, 335.08 | 2.40E+05 | 6.00E+04 | 9.44E+04 | 7.83E+05 | 2.94E+05 | 1.95E+05 |
| Meta405 | 4.62E+02 | C22H22O11 | Diosmetin-7-O-galactoside | Flavonoids | Flavonoid | - | B | 463.12, 301.07, 286.05, 258.05, 300.14 | 9.71E+05 | 9.00E+00 | 1.90E+05 | 9.00E+00 | 2.90E+05 | 2.86E+05 |
| Meta228 | 3.01E+02 | C18H39NO2 | D-erythro-Dihydrosphingosine | Lipids | Sphingolipids | 764-22-7 | B | 302.4, 60.2, 284.3, 240.27 | 1.22E+05 | 2.50E+05 | 2.39E+05 | 5.49E+05 | 2.90E+05 | 2.29E+05 |
| Meta382 | 3.10E+02 | C20H38O2 | Eicosenoic acid | Lipids | Free fatty acids | 26764-41-0 | A | 309.28, 309.28, 308.32, 291.19, 134.89 | 4.49E+04 | 9.00E+00 | 9.68E+05 | 1.28E+05 | 2.85E+05 | 2.26E+05 |
| Meta338 | 1.38E+02 | C7H6O3 | Protocatechuic aldehyde | Flavonoids | Flavanols | 139-85-5 | A | 137.03, 136.02, 108.02, 81.04, 92.03 | 5.69E+04 | 7.60E+05 | 6.93E+04 | 2.24E+05 | 2.77E+05 | 1.57E+05 |
| Meta346 | 2.68E+02 | C8H16N2O4S2 | L-Homocystine | Amino acids and derivatives | Amino acids and derivatives | 626-72-2 | B | 269.06, 88.02, 90.04, 56.05, 134.03 | 2.99E+04 | 1.21E+04 | 1.03E+06 | 2.65E+04 | 2.75E+05 | 2.68E+05 |
| Meta03 | 5.94E+02 | C27H30O15 | Kaempferol-3-O-glucoside-7-O-rhamnoside | Flavonoids | Flavonols | - | A | 595.17, 457.11, 577.15, 595.17, 559.15 | 3.65E+05 | 1.86E+05 | 2.65E+05 | 2.73E+05 | 2.73E+05 | 1.76E+05 |
| Meta218 | 5.48E+02 | C25H24O14 | Chrysoeriol O-malonylhexoside | Flavonoids | Flavonoid | - | B | 549.12, 301.07, 463.12, 286.05, 531.1 | 7.11E+05 | 1.61E+04 | 3.20E+05 | 3.21E+04 | 2.70E+05 | 2.37E+05 |
| Meta403 | 2.83E+02 | C17H17NO3 | N-cis-Paprazine | Alkaloids | Phenolamine | - | A | 282.11, 119.05, 162.06 | 1.14E+04 | 7.44E+05 | 1.16E+03 | 3.12E+05 | 2.67E+05 | 1.69E+05 |
| Meta416 | 2.83E+02 | C17H17NO3 | N-cis-sinapoyltyramine | Alkaloids | Phenolamine | - | B | 282.11, 119.05, 162.06 | 1.16E+04 | 7.46E+05 | 1.05E+03 | 3.07E+05 | 2.66E+05 | 1.71E+05 |
| Meta08 | 6.82E+02 | C29H34N2O17 | 2'-O-Apiosylbetain | Alkaloids | Alkaloids | - | A | 683.2, 389.1 | 5.78E+04 | 9.00E+00 | 9.64E+04 | 8.95E+05 | 2.62E+05 | 1.46E+05 |
| Meta69 | 1.19E+02 | C4H9NO3 | L-(-)-Threonine | Amino acids and derivatives | Amino acids and derivatives | - | - | - | 2.41E+05 | 1.69E+05 | 3.22E+05 | 2.94E+05 | 2.57E+05 | 2.27E+05 |
| Meta235 | 4.91E+02 | C24H46NO7P | LysoPC 16:2(2n isomer) | Lipids | LPC | - | B | 492.31, 184.07, 104.11, 474.29, 320.25 | 1.83E+04 | 5.94E+04 | 1.57E+04 | 9.14E+05 | 2.52E+05 | 1.43E+05 |
| Meta11 | 5.64E+02 | C25H24O15 | Isorhamnetin hexose-malonate | Flavonoids | Flavonols | - | B | 563.1, 519.11, 315.05, 59.01 | 9.11E+05 | 9.00E+00 | 7.43E+04 | 9.00E+00 | 2.46E+05 | 2.07E+05 |
| Meta259 | 2.94E+02 | C18H30O3 | 13-HOTrE(r) | Lipids | Free fatty acids | - | - | 293.21, 275.2, 235.17, 113.1, 223.17 | 2.48E+05 | 1.16E+05 | 4.32E+05 | 1.39E+05 | 2.34E+05 | 2.54E+05 |
| Meta145 | 2.08E+02 | C11H12O4 | Methyl ferulate | Phenolic acids | Phenolic acids | 2309-07-1 | A | 209.08, 145.03, 117.03, 177.06, 149.06 | 1.83E+05 | 5.78E+04 | 6.43E+05 | 4.25E+04 | 2.32E+05 | 1.80E+05 |
| Meta107 | 1.22E+02 | C7H6O2 | 4-Hydroxybenzaldehyde | Phenolic acids | Phenolic acids | 123-08-0 | B | 121.03, 92.03, 93.03, 108.02, 120.02 | 2.56E+05 | 1.13E+05 | 4.33E+05 | 1.13E+05 | 2.29E+05 | 2.28E+05 |
| Meta394 | 3.28E+02 | C18H32O5 | 9,12,13-Trihyroxy-10,15-octadecadienoic acid | Lipids | Free fatty acids | - | B | 327.22, 211.14, 183.14, 171.1, 229.15 | 2.69E+04 | 1.00E+02 | 8.40E+05 | 9.74E+03 | 2.19E+05 | 1.42E+05 |
| Meta264 | 5.50E+02 | C25H26O14 | Hesperetin O-malonylhexoside | Flavonoids | Dihydroflavonol | - | B | 549.2, 387.1, 505.19, 175.03 | 6.75E+04 | 3.43E+05 | 9.00E+00 | 4.38E+05 | 2.12E+05 | 1.32E+05 |
| Meta343 | 1.30E+02 | C6H14N2O | N-Acetylputrescine | Alkaloids | Alkaloids | 18233-70-0 | B | 131, 114, 72.08 | 6.66E+05 | 1.31E+04 | 1.45E+05 | 1.11E+04 | 2.09E+05 | 1.76E+05 |
| Meta215 | 5.08E+02 | C23H24O13 | Syringetin 3-O-hexoside | Flavonoids | Flavonols | - | B | 509.1, 347.2, 329.11, 311.1 | 5.16E+05 | 5.78E+04 | 2.48E+05 | 9.00E+00 | 2.06E+05 | 2.10E+05 |
| Meta123 | 3.29E+02 | C10H12N5O6P | Cyclic AMP | Nucleotides and derivatives | Nucleotides and derivatives | 60-92-4 | A | 328.05, 134.05, 78.96, 192.99, 134.99 | 9.00E+00 | 9.00E+00 | 8.21E+05 | 7.49E+02 | 2.06E+05 | 1.42E+05 |
| Meta188 | 6.12E+02 | C20H32N6O12S2 | Oxidized Glutathione | Amino acids and derivatives | Amino acids and derivatives | - | - | - | 1.36E+05 | 2.41E+05 | 2.43E+05 | 1.98E+05 | 2.05E+05 | 2.24E+05 |
| Meta393 | 3.28E+02 | C16H24O7 | 3-Hydroxy-4-isopropylbenzylalcohol 3-glucoside | Phenolic acids | Phenolic acids | - | B | 327.15, 164.08, 165.09, 149.06, 134.04 | 7.19E+04 | 1.66E+05 | 6.43E+04 | 5.11E+05 | 2.04E+05 | 9.97E+04 |
| Meta322 | 4.65E+02 | C21H21O12 | Delphinidin 3-O-glucoside(Mirtillin) | Flavonoids | Anthocyanins | - | - | - | 2.90E+04 | 3.65E+04 | 7.24E+05 | 1.73E+04 | 2.02E+05 | 2.33E+05 |
| Meta371 | 1.88E+02 | C9H16O4 | Eucommiol | Others | Others | 55930-44-4 | B | 187.1, 125.1, 187.1, 97.06, 123.08 | 4.98E+04 | 2.68E+04 | 6.70E+05 | 4.53E+04 | 1.98E+05 | 1.25E+05 |
| Meta102 | 2.23E+02 | C11H13NO4 | N-Acetyl-L-tyrosine | Amino acids and derivatives | Amino acids and derivatives | 537-55-3 | A | 224.09, 136.08, 119.05, 123.04, 165.05 | 5.18E+05 | 5.41E+04 | 1.64E+05 | 3.99E+04 | 1.94E+05 | 2.34E+05 |
| Meta206 | 1.94E+02 | C10H10O4 | Hydroxy-methoxycinnamate | Phenolic acids | Phenolic acids | - | A | 193.05, 134.04, 178.03, 133.03, 137.03 | 1.68E+05 | 8.32E+03 | 5.69E+05 | 2.30E+04 | 1.92E+05 | 1.24E+05 |
| Meta154 | 3.03E+02 | C12H21N3O6 | Nicotianamine | Amino acids and derivatives | Amino acids and derivatives | 34441-14-0 | B | 304.15, 185.09, 286.14, 114.06, 141.1 | 2.13E+05 | 3.70E+05 | 3.00E+04 | 1.53E+05 | 1.91E+05 | 1.97E+05 |
| Meta414 | 5.94E+02 | C27H30O15 | Vitexin-2-O-D-glucopyranoside | Flavonoids | Flavonoid carbonoside | - | B | 595.17, 457.11, 577.15, 595.17, 559.15 | 3.33E+05 | 2.26E+05 | 1.54E+04 | 1.90E+05 | 1.91E+05 | 1.64E+05 |
| Meta147 | 2.70E+02 | C13H18O6 | Benzyl β-D-Glucopyranoside | Phenolic acids | Phenolic acids | 4304-12-5 | A | 269.1, 59.01, 71.01, 85.03, 101.02 | 6.20E+05 | 8.54E+04 | 4.33E+04 | 1.32E+04 | 1.91E+05 | 2.27E+05 |
| Meta220 | 6.26E+02 | C28H34O16 | 8-C-Hexosyl-hesperetin O-hexoside | Flavonoids | Flavonoid carbonoside | - | B | 627.1, 465.1, 447.09, 429.08 | 2.25E+05 | 1.27E+05 | 2.41E+05 | 1.59E+05 | 1.88E+05 | 2.69E+05 |
| Meta55 | 2.87E+02 | C15H11O6 | Cyanidin chloride | Flavonoids | Anthocyanins | 528-58-5 | B | 287, 213, 137, 227.03 | 4.45E+05 | 5.38E+04 | 2.10E+05 | 2.12E+04 | 1.82E+05 | 7.41E+04 |
| Meta134 | 1.47E+02 | C5H9NO4 | O-Acetylserine | Amino acids and derivatives | Amino acids and derivatives | 5147-00-2 | B | 148.06, 60.05, 88.04, 70.03, 106.05 | 2.53E+05 | 1.26E+05 | 2.16E+05 | 1.22E+05 | 1.79E+05 | 2.06E+05 |
| Meta184 | 2.82E+02 | C18H34O2 | 11-Octadecanoic acid(Vaccenic acid) | Lipids | Free fatty acids | 506-17-2 | A | 281.25, 280.37, 280.39, 263.25, 280.43 | 8.39E+04 | 1.05E+05 | 2.67E+05 | 2.22E+05 | 1.69E+05 | 2.34E+05 |
| Meta37 | 3.72E+02 | C20H20O7 | Tangeretin | Flavonoids | Flavonols | 481-53-8 | B | 373.13, 325.07, 343.1, 343.08, 358.12 | 3.57E+05 | 2.26E+05 | 6.41E+04 | 1.65E+04 | 1.66E+05 | 1.82E+05 |
| Meta320 | 1.84E+02 | C8H8O5 | DL-3,4-Dihydroxymandelic acid | Phenolic acids | Phenolic acids | 775-01-9 | B | 183.03, 137.02, 136.02, 109.03, 108.02 | 3.68E+05 | 5.64E+04 | 2.05E+05 | 2.69E+04 | 1.64E+05 | 1.24E+05 |
| Meta16 | 5.92E+02 | C29H36O13 | (+)-Medioresinol-aceGlu | Lignans and Coumarins | Lignans | - | B | 591.21, 387.14, 217.01, 151.04 | 5.88E+05 | 6.05E+04 | 9.00E+00 | 9.00E+00 | 1.62E+05 | 1.17E+05 |
| Meta42 | 4.48E+02 | C21H20O11 | Kaempferol 7-O-glucosdie | Flavonoids | Flavonols | 16290-07-6 | A | 447.09, 285.04, 284.03, 284.05, 327.05 | 4.00E+05 | 2.35E+04 | 2.13E+05 | 9.99E+03 | 1.62E+05 | 1.17E+05 |
| Meta194 | 2.02E+02 | C9H18N2O3 | Alanylleucine | Amino acids and derivatives | Amino acids and derivatives | 1999-42-4 | B | 203.14, 86.1, 132.1, 157.13 | 4.55E+05 | 2.88E+04 | 1.47E+05 | 1.33E+04 | 1.61E+05 | 1.98E+05 |
| Meta354 | 3.24E+02 | C9H13N2O9P | Uridine 5'-monophosphate | Nucleotides and derivatives | Nucleotides and derivatives | 58-97-9 | A | 323.03, 78.96, 96.97, 323.03, 111.02 | 9.37E+04 | 3.92E+04 | 4.79E+05 | 3.01E+04 | 1.60E+05 | 1.31E+05 |
| Meta108 | 2.80E+02 | C13H16N2O5 | Asp-phe | Amino acids and derivatives | Amino acids and derivatives | 13433-09-5 | A | 281.11, 120.08, 166.09, 200.07, 175.09 | 2.86E+05 | 1.14E+05 | 1.55E+05 | 8.20E+04 | 1.59E+05 | 2.51E+05 |
| Meta384 | 5.20E+02 | C23H20O14 | Quercetin 3-O-β-(2''-O-acetylβ-D-glucuronide) | Flavonoids | Flavonols | - | B | 519.08, 475.09, 300.03, 301.03, 271.02 | 5.83E+05 | 5.61E+02 | 4.52E+04 | 2.65E+02 | 1.57E+05 | 1.32E+05 |
| Meta270 | 4.82E+02 | C22H26O12 | 5-O-p-Coumaroyl shikimic acid O-hexoside | Phenolic acids | Phenolic acids | - | B | 481.13, 319.08, 163.04, 155.03, 113.03 | 9.00E+00 | 9.00E+00 | 4.58E+04 | 5.75E+05 | 1.55E+05 | 3.33E+06 |
| Meta155 | 1.61E+02 | C9H7NO2 | Indole-3-carboxylic acid | Alkaloids | Plumerane | 771-50-6 | B | 160.04, 116.05, 160.04, 115.7, 116.72 | 1.01E+05 | 1.49E+05 | 2.42E+05 | 1.19E+05 | 1.53E+05 | 1.23E+05 |
| Meta289 | 1.88E+02 | C8H16N2O3 | N6-Acetyl-L-lysine | Amino acids and derivatives | Amino acids and derivatives | 692-04-6 | B | 189.12, 86.1, 132.1, 143.12, 69.07 | 3.76E+05 | 1.04E+05 | 5.20E+04 | 7.95E+04 | 1.53E+05 | 1.87E+05 |
| Meta363 | 1.78E+02 | C10H10O3 | Riboprine | Phenolic acids | Phenolic acids | 7724-76-7 | B | 177, 145, 117.04 | 1.74E+05 | 1.07E+05 | 2.01E+05 | 1.20E+05 | 1.51E+05 | 1.73E+05 |
| Meta290 | 1.88E+02 | C7H12N2O4 | N-α-Acetyl-L-glutamine | Amino acids and derivatives | Amino acids and derivatives | - | - | - | 1.78E+05 | 1.05E+05 | 1.39E+05 | 1.79E+05 | 1.50E+05 | 1.46E+05 |
| Meta163 | 3.74E+02 | C16H22O10 | Geniposidic acid | Terpenoids | Sesquiterpenoids | 27741-01-1 | B | 373.12, 123.05, 149.06, 211.06, 167.07 | 4.48E+04 | 1.27E+05 | 9.26E+04 | 3.35E+05 | 1.50E+05 | 1.14E+05 |
| Meta177 | 4.48E+02 | C21H20O11 | Astragalin | Flavonoids | Flavonols | 480-10-4 | A | 447.09, 285.04, 284.03, 284.05, 327.05 | 3.75E+05 | 2.75E+04 | 1.84E+05 | 5.16E+03 | 1.48E+05 | 1.88E+05 |
| Meta187 | 2.24E+02 | C11H12O5 | Sinapic acid | Phenolic acids | Phenolic acids | 530-59-6 | A | 223.06, 193.01, 149.02, 208.04, 164.05 | 3.06E+04 | 2.24E+04 | 4.45E+05 | 7.38E+04 | 1.43E+05 | 1.18E+05 |
| Meta197 | 3.64E+02 | C12H21O11Na | Turanose | Others | Saccharides and Alcohols | 547-25-1 | B | 365.11, 203.05, 275.07 | 2.50E+05 | 9.00E+00 | 1.59E+05 | 1.59E+05 | 1.42E+05 | 2.66E+05 |
| Meta248 | 3.42E+02 | C19H38N2O3 | Cocamidopropyl βine | Alkaloids | Alkaloids | 4292-10-8 | A | 343.3, 240.23, 183.17, 343.28, 109.1 | 8.23E+04 | 1.01E+05 | 2.84E+05 | 8.24E+04 | 1.37E+05 | 1.21E+05 |
| Meta285 | 1.36E+02 | C5H4N4O | Hypoxanthine | Nucleotides and derivatives | Nucleotides and derivatives | 68-94-0 | B | 137, 119, 110.04, 55.03 | 1.35E+05 | 2.36E+04 | 1.65E+05 | 2.06E+05 | 1.33E+05 | 9.82E+04 |
| Meta01 | 7.72E+02 | C33H40O21 | Quercetin-O-rutinoside-hexose | Flavonoids | Flavonols | - | B | 771.13, 609.1, 463.05, 301.01 | 5.68E+04 | 1.53E+05 | 8.04E+04 | 2.27E+05 | 1.29E+05 | 2.22E+05 |
| Meta191 | 2.36E+02 | C12H16N2O3 | DL-Alanyl-DL-phenylalanine | Amino acids and derivatives | Amino acids and derivatives | 1999-45-7 | A | 237.12, 120.09, 166.09, 103.06, 131.05 | 7.21E+04 | 1.73E+04 | 3.97E+05 | 2.01E+04 | 1.27E+05 | 8.50E+04 |
| Meta105 | 3.21E+02 | C11H19N3O6S | S-(methyl)glutathione | Amino acids and derivatives | Amino acids and derivatives | 2922-56-7 | B | 322.11, 176.04, 130.05, 90.04, 84.05 | 7.62E+04 | 2.73E+05 | 1.91E+04 | 1.37E+05 | 1.26E+05 | 1.51E+05 |
| Meta116 | 3.09E+02 | C11H19NO9 | N-Acetylneuraminic acid | Amino acids and derivatives | Amino acids and derivatives | 131-48-6 | B | 308.1, 87.01, 170.04, 98.06, 119.04 | 1.47E+05 | 9.00E+00 | 3.49E+05 | 3.67E+03 | 1.25E+05 | 2.04E+04 |
| Meta179 | 1.48E+02 | C9H8O2 | Cinnamic acid | Phenolic acids | Phenolic acids | 140-10-3 | B | 147.05, 147.05, 119.05, 129.04, 117.04 | 2.65E+04 | 3.11E+05 | 1.28E+04 | 1.50E+05 | 1.25E+05 | 1.85E+05 |
| Meta303 | 2.90E+02 | C15H14O6 | L-Epicatechin | Flavonoids | Flavanols | 490-46-0 | B | 289.07, 289.07, 245.08, 203.07, 109.03 | 9.71E+03 | 4.31E+05 | 9.82E+03 | 4.60E+04 | 1.24E+05 | 3.85E+04 |
| Meta52 | 1.52E+02 | C8H8O3 | 2-Methoxybenzoic acid | Phenolic acids | Phenolic acids | 529-75-9 | B | 151.04, 77.04, 93.03, 107.05, 76.81 | 4.78E+05 | 5.79E+03 | 4.51E+02 | 1.05E+03 | 1.21E+05 | 8.70E+04 |
| Meta169 | 6.08E+02 | C28H32O15 | Diosmin | Flavonoids | Flavonoid | 520-27-4 | A | 609.18, 463.13, 301.07, 609.18, 129.05 | 2.70E+05 | 2.06E+04 | 1.20E+05 | 6.32E+04 | 1.19E+05 | 7.00E+04 |
| Meta242 | 2.64E+02 | C14H20N2O3 | N'-Feruloyl putrescine | Alkaloids | Phenolamine | - | A | 265.1, 177.5, 145.03, 117.03 | 1.68E+05 | 7.48E+02 | 2.99E+05 | 2.17E+03 | 1.18E+05 | 2.84E+06 |
| Meta18 | 3.14E+02 | C18H34O4 | 9,10-Dihydroxy-12-octadecenoic acid | Lipids | Free fatty acids | 263399-34-4 | B | 313.24, 295.23, 255.03, 171.1 | 6.62E+04 | 9.00E+00 | 3.97E+05 | 6.85E+03 | 1.17E+05 | 9.97E+04 |
| Meta185 | 3.16E+02 | C16H12O7 | Tamarixetin | Flavonoids | Flavonols | 603-61-2 | B | 315.05, 300.03, 315.05, 151, 271.03 | 1.86E+05 | 9.00E+00 | 2.83E+05 | 2.73E+02 | 1.17E+05 | 1.05E+05 |
| Meta168 | 4.48E+02 | C21H20O11 | HoMoorientin | Flavonoids | Flavonoid carbonoside | 4261-42-1 | B | 447.09, 285.04, 284.03, 284.05, 327.05 | 7.85E+04 | 2.49E+03 | 3.20E+05 | 6.47E+04 | 1.16E+05 | 9.43E+04 |
| Meta306 | 1.52E+02 | C5H12O5 | Xylitol | Others | Saccharides and Alcohols | 87-99-0 | B | 151.06, 59.01, 71.01, 89.03, 101.02 | 7.04E+04 | 9.00E+00 | 3.90E+05 | 9.00E+00 | 1.15E+05 | 1.92E+04 |
| Meta182 | 4.22E+02 | C12H23O14P | Trehalose 6-phosphate | Others | Saccharides and Alcohols | - | - | - | 1.05E+05 | 1.19E+05 | 1.06E+05 | 1.07E+05 | 1.09E+05 | 1.38E+05 |
| Meta92 | 2.68E+02 | C17H32O2 | Cis-10-Heptadecenoic Acid | Lipids | Free fatty acids | 29743-97-3 | A | 267.23, 266.41, 249.22, 266.4, 266.39 | 3.65E+04 | 2.19E+04 | 3.42E+05 | 3.65E+04 | 1.09E+05 | 8.75E+04 |
| Meta249 | 3.52E+02 | C21H36O4 | MAG(18:3)isomer2 | Lipids | Glycerol ester | - | A | 353.3, 261.1, 243.21, 121.1 | 2.58E+05 | 6.48E+04 | 9.00E+00 | 1.13E+05 | 1.09E+05 | 1.24E+05 |
| Meta97 | 1.66E+02 | C9H10O3 | 3-(4-Hydroxyphenyl)-propionic acid | Phenolic acids | Phenolic acids | 501-97-3 | B | 165.06, 59.01, 93.03, 121.07, 119.05 | 5.05E+04 | 9.06E+04 | 1.52E+05 | 1.41E+05 | 1.08E+05 | 1.15E+05 |
| Meta415 | 3.00E+02 | C16H12O6 | Chrysoeriol | Flavonoids | Flavonoid | 491-71-4 | B | 301.11, 269.08, 181.06, 226.06, 209.06 | 3.40E+05 | 2.84E+04 | 4.24E+04 | 1.21E+04 | 1.06E+05 | 2.09E+05 |
| Meta41 | 3.16E+02 | C16H12O7 | Isorhamnetin | Flavonoids | Flavonols | 480-19-3 | A | 315.05, 300.03, 315.05, 151, 271.03 | 1.60E+05 | 9.00E+00 | 2.61E+05 | 9.00E+00 | 1.05E+05 | 8.29E+04 |
| Meta13 | 5.95E+02 | C27H31O15 | Cyanidin -O-glucoside-O-rhamnoside | Flavonoids | Anthocyanins | - | B | 595.16, 287.06 | 1.30E+03 | 7.11E+03 | 3.83E+05 | 2.30E+04 | 1.04E+05 | 7.32E+04 |
| Meta54 | 6.30E+02 | C27H31ClO15 | Cyanidin 3-rutinoside(Keracyanin chloride) | Flavonoids | Anthocyanins | 18719-76-1 | B | 595, 287, 433.1 | 9.00E+00 | 6.82E+03 | 3.83E+05 | 1.81E+04 | 1.02E+05 | 8.44E+04 |
| Meta275 | 3.60E+02 | C15H20O10 | Syringic acid O-glucoside | Phenolic acids | Phenolic acids | - | A | 359.1, 182.1, 197.05, 138.09 | 1.60E+05 | 1.04E+05 | 2.85E+04 | 1.14E+05 | 1.02E+05 | 8.58E+04 |
| Meta129 | 4.78E+02 | C22H22O12 | Petunidin 3-O-glucoside | Flavonoids | Anthocyanins | 6988-81-4 | A | 479, 317 | 9.00E+00 | 1.78E+03 | 3.81E+05 | 9.00E+00 | 9.58E+04 | 1.01E+05 |
| Meta388 | 4.16E+02 | C22H24O8 | Apigenin-3-O-α-L-rhamnoside | Flavonoids | Flavonoid | - | A | 415.1, 161, 269.1 | 2.38E+05 | 4.63E+04 | 8.34E+04 | 1.56E+04 | 9.58E+04 | 9.41E+04 |
| Meta409 | 6.10E+02 | C27H30O16 | Luteolin-7,3'-Di-O-β-D-Glucoside | Flavonoids | Flavonoid | 257-724-7 | B | 609.15, 609.14, 301.04, 300.03, 607.25 | 2.72E+05 | 5.76E+04 | 3.79E+04 | 1.58E+04 | 9.57E+04 | 3.04E+06 |
| Meta245 | 1.17E+02 | C8H7N | Indole | Alkaloids | Plumerane | 120-72-9 | A | 118.07, 58.07, 59.08, 91.06, 117.06 | 1.64E+05 | 4.33E+04 | 1.44E+05 | 2.85E+04 | 9.51E+04 | 1.10E+05 |
| Meta106 | 3.45E+02 | C10H12N5O7P | Guanosine 3',5'-cyclic monophosphate | Nucleotides and derivatives | Nucleotides and derivatives | 7665-99-8 | A | 344.04, 150.04, 133.02, 78.96, 192.99 | 1.73E+05 | 3.22E+04 | 1.57E+05 | 1.75E+04 | 9.49E+04 | 8.98E+04 |
| Meta385 | 5.34E+02 | C24H22O14 | Isorhamnetin 3-O-β-(2''-O-acetyl-β-D-glucuronide) | Flavonoids | Flavonols | - | A | 535.11, 287.06, 345.06, 315.05, 369.06 | 3.08E+05 | 9.00E+00 | 7.15E+04 | 9.00E+00 | 9.49E+04 | 8.35E+04 |
| Meta151 | 1.61E+02 | C6H11NO4 | DL-2-Aminoadipic acid | Alkaloids | Alkaloids | 542-32-5 | B | 162.08, 98.06, 55.02, 56.05, 70.07 | 8.47E+04 | 1.33E+05 | 7.55E+04 | 8.20E+04 | 9.37E+04 | 6.77E+04 |
| Meta369 | 7.00E+02 | C32H44O17 | Olivin Diglucoside | Lignans and Coumarins | Lignans | - | B | 699.25, 375.15, 537.2, 179.07, 195.07 | 2.63E+05 | 3.39E+04 | 5.98E+04 | 9.55E+03 | 9.16E+04 | 9.38E+04 |
| Meta21 | 5.62E+02 | C28H34O12 | Pinoresinol-acetylglucose | Lignans and Coumarins | Lignans | - | B | 561.2, 357.13, 119.04, 59.01 | 3.11E+05 | 9.00E+00 | 3.47E+04 | 6.41E+03 | 8.81E+04 | 6.57E+04 |
| Meta56 | 4.93E+02 | C23H25O12 | Oenin chloride | Flavonoids | Anthocyanins | 7228-78-6 | B | 493.13, 331.08, 493.13, 315.05, 316.06 | 2.40E+04 | 1.43E+05 | 3.64E+04 | 1.47E+05 | 8.77E+04 | 5.02E+04 |
| Meta22 | 5.80E+02 | C28H36O13 | Syringaresinol-Hex | Lignans and Coumarins | Lignans | - | B | 579.21, 417.16, 519.31, 181.05 | 2.48E+05 | 3.26E+04 | 1.34E+04 | 5.52E+04 | 8.74E+04 | 7.33E+04 |
| Meta370 | 6.82E+02 | C32H42O16 | Pinoresinol diglucoside | Lignans and Coumarins | Lignans | 63902-38-5 | B | 681.24, 357.14, 519.19, 151.04, 161.05 | 2.68E+05 | 4.19E+04 | 3.75E+04 | 9.00E+00 | 8.69E+04 | 1.24E+05 |
| Meta260 | 2.96E+02 | C18H32O3 | 12,13-EODE | Lipids | Free fatty acids | 6799-85-5 | A | 295.23, 277.22, 195.14, 171.1, 183.1 | 2.60E+04 | 4.18E+04 | 1.39E+05 | 1.39E+05 | 8.64E+04 | 9.33E+04 |
| Meta140 | 5.50E+02 | C24H26N2O13 | Batanin | Alkaloids | Alkaloids | 7659-95-2 | A | 551.16, 303.04, 551.16, 533.14, 287.07 | 1.81E+05 | 9.09E+04 | 2.41E+04 | 4.84E+04 | 8.61E+04 | 9.58E+04 |
| Meta46 | 1.61E+02 | C9H7NO2 | Indole-5-carboxylic acid | Alkaloids | Plumerane | 1670-81-1 | B | 160.04, 116.05, 160.04, 115.7, 116.72 | 5.53E+04 | 8.21E+04 | 1.33E+05 | 6.64E+04 | 8.42E+04 | 6.63E+04 |
| Meta397 | 2.83E+02 | C17H17NO3 | N-z-p-Coumaroyl tyramine | Alkaloids | Phenolamine | - | B | 282.11, 119.05, 162.06 | 2.83E+03 | 2.32E+05 | 5.08E+02 | 9.35E+04 | 8.22E+04 | 5.03E+04 |
| Meta247 | 3.52E+02 | C21H36O4 | MAG(18:3)isomer4 | Lipids | Glycerol ester | - | B | 353.3, 261.1, 243.21, 121.1 | 2.95E+04 | 6.54E+03 | 2.67E+05 | 1.91E+04 | 8.06E+04 | 8.88E+04 |
| Meta244 | 3.63E+02 | C10H14N5O8P | Guanosine 5'-monophosphate | Nucleotides and derivatives | Nucleotides and derivatives | 85-32-5 | B | 364.06, 152.06, 135.03, 135.04, 97.03 | 1.17E+04 | 1.17E+05 | 1.31E+04 | 1.78E+05 | 7.99E+04 | 4.71E+04 |
| Meta190 | 1.76E+02 | C6H8O6 | D-Glucurono-6,3-lactone | Others | Others | 32449-92-6 | B | 175.02, 85.03, 59.01, 57.04, 55.02 | 1.56E+05 | 1.34E+05 | 3.21E+03 | 2.64E+04 | 7.99E+04 | 1.29E+05 |
| Meta301 | 4.93E+02 | C23H25O12 | Malvidin 3-O-galactoside | Flavonoids | Anthocyanins | 30113-37-2 | B | 493.13, 331.08, 493.13, 315.05, 316.06 | 2.33E+04 | 1.44E+05 | 3.54E+04 | 1.15E+05 | 7.93E+04 | 4.91E+04 |
| Meta176 | 2.08E+02 | C11H12O4 | Ethyl caffeate | Organic acids | Organic acids | 102-37-4 | B | 209.08, 145.03, 117.03, 177.06, 149.06 | 9.00E+00 | 9.00E+00 | 3.16E+05 | 9.00E+00 | 7.89E+04 | 4.97E+04 |
| Meta258 | 2.94E+02 | C18H30O3 | 9-KODE | Lipids | Free fatty acids | 54232-59-6 | B | 293.21, 275.2, 235.17, 113.1, 223.17 | 8.20E+03 | 6.41E+03 | 2.85E+05 | 1.09E+04 | 7.77E+04 | 7.79E+04 |
| Meta181 | 1.38E+02 | C8H10O2 | Tyrosol | Phenolic acids | Phenolic acids | 501-94-0 | B | 137.06, 137.06, 106.04, 119.05, 107.05 | 1.68E+04 | 1.97E+05 | 3.64E+04 | 5.94E+04 | 7.75E+04 | 5.27E+04 |
| Meta412 | 3.18E+02 | C20H30O3 | Hispanolone | Others | Others | 18676-07-8 | B | 319.22, 319.22, 301.21, 81.03, 95.05 | 5.76E+03 | 4.61E+03 | 2.89E+05 | 4.81E+03 | 7.60E+04 | 9.28E+04 |
| Meta65 | 1.82E+02 | C6H14O6 | D-Sorbitol | Others | Saccharides and Alcohols | 50-70-4 | B | 181.07, 59.01, 71.01, 89.03, 101.02 | 1.06E+05 | 5.03E+04 | 1.14E+05 | 2.89E+04 | 7.48E+04 | 8.53E+04 |
| Meta101 | 1.66E+02 | C9H10O3 | L-(-)-3-Phenyllactic acid | Organic acids | Organic acids | 20312-36-1 | A | 165.06, 59.01, 93.03, 121.07, 119.05 | 2.39E+04 | 6.45E+04 | 1.09E+05 | 9.92E+04 | 7.43E+04 | 7.66E+04 |
| Meta132 | 7.40E+02 | C33H40O19 | Robinin(Kaempferol-3-O-gal-rham-7-O-rham) | Flavonoids | Flavonols | 301-19-9 | A | 741.23, 433.11, 287.06, 595.17, 741.22 | 9.00E+00 | 1.06E+05 | 1.70E+05 | 1.54E+04 | 7.29E+04 | 3.56E+06 |
| Meta113 | 2.64E+02 | C13H16N2O4 | Phenylacetyl-L-glutamine | Amino acids and derivatives | Amino acids and derivatives | 28047-15-6 | B | 263.1, 145.06, 127.05, 128.04, 109.04 | 4.05E+04 | 2.19E+05 | 1.09E+04 | 1.63E+04 | 7.16E+04 | 5.10E+04 |
| Meta203 | 3.54E+02 | C21H38O4 | MAG(18:2)isomer1 | Lipids | Glycerol ester | - | A | 355.29, 263.24, 245.23, 337.27, 95.09 | 7.22E+04 | 1.71E+04 | 8.19E+04 | 1.13E+05 | 7.11E+04 | 7.48E+04 |
| Meta109 | 3.12E+02 | C18H20N2O3 | Phe-Phe | Amino acids and derivatives | Amino acids and derivatives | 2577-40-4 | B | 313.16, 120.09, 166.09, 103.05, 313.16 | 4.08E+04 | 2.99E+03 | 2.35E+05 | 5.56E+03 | 7.10E+04 | 4.19E+04 |
| Meta269 | 5.06E+02 | C23H22O13 | Quercetin O-acetylhexoside | Flavonoids | Flavonoid | - | B | 505.1, 301.2 | 2.27E+05 | 1.89E+04 | 2.65E+04 | 1.07E+04 | 7.09E+04 | 5.62E+04 |
| Meta357 | 1.61E+02 | C6H11NO4 | N-Acetylthreonine | Amino acids and derivatives | Amino acids and derivatives | 17093-74-2 | B | 162.08, 98.06, 55.02, 56.05, 70.07 | 1.01E+05 | 5.95E+04 | 8.66E+04 | 2.88E+04 | 6.89E+04 | 7.45E+04 |
| Meta127 | 1.32E+02 | C6H12O3 | 5-Hydroxyhexanoic acid | Organic acids | Organic acids | 185956-02-9 | B | 131.07, 85.07, 69.03, 113.06, 67.02 | 1.19E+05 | 4.59E+04 | 7.55E+04 | 3.01E+04 | 6.77E+04 | 5.42E+04 |
| Meta383 | 3.08E+02 | C20H36O2 | Eicosadienoic acid | Lipids | Free fatty acids | 5598-38-9 | A | 307.26, 307.19, 198.91, 289.25, 307.09 | 2.65E+04 | 1.95E+04 | 1.93E+05 | 2.73E+04 | 6.66E+04 | 5.36E+04 |
| Meta166 | 5.04E+02 | C18H32O16 | Panose | Others | Saccharides and Alcohols | 33401-87-5 | B | 503.17, 179.06, 161.05, 221.07, 101.02 | 2.60E+04 | 7.81E+04 | 5.53E+04 | 1.06E+05 | 6.64E+04 | 7.33E+04 |
| Meta67 | 2.40E+02 | C6H12N2O4S2 | L-(-)-Cystine | Amino acids and derivatives | Amino acids and derivatives | 56-89-3 | B | 241.03, 120.01, 151.98, 74.02, 180.97 | 5.33E+04 | 1.20E+05 | 5.14E+04 | 3.75E+04 | 6.56E+04 | 2.47E+04 |
| Meta254 | 2.00E+02 | C12H24O2 | Lauric acid | Lipids | Free fatty acids | 143-07-7 | A | 199.17, 199.17, 83.05, 109.03, 181.16 | 4.21E+04 | 1.04E+04 | 1.88E+05 | 1.43E+04 | 6.38E+04 | 1.34E+05 |
| Meta38 | 4.50E+02 | C21H22O11 | Eriodictyol 7-O-glucoside | Flavonoids | Dihydroflavone | 38965-51-4 | B | 451.12, 289.08, 163.04, 153.02, 179.03 | 7.28E+04 | 9.00E+00 | 1.81E+05 | 9.00E+00 | 6.35E+04 | 5.12E+04 |
| Meta201 | 3.50E+02 | C21H34O4 | MAG(18:4)isomer1 | Lipids | Glycerol ester | - | A | 351, 147.1, 259.21, 121.1 | 8.74E+04 | 2.75E+03 | 7.69E+04 | 8.20E+04 | 6.23E+04 | 5.56E+04 |
| Meta335 | 1.78E+02 | C6H10O6 | L-Gulonic-γ-lactone | Others | Saccharides and Alcohols | 1128-23-0 | B | 177.04, 59.01, 71.01, 57.03, 141.87 | 1.28E+05 | 9.00E+00 | 1.12E+05 | 5.81E+03 | 6.15E+04 | 4.64E+04 |
| Meta95 | 1.52E+02 | C5H12O5 | D-Arabitol | Others | Saccharides and Alcohols | - | - | 151.06, 59.01, 71.01, 89.03, 101.02 | 9.84E+04 | 9.00E+00 | 1.47E+05 | 9.00E+00 | 6.14E+04 | 2.53E+04 |
| Meta44 | 1.80E+02 | C10H12O3 | Coniferyl alcohol | Phenolic acids | Phenolic acids | 458-35-5 | B | 179.07, 146.04, 164.05, 145.03, 147.05 | 5.07E+04 | 9.00E+00 | 1.91E+05 | 2.75E+03 | 6.10E+04 | 5.65E+04 |
| Meta36 | 2.90E+02 | C15H14O6 | Catechin | Flavonoids | Flavanols | 154-23-4 | B | 289.07, 289.07, 245.08, 203.07, 109.03 | 1.54E+04 | 1.31E+05 | 7.10E+03 | 8.00E+04 | 5.85E+04 | 2.90E+04 |
| Meta253 | 2.08E+02 | C11H12O4 | 3,4-Dimethoxycinnamic acid | Phenolic acids | Phenolic acids | 14737-89-4 | B | 209.08, 145.03, 117.03, 177.06, 149.06 | 9.00E+00 | 9.00E+00 | 2.31E+05 | 9.00E+00 | 5.78E+04 | 3.75E+04 |
| Meta423 | 3.19E+02 | C21H37NO | 2-(Dodecylamino)-3-phenyl-1-propanol | Others | Others | - | B | 320.3, 302.29, 320.3, 91.05, 131.09 | 4.15E+04 | 5.72E+04 | 3.16E+04 | 9.33E+04 | 5.59E+04 | 4.68E+04 |
| Meta413 | 6.08E+02 | C28H32O15 | Neodiosmin | Flavonoids | Flavonoid | 38665-01-9 | B | 609.18, 463.13, 301.07, 609.18, 129.05 | 1.07E+05 | 1.01E+04 | 6.50E+04 | 4.02E+04 | 5.56E+04 | 2.08E+05 |
| Meta401 | 4.76E+02 | C23H24O11 | 5,2'-Dihydroxy-7,8-dimethoxyflavone glycosides | Flavonoids | Flavonoid | - | B | 477.14, 315.09, 300.06, 477.14, 271.06 | 1.79E+05 | 1.52E+04 | 1.63E+04 | 7.76E+03 | 5.46E+04 | 5.31E+04 |
| Meta327 | 4.34E+02 | C21H22O10 | Isohemiphloin | Flavonoids | Flavonoid carbonoside | 3682-02-8 | B | 433.11, 313.07, 343.08, 433.11, 193.01 | 5.79E+04 | 2.69E+04 | 8.90E+04 | 3.93E+04 | 5.33E+04 | 3.76E+04 |
| Meta319 | 2.05E+02 | C9H19NO4 | Pantothenol | Others | Saccharides and Alcohols | 16485-10-2 | B | 206.14, 76.08, 58.07, 188.13, 74.02 | 2.46E+04 | 9.87E+03 | 1.60E+05 | 1.71E+04 | 5.29E+04 | 2.61E+04 |
| Meta344 | 7.85E+02 | C27H33N9O15P2 | Flavin adenine dinucleotide(FAD) | Nucleotides and derivatives | Nucleotides and derivatives | 146-14-5 | B | 784.15, 437.09, 346.06, 180.99, 408.01 | 9.26E+04 | 3.11E+04 | 5.02E+04 | 3.69E+04 | 5.27E+04 | 3.63E+04 |
| Meta313 | 1.36E+02 | C5H4N4O | Allopurinol | Nucleotides and derivatives | Nucleotides and derivatives | 315-30-0 | B | 137, 119, 110.04, 55.03 | 2.01E+04 | 1.17E+05 | 9.10E+03 | 6.36E+04 | 5.26E+04 | 2.35E+04 |
| Meta295 | 1.12E+02 | C4H4N2O2 | Uracil | Nucleotides and derivatives | Nucleotides and derivatives | 66-22-8 | A | 111.03, 80, 95.95, 68.02 | 5.26E+04 | 5.22E+04 | 4.47E+04 | 5.96E+04 | 5.23E+04 | 4.84E+04 |
| Meta156 | 4.32E+02 | C21H20O10 | Isovitexin | Flavonoids | Flavonoid carbonoside | 29702-25-8 | A | 431.1, 311.06, 341.07, 283.06, 323.06 | 1.94E+05 | 2.36E+03 | 9.46E+03 | 2.24E+03 | 5.19E+04 | 5.53E+04 |
| Meta302 | 4.93E+02 | C23H25O12 | Malvidin 3-O-glucoside(Oenin) | Flavonoids | Anthocyanins | 18470-06-9 | B | 493.13, 331.08, 493.13, 315.05, 316.06 | 1.67E+04 | 8.55E+04 | 2.05E+04 | 7.85E+04 | 5.03E+04 | 3.56E+04 |
| Meta174 | 1.94E+02 | C7H14O6 | D-Pinitol | Others | Others | 10284-63-6 | B | 193.07, 101.02, 71.01, 85.03, 73.03 | 1.08E+05 | 1.20E+04 | 6.60E+04 | 1.35E+04 | 4.99E+04 | 4.93E+04 |
| Meta144 | 1.78E+02 | C10H10O3 | Trans-4-Hydroxycinnamic Acid Methyl Ester | Phenolic acids | Phenolic acids | 19367-38-5 | B | 177, 145, 117.04 | 3.53E+04 | 6.54E+03 | 1.46E+05 | 1.05E+04 | 4.96E+04 | 3.75E+04 |
| Meta148 | 4.48E+02 | C21H20O11 | Orientin | Flavonoids | Flavonoid carbonoside | - | - | 447.09, 285.04, 284.03, 284.05, 327.05 | 4.73E+04 | 5.64E+03 | 4.52E+04 | 9.73E+04 | 4.89E+04 | 3.98E+04 |
| Meta89 | 2.42E+02 | C15H30O2 | Pentadecanoic Acid | Lipids | Free fatty acids | 1002-84-2 | A | 241.22, 240.48, 223.21, 225.03 | 2.86E+04 | 2.14E+04 | 1.17E+05 | 2.00E+04 | 4.68E+04 | 6.91E+04 |
| Meta333 | 1.82E+02 | C6H14O6 | Dulcitol | Others | Saccharides and Alcohols | 608-66-2 | B | 181.07, 59.01, 71.01, 89.03, 101.02 | 8.16E+04 | 3.25E+03 | 9.18E+04 | 5.07E+03 | 4.54E+04 | 2.24E+04 |
| Meta161 | 3.56E+02 | C16H20O9 | Gentiopicroside | Terpenoids | Sesquiterpenoids | - | - | - | 9.00E+00 | 1.04E+05 | 9.00E+00 | 7.73E+04 | 4.53E+04 | 4.60E+04 |
| Meta230 | 3.45E+02 | C14H19NO9 | 4-Pyridoxic acid O-hexoside | Others | Vitamin | - | - | - | 2.92E+04 | 7.05E+04 | 1.65E+04 | 6.25E+04 | 4.47E+04 | 4.52E+04 |
| Meta14 | 7.70E+02 | C34H42O20 | Rhamnetin-O-glucoside-O-rhamnoside-O-rhamnoside | Flavonoids | Flavonoid | - | B | 771.23, 463.12, 609.19, 301.07, 625.18 | 1.37E+05 | 5.91E+02 | 4.10E+04 | 1.74E+02 | 4.47E+04 | 1.03E+06 |
| Meta386 | 3.82E+02 | C20H30O7 | Cinncassiol A | Phenolic acids | Phenolic acids | 73599-11-8 | B | 381.18, 235.12, 101.02, 381.18, 161.05 | 4.97E+04 | 2.42E+04 | 5.45E+04 | 3.97E+04 | 4.20E+04 | 2.48E+05 |
| Meta86 | 1.32E+02 | C6H12O3 | (S)-(-)-2-Hydroxyisocaproic acid | Organic acids | Organic acids | 13748-90-8 | B | 131.07, 85.07, 69.03, 113.06, 67.02 | 9.67E+03 | 4.70E+03 | 1.08E+05 | 4.52E+04 | 4.20E+04 | 6.26E+04 |
| Meta272 | 3.01E+02 | C8H16NO9P | N-Acetyl-D-glucosamine 1-phosphate | Others | Others | - | B | 300.05, 78.96, 96.97, 199, 78.97 | 7.91E+04 | 4.74E+04 | 2.30E+04 | 8.37E+03 | 3.95E+04 | 5.33E+04 |
| Meta266 | 8.00E+02 | C34H40O22 | Chrysoeriol O-hexosyl-O-hexosyl-O-Glucuronic acid | Flavonoids | Flavonoid | - | B | 799.19, 431.1, 445.12, 311.06, 341.07 | 4.75E+03 | 2.16E+04 | 9.00E+00 | 1.07E+05 | 3.34E+04 | 1.61E+04 |
| Meta362 | 1.65E+02 | C6H7N5O | 7-Methylguanine | Nucleotides and derivatives | Nucleotides and derivatives | 578-76-7 | B | 166, 149.05, 124.05, 55.03 | 1.07E+04 | 2.60E+04 | 8.40E+04 | 1.26E+04 | 3.33E+04 | 2.89E+04 |
| Meta223 | 5.64E+02 | C26H28O14 | C-Hexosyl-apigenin O-pentoside | Flavonoids | Flavonoid carbonoside | - | B | 565.1, 433.3, 415.1, 313.07 | 9.00E+00 | 9.00E+00 | 1.32E+05 | 9.00E+00 | 3.30E+04 | 9.66E+05 |
| Meta364 | 1.78E+02 | C9H6O4 | 5,7-Dihydroxychromone | Others | Others | 31721-94-5 | B | 177.02, 177.02, 133.03, 91.02, 63.02 | 3.27E+04 | 5.07E+04 | 1.32E+04 | 3.36E+04 | 3.26E+04 | 1.94E+05 |
| Meta329 | 1.37E+02 | C7H9N2O | 3-Carbamyl-1-methylpyridinium(1-Methylnicotinamide) | Others | Others | - | - | - | 3.92E+04 | 3.53E+04 | 2.80E+04 | 2.66E+04 | 3.23E+04 | 3.31E+04 |
| Meta404 | 2.56E+02 | C15H12O4 | Pinocembrin | Flavonoids | Dihydroflavone | 480-39-7 | B | 257.1, 153, 131.05, 151.04 | 1.10E+05 | 3.01E+03 | 1.30E+04 | 3.02E+03 | 3.22E+04 | 3.23E+04 |
| Meta31 | 3.04E+02 | C15H12O7 | Taxifolin | Flavonoids | Dihydroflavonol | 480-18-2 | B | 303.05, 125.02, 285.04, 175.04, 217.05 | 8.12E+03 | 2.11E+04 | 7.71E+04 | 2.22E+04 | 3.21E+04 | 1.77E+05 |
| Meta227 | 1.43E+02 | C6H9NOS | 4-Methyl-5-thiazoleethanol | Others | Others | 137-00-8 | B | 144.05, 115.05, 143.07, 117.07, 127.05 | 2.08E+04 | 3.07E+04 | 5.37E+04 | 2.29E+04 | 3.20E+04 | 4.48E+04 |
| Meta57 | 6.30E+02 | C27H31ClO15 | Pelargonin chloride | Flavonoids | Anthocyanins | - | - | 595, 287, 433.1 | 9.00E+00 | 9.00E+00 | 9.00E+00 | 1.28E+05 | 3.20E+04 | 1.73E+04 |
| Meta399 | 3.30E+02 | C17H14O7 | Jaceosidin | Flavonoids | Anthocyanins | 18085-97-7 | B | 331.08, 316.06, 301.03, 168, 273.04 | 8.75E+04 | 4.65E+02 | 3.37E+04 | 3.20E+02 | 3.05E+04 | 3.60E+04 |
| Meta117 | 1.29E+02 | C5H7NO3 | 5-Oxoproline | Amino acids and derivatives | Amino acids and derivatives | 149-87-1 | B | 128.04, 128.04, 82.03, 82.02 | 2.49E+04 | 1.61E+04 | 7.33E+04 | 6.60E+03 | 3.02E+04 | 3.08E+04 |
| Meta216 | 6.54E+02 | C32H30O15 | Luteolin O-sinapoylhexoside | Flavonoids | Flavonoid | - | B | 655.17, 331.08, 493.13, 493.14, 571.22 | 1.12E+05 | 9.00E+00 | 2.38E+03 | 5.10E+03 | 3.00E+04 | 2.02E+04 |
| Meta408 | 6.08E+02 | C28H32O15 | Diosmetin-7-O-rutin | Flavonoids | Flavonoid | - | B | 609.18, 463.13, 301.07, 609.18, 129.05 | 9.00E+00 | 1.03E+04 | 6.74E+04 | 3.92E+04 | 2.92E+04 | 2.00E+05 |
| Meta159 | 5.96E+02 | C27H32O15 | Eriocitrin | Flavonoids | Dihydroflavone | 13463-28-0 | B | 595.17, 287.06, 151, 459.12, 135.04 | 9.00E+00 | 1.29E+04 | 9.15E+04 | 9.42E+03 | 2.85E+04 | 3.06E+04 |
| Meta173 | 4.74E+02 | C22H18O12 | Chicory acid | Phenolic acids | Phenolic acids | 70831-56-0 | B | 473.07, 149.01, 179.04, 311.04, 293.03 | 8.80E+04 | 9.00E+00 | 1.22E+04 | 6.12E+03 | 2.66E+04 | 8.69E+04 |
| Meta33 | 4.32E+02 | C21H20O10 | Vitexin | Flavonoids | Flavonoid carbonoside | - | - | 431.1, 311.06, 341.07, 283.06, 323.06 | 4.85E+04 | 9.93E+03 | 4.18E+04 | 5.09E+03 | 2.63E+04 | 2.27E+04 |
| Meta339 | 2.18E+02 | C8H14N2O5 | (5-L-Glutamyl)-L-amino acid | Amino acids and derivatives | Amino acids and derivatives | - | - | - | 3.82E+04 | 2.27E+04 | 2.54E+04 | 1.46E+04 | 2.53E+04 | 2.65E+04 |
| Meta34 | 2.84E+02 | C16H12O5 | Acacetin | Flavonoids | Flavonoid | 480-44-4 | B | 285.08, 270.06, 242.06, 153.02, 133.07 | 9.28E+04 | 4.93E+03 | 2.70E+03 | 9.00E+00 | 2.51E+04 | 1.97E+04 |
| Meta192 | 4.34E+02 | C20H18O11 | Quercetin-3-O-α-L-arabinopyranoside(guaijaverin) | Flavonoids | Flavonols | 22255-13-6 | B | 433.08, 300.03, 271.03, 255.03, 179 | 6.99E+04 | 8.35E+03 | 8.46E+03 | 9.97E+03 | 2.42E+04 | 2.67E+04 |
| Meta389 | 1.66E+02 | C10H14O2 | 1-(4-Methoxyphenyl)-1-propanol | Phenolic acids | Phenolic acids | 5349-60-0 | B | 165.09, 149.06, 150.07, 135.04, 138.03 | 5.70E+04 | 1.25E+04 | 1.23E+04 | 1.48E+04 | 2.42E+04 | 5.85E+04 |
| Meta50 | 2.84E+02 | C16H12O5 | Genkwanin | Flavonoids | Flavonoid | 437-64-9 | B | 285.08, 270.06, 242.06, 153.02, 133.07 | 6.75E+04 | 9.00E+00 | 2.81E+04 | 9.00E+00 | 2.39E+04 | 6.86E+04 |
| Meta226 | 7.12E+02 | C30H32O20 | Quercetin 7-O-malonylhexosyl-hexoside | Flavonoids | Flavonoid | - | - | - | 2.36E+04 | 3.19E+04 | 1.68E+03 | 3.31E+04 | 2.26E+04 | 1.47E+04 |
| Meta180 | 1.94E+02 | C8H10N4O2 | Caffeine | Alkaloids | Alkaloids | - | - | - | 2.31E+04 | 6.35E+03 | 4.14E+04 | 1.58E+04 | 2.16E+04 | 2.16E+04 |
| Meta152 | 1.82E+02 | C9H10O4 | Syringic Aldehyde | Phenolic acids | Phenolic acids | - | - | - | 1.90E+04 | 1.78E+04 | 2.05E+04 | 2.90E+04 | 2.16E+04 | 2.07E+04 |
| Meta265 | 7.40E+02 | C33H40O19 | Apigenin O-hexosyl-O-rutinoside | Flavonoids | Flavonoid | - | B | 741.23, 433.11, 287.06, 595.17, 741.22 | 6.03E+04 | 1.62E+04 | 3.76E+03 | 1.46E+03 | 2.04E+04 | 2.12E+04 |
| Meta305 | 5.04E+02 | C18H32O16 | D-(+)-Melezitose | Others | Saccharides and Alcohols | 597-12-6 | B | 503.17, 179.06, 161.05, 221.07, 101.02 | 5.04E+03 | 1.90E+04 | 3.33E+04 | 2.10E+04 | 1.96E+04 | 1.81E+04 |
| Meta231 | 5.07E+02 | C24H29NO11 | Pyridoxine O-feruloyl hexoside | Others | Vitamin | - | - | - | 5.53E+02 | 4.10E+04 | 9.63E+02 | 3.52E+04 | 1.94E+04 | 1.91E+04 |
| Meta391 | 2.88E+02 | C16H32O4 | 10,16-Dihydroxy-palmitic acid | Lipids | Free fatty acids | 3233-90-7 | B | 287.22, 269.22, 285.21, 141.13, 241.22 | 6.77E+03 | 4.62E+03 | 5.79E+04 | 7.84E+03 | 1.93E+04 | 1.67E+04 |
| Meta35 | 4.46E+02 | C21H18O11 | Baicalin | Flavonoids | Flavonoid | 21967-41-9 | B | 447.09, 271.07, 271.09, 270.23, 272.05 | 9.00E+00 | 1.23E+03 | 3.77E+03 | 7.10E+04 | 1.90E+04 | 1.83E+04 |
| Meta299 | 5.78E+02 | C27H30O14 | Apigenin 7-rutinoside(Isorhoifolin) | Flavonoids | Flavonoid | - | - | 579.17, 271.07, 271.08, 433.11, 270.23 | 4.14E+04 | 9.00E+00 | 3.14E+04 | 3.10E+03 | 1.90E+04 | 2.42E+04 |
| Meta312 | 1.39E+02 | C6H5NO3 | 6-Hydroxynicotinic acid | Alkaloids | Alkaloids | 5006-66-6 | B | 140.03, 122.02, 78.04, 94.03, 51.02 | 9.00E+00 | 1.09E+04 | 6.25E+04 | 9.00E+00 | 1.84E+04 | 6.39E+04 |
| Meta267 | 4.32E+02 | C21H20O10 | Apigenin 7-O-glucoside(Cosmosiin) | Flavonoids | Flavonoid | 578-74-5 | B | 431.1, 311.06, 341.07, 283.06, 323.06 | 3.34E+04 | 2.08E+04 | 6.03E+03 | 1.12E+04 | 1.78E+04 | 2.60E+04 |
| Meta291 | 2.16E+02 | C8H16N4O3 | N-α-Acetyl-L-arginine | Amino acids and derivatives | Amino acids and derivatives | 155-84-0 | B | 217.13, 158.08, 70.07, 116.07, 112.08 | 4.01E+04 | 9.00E+00 | 2.90E+04 | 9.00E+00 | 1.73E+04 | 2.36E+04 |
| Meta400 | 3.88E+02 | C20H20O8 | 5-Hydroxy-6,7,8,3',4'-pentamethoxyflavone | Flavonoids | Flavonoid | 2174-59-6 | B | 389.13, 359.08, 374.1, 331.08, 341.07 | 2.61E+04 | 4.27E+04 | 2.62E+02 | 9.99E+01 | 1.73E+04 | 1.98E+04 |
| Meta406 | 5.48E+02 | C25H24O14 | Diosmetin-7-O-(6'-O-malonyl)-β-D-glucoside | Flavonoids | Flavonoid | - | B | 549.12, 301.07, 463.12, 286.05, 531.1 | 4.73E+04 | 9.00E+00 | 2.08E+04 | 9.00E+00 | 1.70E+04 | 1.55E+04 |
| Meta200 | 2.16E+02 | C12H24O3 | 12-Hydroxydodecanoic acid | Lipids | Free fatty acids | - | - | - | 2.00E+04 | 1.28E+04 | 1.95E+04 | 1.14E+04 | 1.59E+04 | 1.57E+04 |
| Meta131 | 1.48E+02 | C9H8O2 | p-Coumaraldehyde | Phenolic acids | Phenolic acids | 2538-87-6 | B | 147.05, 147.05, 119.05, 129.04, 117.04 | 7.21E+03 | 3.21E+04 | 2.47E+03 | 2.15E+04 | 1.58E+04 | 1.64E+04 |
| Meta221 | 7.56E+02 | C33H40O20 | 8-C-Hexosyl-apigenin O-hexosyl-O-hexoside | Flavonoids | Flavonoid carbonoside | - | B | 757.2, 595.2, 739.21, 449.11 | 9.00E+00 | 4.59E+03 | 5.38E+04 | 3.61E+03 | 1.55E+04 | 3.98E+04 |
| Meta219 | 7.88E+02 | C34H44O21 | Hesperetin C-hexosyl-O-hexosyl-O-hexoside | Flavonoids | Dihydroflavonol | - | B | 789.1, 626.8, 609.15, 507.11 | 9.00E+00 | 9.00E+00 | 6.13E+04 | 9.00E+00 | 1.53E+04 | 1.54E+04 |
| Meta287 | 1.35E+02 | C4H9NO2S | DL-Homocysteine | Amino acids and derivatives | Amino acids and derivatives | - | - | - | 1.49E+04 | 1.58E+04 | 1.43E+04 | 1.25E+04 | 1.44E+04 | 1.70E+04 |
| Meta112 | 1.41E+02 | C2H8NO4P | O-Phosphorylethanolamine | Others | Others | - | - | - | 1.09E+04 | 1.93E+04 | 7.40E+03 | 1.77E+04 | 1.38E+04 | 1.17E+04 |
| Meta387 | 4.18E+02 | C22H26O8 | Syringaresinol | Lignans and Coumarins | Lignans | 21453-69-0 | B | 417.16, 181.05, 166.03, 387.11, 402.14 | 1.23E+04 | 7.47E+03 | 1.73E+04 | 1.53E+04 | 1.31E+04 | 1.12E+04 |
| Meta334 | 3.02E+02 | C14H6O8 | Ellagic acid | Others | Others | - | - | - | 1.08E+04 | 1.60E+04 | 1.17E+04 | 1.30E+04 | 1.29E+04 | 1.08E+04 |
| Meta213 | 5.34E+02 | C24H22O14 | Cyanidin 3-O-malonylhexoside | Flavonoids | Anthocyanins | - | B | 535.11, 287.06, 345.06, 315.05, 369.06 | 4.82E+04 | 4.08E+02 | 9.00E+00 | 9.00E+00 | 1.22E+04 | 9.52E+03 |
| Meta78 | 1.29E+02 | C5H7NO3 | L-PyroglutamicAcid | Amino acids and derivatives | Amino acids and derivatives | 98-79-3 | B | 128.04, 128.04, 82.03, 82.02 | 8.16E+03 | 1.03E+04 | 2.45E+04 | 1.85E+03 | 1.12E+04 | 1.78E+04 |
| Meta91 | 2.78E+02 | C18H30O2 | α-Linolenic Acid | Lipids | Free fatty acids | 463-40-1 | B | 277.22, 59.01, 259.21, 233.23, 205.2 | 5.84E+03 | 9.00E+00 | 3.40E+04 | 1.82E+03 | 1.04E+04 | 1.33E+04 |
| Meta142 | 1.32E+02 | C4H4O5 | Oxaloacetic acid | Organic acids | Organic acids | 328-42-7 | B | 131, 59.2 | 2.45E+04 | 9.00E+00 | 1.54E+04 | 1.09E+03 | 1.03E+04 | 2.86E+04 |
| Meta45 | 3.58E+02 | C20H22O6 | Pinoresinol | Lignans and Coumarins | Lignans | 487-36-5 | B | 357.14, 151.04, 136.02, 357.14, 342.11 | 3.41E+04 | 4.08E+03 | 9.00E+00 | 2.68E+03 | 1.02E+04 | 1.80E+04 |
| Meta88 | 1.29E+02 | C6H11NO2 | Pipecolinic acid | Organic acids | Organic acids | 535-75-1 | B | 130.09, 84.08, 55 | 6.88E+03 | 8.58E+03 | 2.30E+04 | 1.93E+03 | 1.01E+04 | 1.68E+04 |
| Meta345 | 1.63E+02 | C5H9NO5 | 4-Hydroxy-L-glutamic acid | Amino acids and derivatives | Amino acids and derivatives | 3913-68-6 | B | 162.04, 144.03, 99.01, 55.02, 72.01 | 7.88E+03 | 1.13E+04 | 1.21E+04 | 5.88E+03 | 9.29E+03 | 1.19E+04 |
| Meta85 | 1.44E+02 | C6H8O4 | 2,3-Dimethylsuccinic acid | Amino acids and derivatives | Amino acids and derivatives | 13545-04-5 | B | 143.03, 58.01, 67.02, 111.01, 69.03 | 1.32E+04 | 1.34E+03 | 1.95E+04 | 9.00E+00 | 8.53E+03 | 3.58E+04 |
| Meta48 | 2.57E+02 | C8H20NO6P | Choline alfoscerate | Lipids | PC | 28319-77-9 | B | 258.11, 104.11, 125, 184.07, 86.1 | 5.15E+03 | 9.00E+00 | 2.85E+04 | 9.00E+00 | 8.42E+03 | 1.77E+05 |
| Meta255 | 4.86E+02 | C21H26O13 | 4-hydroxycoumarin di-glucoside | Lignans and Coumarins | Coumarins | - | B | 485.1, 323.2, 161.04, 219.1 | 9.00E+00 | 1.17E+04 | 1.67E+03 | 6.37E+03 | 4.93E+03 | 4.43E+04 |
| Meta167 | 6.66E+02 | C24H42O21 | Maltotetraose | Others | Saccharides and Alcohols | 34612-38-9 | B | 665.21, 161.05, 341.11, 545.18, 179.06 | 6.34E+03 | 9.00E+00 | 6.32E+03 | 5.81E+03 | 4.62E+03 | 4.39E+04 |
| Meta378 | 4.38E+02 | C20H22O11 | 3-Hydroxy-5-Methylphenol-1-Oxy-β-D-(6'-Gallic Acyl)Glucose | Phenolic acids | Phenolic acids | - | - | - | 1.55E+04 | 9.00E+00 | 1.24E+03 | 2.58E+02 | 4.24E+03 | 1.45E+04 |
| Meta372 | 5.16E+02 | C25H24O12 | Isochlorogenic acid A | Phenolic acids | Phenolic acids | 2450-53-5 | B | 515.12, 353.09, 191.06, 179.04, 335.08 | 9.00E+00 | 7.97E+02 | 9.00E+00 | 1.23E+04 | 3.28E+03 | 9.64E+03 |
| Meta298 | 4.32E+02 | C21H20O10 | Kaempferol 7-O-rhamnoside | Flavonoids | Flavonols | 20196-89-8 | B | 431.1, 311.06, 341.07, 283.06, 323.06 | 9.00E+00 | 9.00E+00 | 6.18E+02 | 9.00E+00 | 1.61E+02 | 1.52E+04 |
| Meta222 | 6.26E+02 | C28H34O16 | 6-C-Hexosyl-hesperetin O-hexoside | Flavonoids | Flavonoid carbonoside | - | B | 627.1, 465.1, 447.09, 429.08 | 9.00E+00 | 9.00E+00 | 9.00E+00 | 9.00E+00 | 9.00E+00 | 7.62E+05 |
| Meta263 | 4.66E+02 | C24H18O10 | Cyanidin O-syringic acid | Flavonoids | Anthocyanins | - | B | 465.08, 285.1, 329.09, 241.03 | 9.00E+00 | 9.00E+00 | 9.00E+00 | 9.00E+00 | 9.00E+00 | 4.75E+04 |
| Meta398 | 6.24E+02 | C29H36O15 | Verbascoside | Phenolic acids | Phenolic acids | 61276-17-3 | B | 623.2, 461.2, 119.05 | 9.00E+00 | 9.00E+00 | 9.00E+00 | 9.00E+00 | 9.00E+00 | 4.59E+05 |

**Table S2**. Top 20 most abundant metabolites in fruit peel

| **Index** | **Molecular Weight (Da)** | **Formula** | **Compounds** | **Class I** | **Average** |
| --- | --- | --- | --- | --- | --- |
| Meta432 | 1.19E+02 | C8H9N | N-Benzylmethylene isomethylamine | Alkaloids | 4.90E+07 |
| Meta207 | 1.03E+02 | C5H13NO | Choline | Alkaloids | 2.66E+07 |
| Meta75 | 1.17E+02 | C5H11NO2 | L-Valine | Amino acids and derivatives | 6.03E+07 |
| Meta342 | 1.17E+02 | C5H11NO2 | DL-Norvaline | Amino acids and derivatives | 5.99E+07 |
| Meta411 | 2.04E+02 | C11H12N2O2 | Tryptophan | Amino acids and derivatives | 4.45E+07 |
| Meta171 | 1.17E+02 | C5H11NO2 | D-(-)-Valine | Amino acids and derivatives | 2.40E+07 |
| Meta325 | 6.24E+02 | C28H32O16 | Isorhamnetin 3-O-neohesperidoside | Flavonoids | 3.15E+07 |
| Meta381 | 6.10E+02 | C27H30O16 | Bioquercetin | Flavonoids | 2.21E+07 |
| Meta90 | 2.78E+02 | C18H30O2 | γ-Linolenic Acid | Lipids | 5.56E+07 |
| Meta157 | 2.84E+02 | C18H36O2 | Stearic Acid | Lipids | 3.52E+07 |
| Meta240 | 2.78E+02 | C18H30O2 | Punicic acid | Lipids | 3.07E+07 |
| Meta422 | 2.73E+02 | C16H35NO2 | Hexadecylsphingosine | Lipids | 2.41E+07 |
| Meta256 | 2.96E+02 | C18H32O3 | 9,10-EODE | Lipids | 2.31E+07 |
| Meta20 | 2.96E+02 | C18H32O3 | 9-Hydroxy-10,12-octadecadienoic acid | Lipids | 2.16E+07 |
| Meta19 | 2.96E+02 | C18H32O3 | 13-Hydroxy-9,11-octadecadienoic acid | Lipids | 2.14E+07 |
| Meta392 | 2.96E+02 | C18H32O3 | 9S-Hyroxy-10E,12E-octadecadienoic acid | Lipids | 2.11E+07 |
| Meta324 | 2.97E+02 | C11H15N5O3S | 5'-Deoxy-5'-(methylthio)adenosine | Nucleotides and derivatives | 3.28E+07 |
| Meta138 | 3.42E+02 | C12H22O11 | Galactinol | Others | 2.17E+07 |
| Meta58 | 3.54E+02 | C16H18O9 | Chlorogenic acid | Phenolic acids | 2.41E+07 |
| Meta368 | 3.16E+02 | C13H16O9 | Protocatechuic acid-4-glucoside | Phenolic acids | 2.19E+07 |

**Table S3.** Top 20 most abundant metabolites in fruit pulp

| **Index** | **Molecular Weight (Da)** | **Formula** | **Compounds** | **Class I** | **Class II** | **Average** |
| --- | --- | --- | --- | --- | --- | --- |
| Meta432 | 1.19E+02 | C8H9N | N-Benzylmethylene isomethylamine | Alkaloids | Alkaloids | 3.28E+07 |
| Meta29 | 2.02E+02 | C10H26N4 | Spermine | Alkaloids | Phenolamine | 2.64E+07 |
| Meta207 | 1.03E+02 | C5H13NO | Choline | Alkaloids | Alkaloids | 2.48E+07 |
| Meta75 | 1.17E+02 | C5H11NO2 | L-Valine | Amino acids and derivatives | Amino acids and derivatives | 7.33E+07 |
| Meta342 | 1.17E+02 | C5H11NO2 | DL-Norvaline | Amino acids and derivatives | Amino acids and derivatives | 7.20E+07 |
| Meta325 | 6.24E+02 | C28H32O16 | Isorhamnetin 3-O-neohesperidoside | Flavonoids | Flavonols | 3.02E+07 |
| Meta90 | 2.78E+02 | C18H30O2 | γ-Linolenic Acid | Lipids | Free fatty acids | 7.21E+07 |
| Meta422 | 2.73E+02 | C16H35NO2 | Hexadecylsphingosine | Lipids | Sphingolipids | 3.51E+07 |
| Meta240 | 2.78E+02 | C18H30O2 | Punicic acid | Lipids | Free fatty acids | 3.22E+07 |
| Meta256 | 2.96E+02 | C18H32O3 | 9,10-EODE | Lipids | Free fatty acids | 2.73E+07 |
| Meta157 | 2.84E+02 | C18H36O2 | Stearic Acid | Lipids | Free fatty acids | 2.27E+07 |
| Meta20 | 2.96E+02 | C18H32O3 | 9-Hydroxy-10,12-octadecadienoic acid | Lipids | Free fatty acids | 2.15E+07 |
| Meta426 | 4.95E+02 | C24H50NO7P | LysoPC(16:1) | Lipids | LPC | 1.89E+07 |
| Meta392 | 2.96E+02 | C18H32O3 | 9S-Hyroxy-10E,12E-octadecadienoic acid | Lipids | Free fatty acids | 1.77E+07 |
| Meta352 | 1.48E+02 | C6H12O4 | (Rs)-Mevalonic acid | Organic acids | Organic acids | 1.85E+07 |
| Meta138 | 3.42E+02 | C12H22O11 | Galactinol | Others | Saccharides and Alcohols | 3.26E+07 |
| Meta150 | 2.19E+02 | C9H17NO5 | D-Pantothenic Acid | Others | Vitamin | 1.81E+07 |
| Meta58 | 3.54E+02 | C16H18O9 | Chlorogenic acid | Phenolic acids | Phenolic acids | 4.45E+07 |
| Meta368 | 3.16E+02 | C13H16O9 | Protocatechuic acid-4-glucoside | Phenolic acids | Phenolic acids | 3.37E+07 |
| Meta261 | 3.16E+02 | C13H16O9 | 2,5-Dihydroxy benzoic acid O-hexside | Phenolic acids | Phenolic acids | 1.76E+07 |
