# Supplementary material for: Comparative Metabolic Profiling in Pulp and Peel of Green and Red Pitayas (Hylocereus polyrhizus and Hylocereus undatus) Reveals Potential Valorization in the Pharmaceutical and Food Industries
Source: Biomed Res Int. 2021 Mar 12;2021:6546170. doi: 10.1155/2021/6546170 (PMC7980772; doi:10.1155/2021/6546170)
Supplement: Supplementary 2 — Figure S1: graph of mass scan data collected over time (TIC) of MRM from sample 20-Meta2 (9-hydroxy-10,12-octadecadienoic acid). Figure S2: graph of mass scan data collected over time (TIC) of MRM from sample 26-Meta 26 (trigonelline). Figure S3: graph of mass scan data collected over time (TIC) of MRM from sample 30-Meta30 (baicalein). Figure S4: graph of mass scan data collected over time (TIC) of MRM from sample 37-Meta37 (tangeretin). Figure S5: graph of mass scan data collected over time (TIC) of MRM from sample 43-Meta43 (isoquercitrin). [file 6546170.f2.docx]

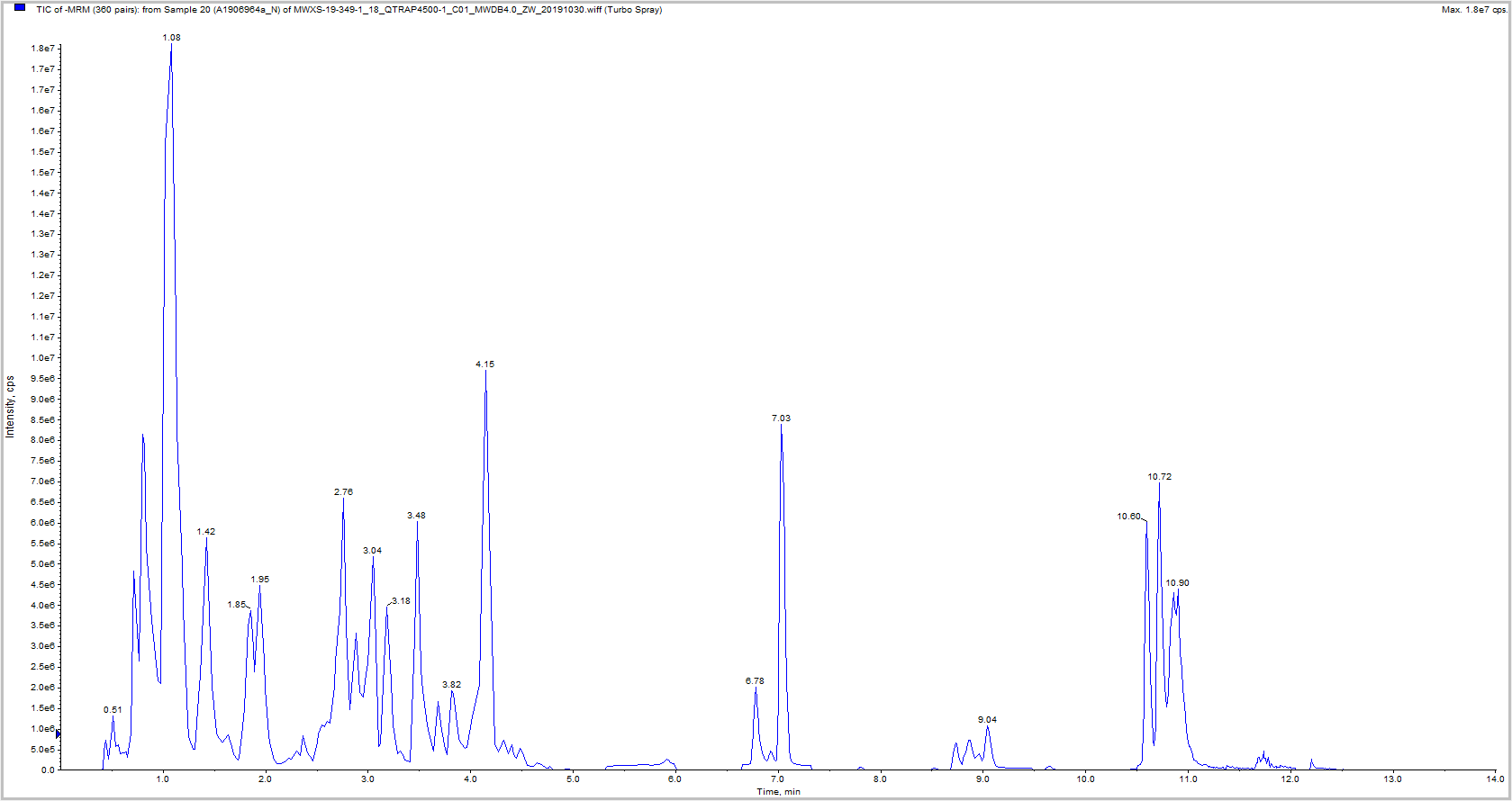


Figure S1: Graph of mass scan data collected over time (TIC) of MRM from sample 20-Meta20 (9-Hydroxy-10,12-octadecadienoic acid)


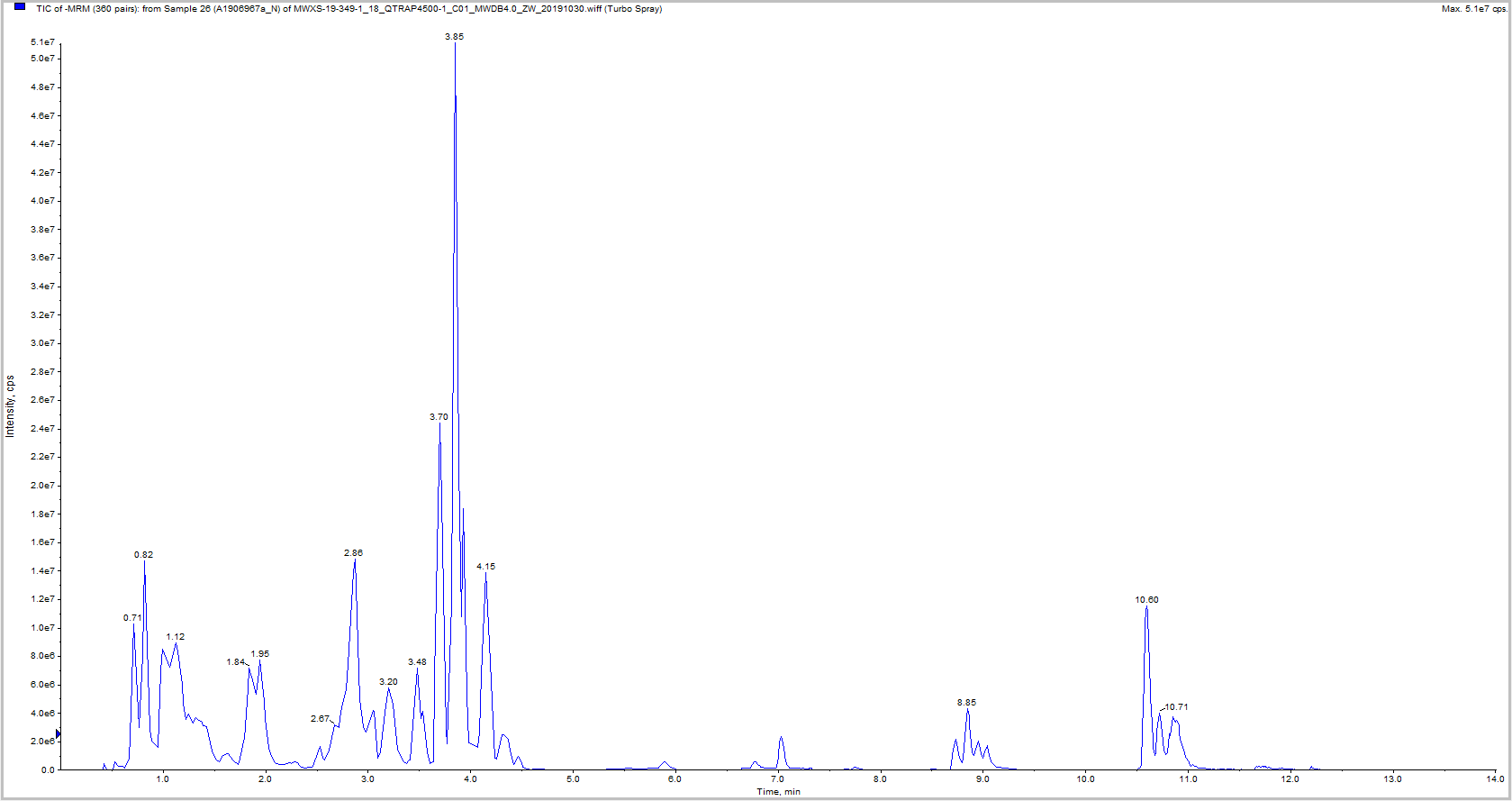


Figure S2: Graph of mass scan data collected over time (TIC) of MRM from sample 26-Meta 26 (Trigonelline)


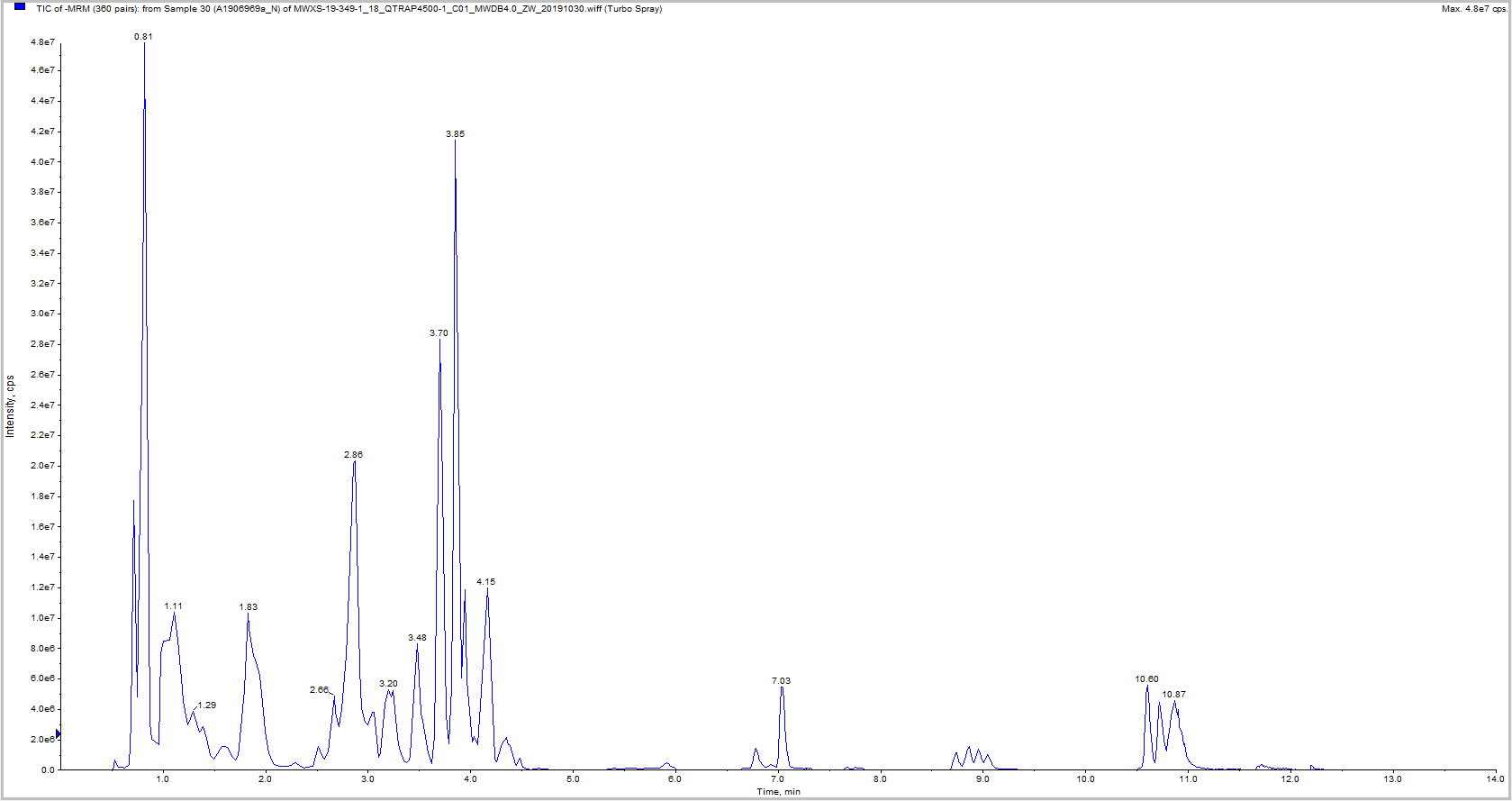


Figure S3: Graph of mass scan data collected over time (TIC) of MRM from sample 30-Meta30 (Baicalein)


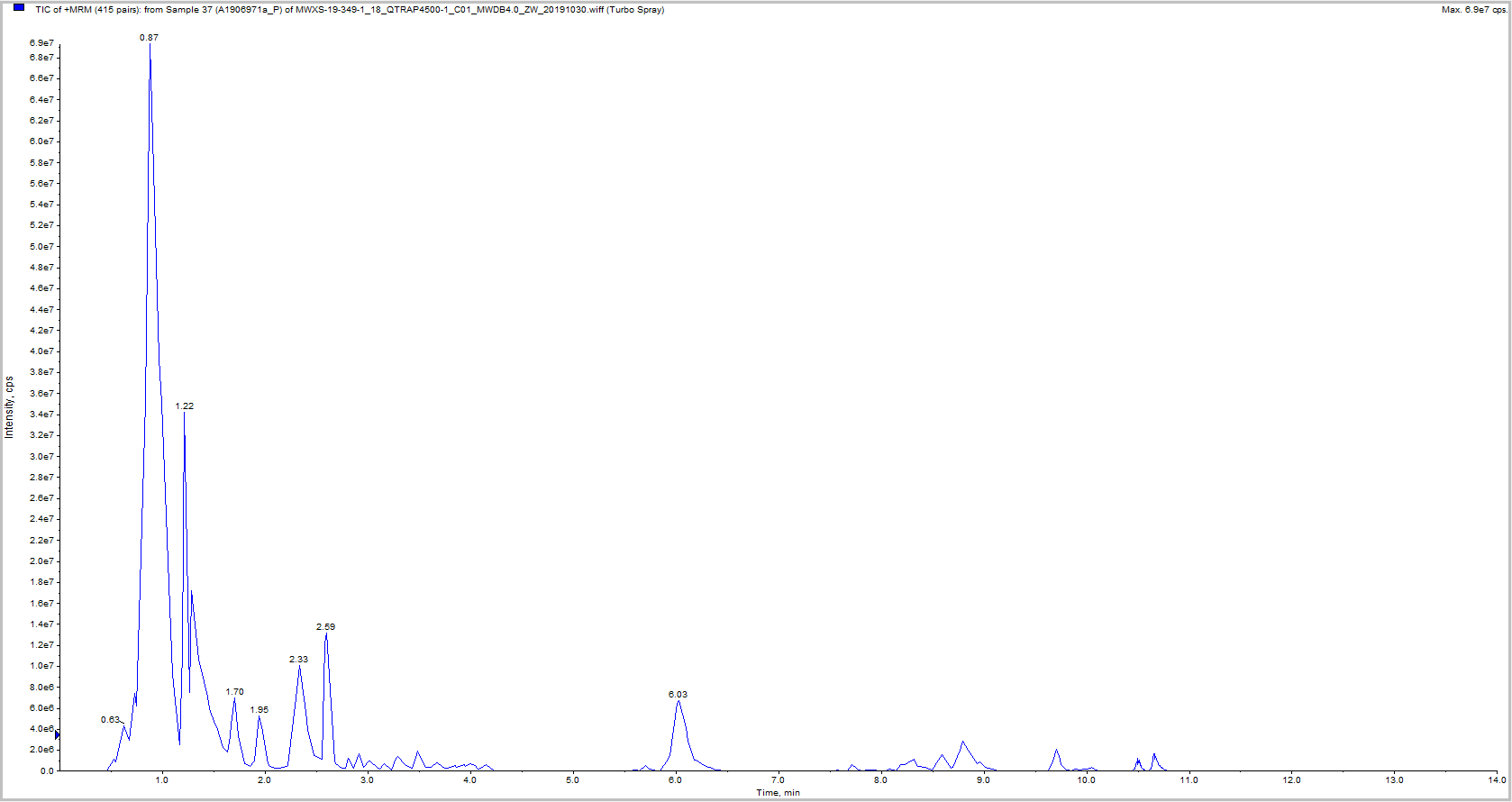


Figure S4: Graph of mass scan data collected over time (TIC) of MRM from sample 37-Meta37 (Tangeretin)


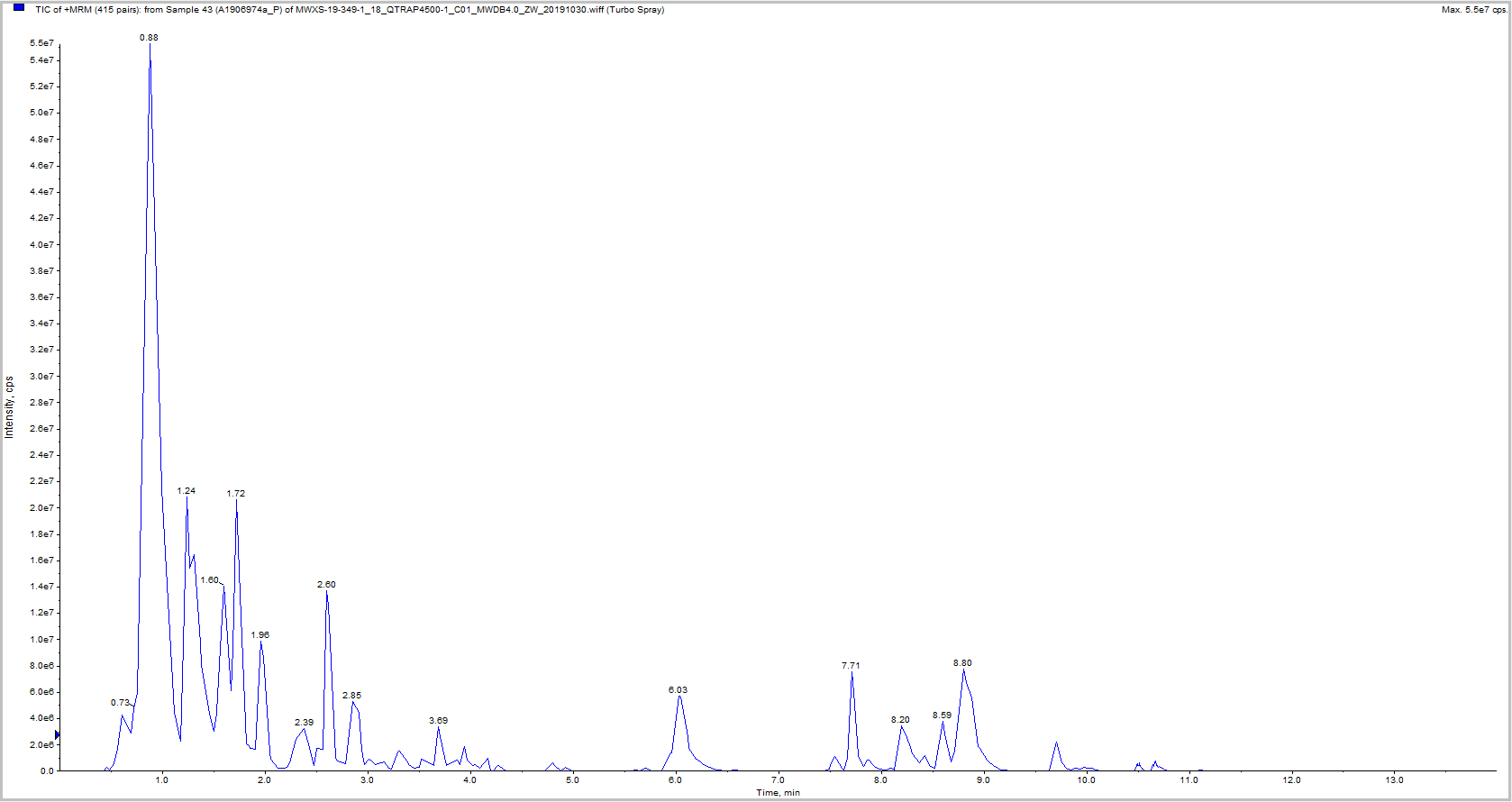


Figure S5: Graph of mass scan data collected over time (TIC) of MRM from sample 43-Meta43 (Isoquercitrin)
